# Whole genome sequencing, analyses of drug resistance-conferring mutations, and correlation with transmission of *Mycobacterium tuberculosis* carrying *katG*-S315T in Hanoi, Vietnam

**DOI:** 10.1038/s41598-019-51812-7

**Published:** 2019-10-25

**Authors:** Nguyen Thi Le Hang, Minako Hijikata, Shinji Maeda, Pham Huu Thuong, Jun Ohashi, Hoang Van Huan, Nguyen Phuong Hoang, Akiko Miyabayashi, Vu Cao Cuong, Shintaro Seto, Nguyen Van Hung, Naoto Keicho

**Affiliations:** 1 NCGM-BMH Medical Collaboration Center, Hanoi, Vietnam; 2 Department of Pathophysiology and Host Defense, The Research Institute of Tuberculosis, JATA, Tokyo, Japan; 3 Faculty of Pharmaceutical Sciences, Hokkaido University of Science, Hokkaido, Japan; 4 Hanoi Lung Hospital, Hanoi, Vietnam; 5 Department of Biological Sciences, Graduate School of Science, The University of Tokyo, Tokyo, Japan; 6 Department of Microbiology, Hanoi Lung Hospital, Hanoi, Vietnam; 7 Hanoi Department of Health, Hanoi, Vietnam; 8 Department of Microbiology, National Lung Hospital, Hanoi, Vietnam; 9 The Research Institute of Tuberculosis JATA, Tokyo, Japan; 10 National Center for Global Health and Medicine, Tokyo, Japan

**Keywords:** Bacterial genes, Tuberculosis

## Abstract

Drug-resistant tuberculosis (TB) is a serious global problem, and pathogen factors involved in the transmission of isoniazid (INH)-resistant TB have not been fully investigated. We performed whole genome sequencing of 332 clinical *Mycobacterium tuberculosis* (Mtb) isolates collected from patients newly diagnosed with smear-positive pulmonary TB in Hanoi, Vietnam. Using a bacterial genome-wide approach based on linear mixed models, we investigated the associations between 31-bp k-mers and clustered strains harboring *katG*-S315T, a major INH-resistance mutation in the present cohort and in the second panel previously published in South Africa. Five statistically significant genes, namely, *PPE18/19*, *gid*, *emrB*, *Rv1588c*, and *pncA*, were shared by the two panels. We further identified variants of the genes responsible for these k-mers, which are relevant to the spread of INH-resistant strains. Phylogenetic convergence test showed that variants relevant to *PPE46/47*-like chimeric genes were significantly associated with the same phenotype in Hanoi. The associations were further confirmed after adjustment for the confounders. These findings suggest that genomic variations of the pathogen facilitate the expansion of INH-resistance TB, at least in part, and our study provides a new insight into the mechanisms by which drug-resistant Mtb maintains fitness and spreads in Asia and Africa.

## Introduction

In 2017, 558,000 tuberculosis (TB) cases of multidrug resistance (MDR) or rifampicin (RMP) resistance were estimated globally^1^. Resistance to anti-TB drugs increases the burden of TB because the treatment of drug-resistant TB is generally prolonged and costly, while the outcome is relatively poor^2,3^. Isoniazid (INH) resistance without concurrent RMP resistance accounts for 7.1% of the new TB cases in the world^1^. The spread of INH resistance serves as a reservoir of more combined drug resistance^4,5^; empirical treatment after rapid genetic assessment of RMP resistance alone increases the future risk for developing MDR-TB or extensively drug-resistant (XDR)-TB^6,7^. INH-containing preventive therapy is also affected heavily when the INH-resistance rate is high in a target population.

Extensive studies have been performed to identify the genetic mutations responsible for *Mycobacterium tuberculosis* (Mtb) drug resistance, and comprehensive lists of drug-resistance-conferring mutations have been provided^8,9^. However, unidentified genetic variants, including compensatory mutations, may also assist or facilitate the transmission of drug-resistant TB without reducing the fitness of the bacilli^10^. Investigation of this mechanism would contribute to the effective control of drug-resistant TB, and the accumulation of molecular epidemiological data in many areas around the world would deepen the understanding of the dynamics.

Vietnam is one of the 30 TB high-burden countries, with approximately 124,000 incidence cases reported in 2017^1^. According to the national drug resistance survey conducted in 2011, among the new cases, resistance to any anti-TB drug accounted for 32.7% of the cases, and the proportion of INH-resistant TB reached 18.9%^11^. In our previous study cohort of 489 newly diagnosed patients in a city area, INH resistance was observed in 28.2% of the patients^12^, while RMP resistance remained in 4.9% of the patients. The predominant genetic mutations *katG-*S315T for INH and *rpoB-*S450L for RMP that confer drug resistance in Vietnam are similar to those reported in other Asian countries^13–15^. However, pathogen factors correlated with the transmission of INH-resistant TB have not been fully investigated.

Whole genome sequencing (WGS) has been recently used globally, offering new opportunities in the management of drug-resistant TB, since it can provide a huge amount of information, including genetic variants that are relevant to drug resistance throughout the genome^16^. WGS data also offer critical insights into the dynamics of TB endemics, transmission route, and the evolutionary patterns of genomic mutations^10,17–19^. Recently, bacterial genome-wide association studies (GWAS) controlling for population structure have also been performed for identifying the genes or genetic variants relevant to the TB phenotype, including drug resistance, by analyzing all single-nucleotide polymorphisms, small and large insertions/deletions (indels), or k-mers obtained from massive short-read data from next-generation sequencers (NGS)^20,21^.

In this study, we investigated drug resistance-conferring mutations carried by the clinical Mtb isolates from patients newly diagnosed with smear-positive pulmonary TB in Hanoi, Vietnam, by using WGS with a bacterial GWAS approach incorporating linear mixed models (LMMs). We then identified the genetic variants that may be relevant to the success in an extensive spread of INH-resistant strains, in reference to a previously published cohort study in KwaZulu-Natal, South Africa, as the second panel^19^.

## Results

### Prevalence of known drug resistance-conferring mutations

Among the 332 Hanoi samples analyzed with WGS, known mutations, which have been registered in the TBProfiler’s mutation database^9,22^, accounted for 80 (90.9%) of the 88 isolates with phenotypic INH resistance, 12 (92.3%) of the 13 isolates with RMP resistance, 63 (73.3%) of the 86 isolates with streptomycin (SM) resistance, 5 (100.0%) of the 5 isolates with ethambutol resistance, and 5 (55.6%) of the 9 isolates with pyrazinamide (PZA) resistance (Table 1). The most prevalent drug-resistance-conferring mutations were *katG-*S315T (26.2%) to INH, *rpsL-*K43R (13.3%) to SM, and *rpoB-*S450L (2.7%) to RMP. Mutations conferring resistance to the second-line drugs were *rrs-*A514C (4.5%) to amikacin and those in the *fabG1*-promoter (2.7%) to ethionamide. Others were rare mutations such as *ethR-*F110L to ethionamide, and *gyrA-*A90V and *gyrA-*D94G to fluoroquinolone (0.6%, 0.3%, and 0.6%, respectively). Among the 87 strains harboring *katG-*S315T mutations, 54 (62.1%) had at least one other drug-resistance-conferring mutation. Co-occurrence of *katG*-S315T and *rpsL*-K43R with or without other known mutations was observed most frequently (31 of the 87 isolates; 35.6%).

**Table 1:** Frequencies of known mutations conferring resistance to first-line drugs among all isolates, phenotypically resistant isolates, and susceptible isolates in the Hanoi sample set (N = 332).

Drug resistance-conferring mutations	Study population n (%)	Phenotypically resistant isolates n (%)	Phenotypically susceptible isolates n (%)
**Isoniazid**	N = 332	N = 88	N = 244
(−)	233 (70.2)	8 (9.1)	225 (92.2)
*katG* (S315T)	87 (26.2)	75 (85.3)	12 (4.9)
Rv1482c*-fabG1* (C-15T)	7 (2.1)	3 (3.4)	4 (1.6)
Rv1482c*-fabG1* (C-15T), *inhA* (S94A)	1 (0.3)	1 (1.1)	0 (0.0)
Rv1482c*-fabG1* (C-15T), *katG* (S315N)	1 (0.3)	1 (1.1)	0 (0.0)
*katG* (G234R)	1 (0.3)	0 (0.0)	1 (0.4)
*katG* (S315G)	1 (0.3)	0 (0.0)	1 (0.4)
*katG* (W191R)	1 (0.3)	0 (0.0)	1 (0.4)
**Streptomycin**	N = 332	N = 86	N = 246
(−)	259 (78.0)	23 (26.6)	236 (96.0)
*rpsL* (K43R)	44 (13.3)	42 (48.8)	2 (0.8)
*rrs* (A514C)	14 (4.2)	11 (12.8)	3 (1.2)
*rpsL* (K88R)	6 (1.8)	6 (7.0)	0 (0.0)
*gid* (L16R), *rrs* (A514C)	1 (0.3)	1 (1.2)	0 (0.0)
*rpsL* (K43R), *rrs* (A1401G)	1 (0.3)	1 (1.2)	0 (0.0)
*rrs* (A907T)	1 (0.3)	1 (1.2)	0 (0.0)
*rrs* (C517T)	2 (0.6)	1 (1.2)	1 (0.4)
*rrs* (T1239C)	1 (0.3)	0 (0.0)	1 (0.4)
*rpsL* (K88T)	1 (0.3)	0 (0.0)	1 (0.4)
*gid* (L16R)	2 (0.6)	0 (0.0)	2 (0.8)
**Rifampicin**	N = 332	N = 13	N = 319
(−)	310 (93.4)	1 (7.7)	309 (96.9)
*rpoB* (S450L)	9 (2.7)	4 (30.8)	5 (1.6)
*rpoB* (H445D)	4 (1.2)	4 (30.8)	0 (0.0)
*rpoB* (H445L)	2 (0.6)	2 (15.4)	0 (0.0)
*rpoB* (K446Q)	1 (0.3)	1 (7.7)	0 (0.0)
*rpoB* (Q432K)	1 (0.3)	1 (7.7)	0 (0.0)
*rpoB* (L452P)	1 (0.3)	0 (0.0)	1 (0.3)
*rposB* (S450L), *rpoB* (E761D)	1 (0.3)	0 (0.0)	1 (0.3)
*rpoB* (L430P)	3 (0.9)	0 (0.0)	3 (0.9)
**Ethambutol**	N = 332	N = 5	N = 327
(−)	303 (91.3)	0 (0.0)	303 (92.7)
*embB* (M306V)	6 (1.8)	3 (60.0)	3 (0.9)
*embB* (L370R)	13 (3.9)	1 (20.0)	12 (3.7)
*embC-embA* (C-12T)	1 (0.3)	1 (20.0)	0 (0.0)
*embB* (D354A)	1 (0.3)	0 (0.0)	1 (0.3)
*embB* (G406A)	2 (0.6)	0 (0.0)	2 (0.6)
*embB* (G406D)	1 (0.3)	0 (0.0)	1 (0.3)
*embB* (M306I)	4 (1.2)	0 (0.0)	4 (1.2)
*embB* (Q497R)	1 (0.3)	0 (0.0)	1 (0.3)
**Pyrazinamide**	N = 332	N = 9	N = 323
(−)	319 (96.1)	4 (44.4)	315 (97.5)
*pncA* (Q141P)	3 (0.9)	2 (22.2)	1 (0.3)
*pncA* (L159R)	1 (0.3)	1 (11.1)	0 (0.0)
*pncA* (S59P)	1 (0.3)	1 (11.1)	0 (0.0)
*pncA*-Rv2044c (T-11C)	3 (0.9)	1 (11.1)	2 (0.6)
*pncA* (A46V)	1 (0.3)	0 (0.0)	1 (0.3)
*pncA* (I31S)	1 (0.3)	0 (0.0)	1 (0.3)
*pncA* (Q10P)	1 (0.3)	0 (0.0)	1 (0.3)
*pncA* (Q10R)	1 (0.3)	0 (0.0)	1 (0.3)
*pncA* (S104R)	1 (0.3)	0 (0.0)	1 (0.3)

Other than SNVs, in one isolate, a 353-bp deletion was found in the *pncA* region after the screening of zero or low-depth areas, which also covered the *pncA* promoter and a part of the Rv2044c nearby. One sample had an in-frame 6-bp deletion (M434-D435) in the *rpoB* gene; this strain was not resistant to RMP. Eight isolates harboring 1-bp deletion in the *gid* gene, and two other isolates carrying 1-bp deletion of the *ethA* gene were also found (Supplementary Fig. S1).

### Mtb lineages/sublineages, drug-resistance-conferring mutations, and genetic clusters

The lineage (L)2 East Asian—mostly the Beijing strains—possessed the mutations that conferred drug resistance most frequently (52.6%); L1 (Indo-Oceanic; 23.2%) carried less and then L4 (Euro-American; 18.3%) the least (P < 0.0001) in Hanoi. The proportion of the above drug resistance was the highest in ancient Beijing strains (59.8%), followed by modern Beijing strains (40.9%), and then by non-Beijing strains (20.8%) (P < 0.0001).

**Figure 1:**
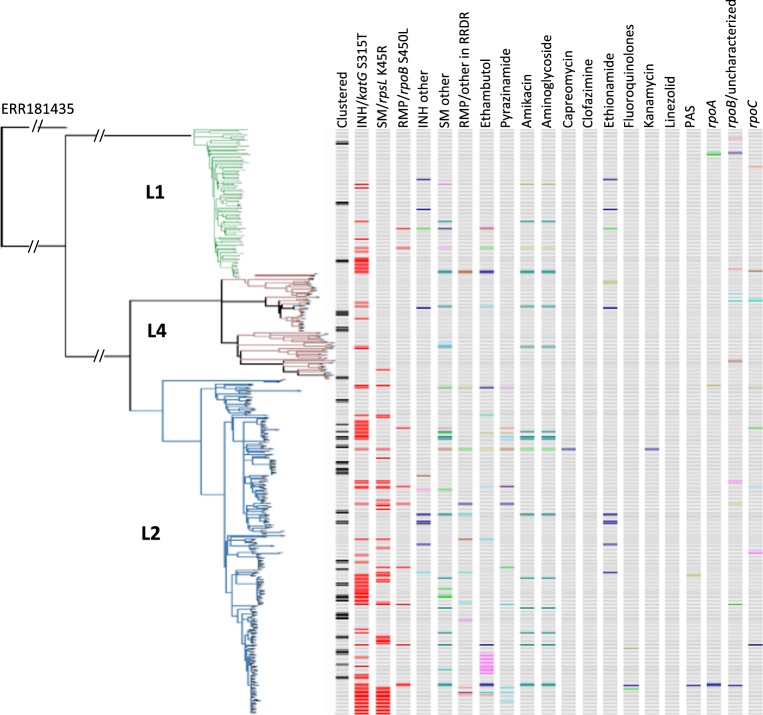
Distribution of drug resistant-relevant mutations and their presence in correlation with Mtb lineages and clustering. Clustered: defined by pairwise SNV difference <6 SNVs. Different colors indicate different mutations.

Figure 1 shows the distribution of the strains harboring mutations of interest. Of note, *katG*-S315T, known as a major INH-resistance mutation in the world^23^, was reported with a frequency of 26.2% in Hanoi, which accounted for 85.3% of the primary INH-resistance, and it was distributed unevenly among the strains like scattered islands. This S315T mutation, regardless of whether it occurred alone or in combination with other mutations, was more frequently observed in the L2 and L1 strains than in L4 (P < 0.0001) and also more in ancient Beijing than in modern Beijing and non-Beijing strains (59.0%, 40.9%, and 19.4%, respectively, P < 0.001). *katG*-S315T was often carried by ancient Beijing strains, whereas clustered strains defined by pairwise SNV differences of <6 alone were observed more frequently among modern Beijing strains than among ancient and non-Beijing strains (24.2%, 18.9%, and 9.7%, respectively, P = 0.016).

### Framework of bacterial GWAS analysis

To investigate the pathogen factors involved in the wide spread of INH-resistant strains in Hanoi, we used a combination of a representative INH resistance-conferring mutation *katG*-S315T (S315T[+]) with genetic cluster (cluster[+]) <6 SNV differences as a “phenotype” or objective variable for GWAS. *katG*-S315T and SNV-based genetic clusters alone served as phenotypes for comparison. Initially 31-bp k-mers throughout the genome were set as the “genotype” or explanatory variables, because the presence or absence of such k-mers provides clues for identifying the SNVs, indels, or even structural variants without a standard reference sequence for mapping reads^20^. When candidate genes were obtained from the k-mer analysis, individual variants, including SNVs and indels, were further analyzed for confirmation using other variant-based platforms.

### Association between phenotypes and 31-bp k-mers throughout the genome

**Figure 2:**
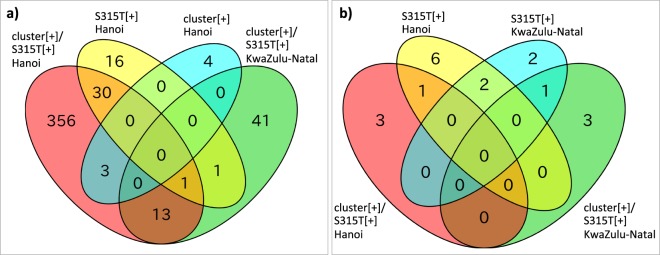
Venn diagram showing the number of genes significantly associated with the given phenotypes and shared by the two study cohorts. (**a**) results from k-mer-based GWAS; (**b**) results from phyOverlap; cluster[+]: clustered (pairwise SNV difference between two isolates is no more than five SNVs); S315T[+]: harboring *katG*-S315T mutation; Hanoi: Hanoi’s study cohort; KwaZulu-Natal: KwaZulu-Natal’s study cohort.

When k-mer GWAS was performed for our Hanoi samples, the number of genes harboring significant k-mers associated with cluster[+] alone and S315T[+] alone were 7 and 48, respectively, whereas the combined phenotype, cluster[+]/S315T[+] extracted 403 statistically significant genes, controlling for the population structure using LMM (Table 2). For comparison, 337 samples from the South African KwaZulu-Natal study were analyzed in a similar way as for the combined phenotype (Table 2), and 14 genes were eventually extracted in common (Fig. 2a). Of these, eight (*Rv1148c*, *mmpL6, PE_PGRS21*, *PE_PGRS53*, *pks12, rpoB*, *Rv2090*, and *wag22*) were excluded from further analysis because the presence or absence of k-mers appeared only in one sample of either case or control group. *PE_PGRS10* was further excluded since the presence of k-mers was not further confirmed by BLAST search for read sequences from both Hanoi and KwaZulu-Natal samples.

**Table 2:** Genes harboring k-mers significantly associated with different phenotypes (from (1) to (6)) in Hanoi and KwaZulu-Natal studies.

Phenotype						Gene name	Number of genes
(1)	(2)	(3)	(4)	(5)	(6)		
No	Yes	**Yes**	Yes	Yes	**Yes**	***gid***	**1**
No	Yes	No	Yes	No	Yes	*rrs*	1
No	Yes	Yes	No	Yes	No	*katG*	1
No	No	**Yes**	Yes	Yes	**Yes**	***PPE18, Rv1148c***	**2**
Yes	No	Yes	No	No	No	*ephB, glpK, mce1A*	3
No	No	**Yes**	No	No	**Yes**	***emrB, mmpL6, Rv1588c***	**3**
No	No	**Yes**	Yes	No	**Yes**	***PE_PGRS10, PE_PGRS21, PE_PGRS53, pks12, pncA, rpoB, Rv2090, wag22***	**8**
No	No	No	Yes	No	Yes	*atsD, fabG1, gyrA, helZ, murF, PE_PGRS52, Rv2000, Rv2161c, Rv3471c, ubiA*	10
No	No	No	Yes	Yes	Yes	*ctpB, drrA, ethA, fhaA, PPE19, PPE60, Rv0094c, Rv1945, Rv2019, Rv2141c, Rv3921c*	11
No	Yes	Yes	No	No	No	*accD2, eccC3, eccC4, fadE7, fas, glfT1, icd1, lepB, lprN, mbtJ, mce1R, menA, mez, PE_PGRS42, PPE30, PPE4, PPE54, rbsK, Rv1106c, Rv1147, Rv1215c, Rv1747, Rv2005c, Rv2566, Rv3085, Rv3401, Rv3677c, yfiH, zwf1*	29
No	No	No	No	Yes	No	*fadD5, Rv0336, Rv0348*	3
Yes	No	No	No	No	No	*atsA, gltA2, PE_PGRS12, Rv3863*	4
No	Yes	No	No	No	No	*mycP2, rpsL, Rv0012, Rv0219, Rv0893c, Rv1217c, Rv1443c, Rv1515c, Rv1516c, Rv2033c, Rv2277c, Rv2672, Rv2825c, Rv3177, Rv3618, yjcE*	16
No	No	No	No	No	Yes	*cysK1, espI, glmS, hsaA, lprQ, PE23, pks15, pks7, pmmB, rhlE, Rv0121c, Rv1073, Rv1639c, Rv2219, Rv3217c, Rv3254, Rv3660c, Rv3707c, thiL, vapB34*	20
No	No	Yes	No	No	No	*35kd_ag, accA3, adhE1, aglA, aldA, alkB, alr, amiB2, amiC, aprA, aroA, aroG, arsA, arsB2, bfrA, bfrB, blaC, cdh, cobN, cut1, cyp139, cysA2, cysD, deoC, devS, dinF, dinG, dinX, dnaB, dnaE2, dnaG, dnaK, dxs2, eccA3, eccB2, eccC2, eccD5, efp, egtE, eno, erg3, espH, esxJ, esxK, esxM, etgB, fadB3, fadB4, fadD15, fadD18, fadD22, fadD24, fadE18, fadE25, fadE34, fbiC, fecB, folC, frdD, fusA1, gatB, gcvB, glgA, gnd2, groEL2, guaB1, guaB2, hemB, hemY, hisH, hupB, hycQ, iniC, ispD, kshA, lepA, leuA, lipD, lipP, lppA, lppH, lppZ, lpqH, lprA, ltp1, mbtE, mce3B, mce3C, mcr7, menC, metH, mmpL1, mmpL11, mmpL5, mmsA, mmuM, moeB1, murE, mutT3, mymT, narG, nlhH, nrp, oppB, oxyR, papA2, papA5, PE_PGRS1, PE_PGRS13, PE_PGRS16, PE_PGRS17, PE_PGRS19, PE_PGRS2, PE_PGRS22, PE_PGRS23, PE_PGRS24, PE_PGRS25, PE_PGRS26, PE_PGRS27, PE_PGRS30, PE_PGRS31, PE_PGRS32, PE_PGRS33, PE_PGRS38, PE_PGRS4, PE_PGRS43, PE_PGRS44, PE_PGRS45, PE_PGRS46, PE_PGRS47, PE_PGRS48, PE_PGRS50, PE_PGRS54, PE_PGRS55, PE_PGRS56, PE_PGRS57, PE_PGRS6, PE_PGRS62, PE_PGRS7, PE_PGRS9, PE1, pepC, pepD, pepR, php, pknB, pknJ, pknK, pknL, pks13, pks9, pncB2, pntAb, ppa, PPE11, PPE12, PPE15, PPE21, PPE24, PPE34, PPE42, PPE46, PPE55, PPE56, PPE57, PPE59, PPE6, PPE8, ppm1, ppnK, ppsA, ppsC, proV, prpD, psd, purH, purL, purT, recC, relE, rfe, rimJ, rpfA, rpsD, Rv0057, Rv0060, Rv0074, Rv0095c, Rv0111, Rv0133, Rv0149, Rv0178, Rv0180c, Rv0188, Rv0192, Rv0193c, Rv0203, Rv0218, Rv0238, Rv0257, Rv0278c, Rv0302, Rv0323c, Rv0339c, Rv0367c, Rv0378, Rv0386, Rv0412c, Rv0457c, Rv0474, Rv0477, Rv0492c, Rv0537c, Rv0538, Rv0575c, Rv0584, Rv0647c, Rv0650, Rv0658c, Rv0686, Rv0696, Rv0740, Rv0790c, Rv0842, Rv0845, Rv0874c, Rv0876c, Rv0921, Rv0939, Rv0996, Rv1004c, Rv1024, Rv1043c, Rv1045, Rv1047, Rv1048c, Rv1050, Rv1069c, Rv1085c, Rv1178, Rv1225c, Rv1273c, Rv1313c, Rv1318c, Rv1372, Rv1395, Rv1425, Rv1455, Rv1488, Rv1498A, Rv1499, Rv1526c, Rv1610, Rv1667c, Rv1683, Rv1702c, Rv1707, Rv1714, Rv1725c, Rv1726, Rv1728c, Rv1729c, Rv1739c, Rv1749c, Rv1815, Rv1825, Rv1828, Rv1831, Rv1835c, Rv1879, Rv1895, Rv1941, Rv1999c, Rv2017, Rv2023A, Rv2024c, Rv2025c, Rv2038c, Rv2074, Rv2134c, Rv2143, Rv2164c, Rv2177c, Rv2190c, Rv2219A, Rv2228c, Rv2254c, Rv2256a, Rv2286c, Rv2314c, Rv2319c, Rv2327, Rv2424c, Rv2456c, Rv2532c, Rv2556c, Rv2567, Rv2621c, Rv2630, Rv2638, Rv2656c, Rv2668, Rv2669, Rv2729c, Rv2733c, Rv2792c, Rv2798c, Rv2851c, Rv2864c, Rv2893, Rv2897c, Rv2951c, Rv2974c, Rv2994, Rv3027c, Rv3060c, Rv3064c, Rv3074, Rv3104c, Rv3169, Rv3193c, Rv3201c, Rv3208, Rv3216, Rv3218, Rv3230c, Rv3263, Rv3365c, Rv3400, Rv3439c, Rv3549c, Rv3668c, Rv3689, Rv3717, Rv3728, Rv3729, Rv3807c, Rv3856c, Rv3896c, Rv3898c, sirA, smc, tal, TB9.4, tgs2, thiD, tig, treX, trxB1, uspC, uvrA, vapB36, vapC29, whiA, yrbE3A, yrbE3B*	356

Phenotype (1): clustered strains (cluster[+]) (pairwise SNV difference between two isolates is no more than five SNVs) in Hanoi’s study cohort; (2): strains harboring *katG*-S315 T mutation (S315 T[+]) in Hanoi’s cohort; (3): cluster[+]/S315T[+] in Hanoi’s cohort; (4): cluster[+] in KwaZulu-Natal’s cohort; (5) S315T[+] in KwaZulu-Natal’s cohort; (6): cluster[+]/S315T[+] in KwaZulu-Natal’s cohort; SNV: single nucleotide variant.

The genes in bold are associated with cluster[+]/S315T[+], and shared between Hanoi and KwaZulu-Natal study population.

**Figure 3:**
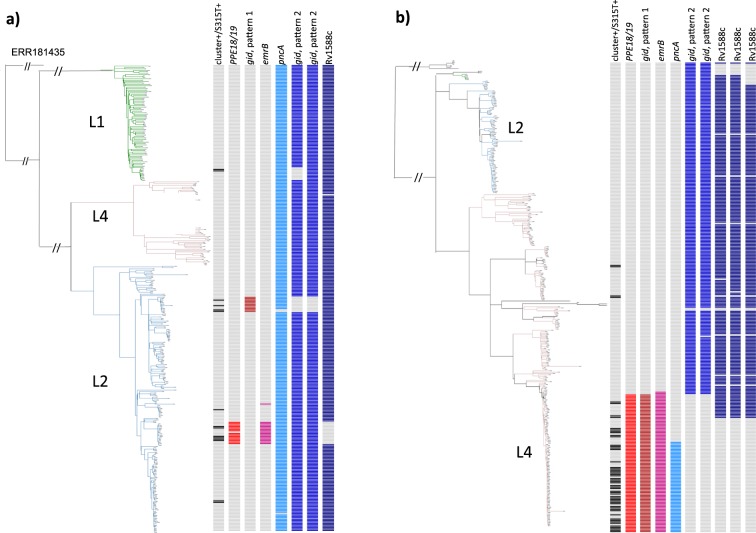
Distribution of k-mers derived from variant (or wild) types of five genes (*PPE18/19, gid, emrB, Rv1588c*, and *pncA*) that showed positive or negative associations with clustered strains carrying *katG*-S315T in the phylogenetic trees of Hanoi (**a**) and KwaZulu-Natal (**b**) study population.

The remaining five genes, namely, *PPE18*, *gid, emrB, Rv1588c*, and *pncA*, were confirmed by BLAST search and were aligned to the H37Rv reference sequence (Supplementary Fig. S2) and nominated as real candidates. In the Hanoi study population, significant k-mers included 36 k-mers from the *PPE18* gene (all had the same P value = 1.840E-09), 33 k-mers from *gid* (the best P value = 5.213E-08), 31 from *emrB* (8.579E-08), 4 from *Rv1588c* (8.579E-08), and 31 from the *pncA* gene (4.437E-07) (Supplementary Table S1), when 6.208E-06 was applied as the threshold of statistical significance after Bonferroni correction. For the KwaZulu-Natal study population, within the same set of genes, the best P values obtained from 5, 101, 31, 12, and 29 k-mers were 2.998E-08, 2.998E-08, 7.802E-07, 2.453E-06, and 4.802E-11, respectively, with the threshold of significance after Bonferroni correction as 4.591E-06 (Supplementary Table S1). Positive associations with the phenotype were mainly observed in L2 of Hanoi’s samples and L4 of KwaZulu-Natal’s samples (Fig. 3).

Next, we attempted to confirm significant k-mers by using a DBGWAS approach to identify all the relevant sequences as “unitigs” (Table 3). Consequently, one significant unitig indicating merged k-mers was identified in *PPE18*, three were identified *in gid*, two in *emrB*, and two in *Rv1588c* (the best q value in each gene was 5.562E-07, 3.676E-05, 1.704E-05, and 1.704E-05, respectively) for the Hanoi samples (Supplementary Table S2, Fig. S3). For the KwaZulu-Natal samples, one significant unitig was found in *PPE18*, seven in *gid*, two in *emrB*, and one in *pncA* (the best q value in each gene was 4.856E-10, 7.943E-09, 7.943E-09, and 7.639E-12, respectively) (Supplementary Table S2).

**Table 3:** Genes with k-mers significantly associated with clustered strains harboring *katG*-S315T mutation (cluster[+]/S315T[+]) in both Hanoi and KwaZulu-Natal study cohorts, further investigated by using the DBGWAS platform and variant/deletion-based search.

Gene name	Sample panel	K-mer-based GWAS	Strongest P value	DBGWAS*	Strongest P value	Variant-based GWAS/deletion-based GWAS/BLAST search	Strongest P value	Conclusion on variant(s) corresponding to significant k-mer(s)
*PPE18/19*	Hanoi	36 k-mers, SNV at 1339644 and 1339649	1.840E-09	1 unitig, SNVs at 1339644 and 1339649	5.56188E-07	SNVs at 1339644 (E99A) and 1339649 (A101T) found by variant-based GWAS	1.941E-09	Significant SNVs E99A and A101T
*PPE18/19*	KwaZulu-Natal	5 k-mers, SNV at 1340231	2.998E-08	1 unitig**, SNV at 1532777	4.8561E-10	SNV at 1532777 (Q286R) detected by BLAST search. By variant-based GWAS, SNV was not called properly	NA	SNV Q286R
*gid*	Hanoi	33 k-mers, SNV at 4407686	5.213E-08	3 unitgs, SNV at 4407686	3.676E-05	SNV at 4407686 (E173*) fount by variant-based GWAS	2.991E-06	Significant SNV E173*
*gid*	KwaZulu-Natal	101 k-mers	7.802E-07	7 unitigs, no SNV	7.943E-09	Large deletions (from 120 bp to 675 bp)	2.178E-04	Large deletions
*emrB*	Hanoi	31 k-mers, SNV at 876918	8.579E-08	2 unitigs, SNV at 876918	1.704E-05	SNV at 876918 (F508S) found by variant-based GWAS	1.507E-08	Significant SNV F508S
*emrB*	KwaZulu-Natal	31 k-mers, SNV at 877058	7.802E-07	2 unitigs**, SNV at 877058	7.943E-09	SNV at 877058 (I461I) found by variant-based GWAS	1.077E-06	Significant SNV I461I
*Rv1588c*	Hanoi	4 k-mers without SNV (neg.***)	8.579E-08	2 unitigs**, no SNV	1.704E-05	SNV at 1789735 (P34P) found by variant-based GWAS and BLAST search	1.511E-08	Significant SNV P34P
*Rv1588c*	KwaZulu-Natal	12 k-mers without SNV (neg.***)	2.453E-06	0 unitig	NA	No SNV inside the corresponding k-mers	NA	None
*pncA*	Hanoi	31 k-mers without SNV (neg.***)	4.437E-07	0 unitig (*pncA* not found)	NA	SNV at 2288820 (Q141P) found by variant-based GWAS	4.919E-07	Significant SNV Q141P
*pncA*	KwaZulu-Natal	29 k-mers, SNV at 2288785 and 2288788	4.802E-11	1 unitig, G insertion at 2288785	7.639E-12	SNV at 2288785 (T153fs) found by variant-based GWAS	6.569E-11	Significant SNV T153fs

cluster[+]: clustered (pairwise SNV difference between two isolates is no more than five SNVs); S315T[+]: harboring *katG*-S315T mutation; Hanoi: Hanoi’s study cohort; KwaZulu-Natal: KwaZulu-Natal’s study cohort; GWAS: genome wide association study; SNV: single nucleotide variant.

*Only significant unitigs and their SNVs are shown.

**Found by sequence search.

***Showing negative association with Cluster[+]/S315T[+] by logistic regression analyses

All k-mers annotated with *PPE18* in both study sets were mapped to either *PPE18, 19*, or 60. Nucleotide sequences within these three *PPE* genes were hardly distinguishable from each other (Supplementary Fig. S4); BWA-MEM mapping or simple BLAST search to the reference could not specify the exact *PPE* gene that each 31-bp k-mer belonged to. Nevertheless, the analysis of *de novo* assembled contigs demonstrated that the Hanoi variant k-mers were derived from *PPE18* and KwaZulu-Natal’s k-mers initially annotated with *PPE18* were derived from *PPE19*. Such a high degree of sequence similarity was also observed in significant unitigs identified in both study sets with the DBGWAS approach. Therefore, these k-mers and unitigs were designated as *PPE18/19* in our study (Table 3).

### Individual variant analysis using a genome-wide approach

When mapping the aforementioned significant k-mers of Hanoi samples on the H37Rv genome, we identified variants corresponding to the significant k-mers. Next, we attempted to confirm the variants by SNV- and small indel-based GWAS or BLAST search, and finally identified mutations encoding E99A and A101T, in *PPE18*, E173* (Glu173Stop) in the *gid*, F508S in *emrB*, P34P in *Rv1588c*, and Q141P in *pncA*. They were significantly associated with cluster[+]/S315T[+] (P values were 1.941E-09 for both *PPE18* SNVs, and the P values were 2.991E-06, 1.507E-08, 1.511E-08, and 4.919E-07 for *gid, emrB*, *Rv1588c*, and *pncA* SNV, respectively) (Table 3). The PROVEAN web server^24^ and SIFT_4G tool^25^ predicted *gid*-E173* and *emrB*-F508S as deleterious or that affecting the protein function.

For the KwaZulu-Natal samples, mapping results showed SNVs in *emrB* (I461I) and *pncA* (T153fs) (variant-based GWAS P values = 1.077E-06 and 6.596E-11, respectively). Initially, the SNV in the *PPE18/19* was not clearly mapped to the reference for the aforementioned reason. After the in-depth search for *de novo* assembled contigs, Q286R in *PPE19* was identified. Large deletions were found in *gid* in 101 of the 337 samples (from 120 to 675 bp). *Rv1588c* did not show any SNV in the corresponding area of the k-mers (Table 3).

SNVs and small indels from VCF files, significantly associated with the cluster[+]/S315T[+] of the Hanoi cohort obtained by GWAS, included 329 SNVs and indels other than the six shown above (Supplementary Table S3) (the best P = 1.754E-13, Supplementary Table S4). None of them were lineage-specific SNVs that have been reported elsewhere^22^. After further excluding variants in ambiguous *PE* and *PPE* genes, synonymous SNVs, a mutation causing S315T, and those with frequency no more than two in case or control samples, 144 SNVs and small indels were extracted in the samples from Hanoi. Of these, 77 SNVs correlated well with the principal component (PC)-7 (Supplementary Table S4), which corresponded to a phylogenetic branch of the ancient Beijing strains (Supplementary Fig. S5), with a high percentage (92.9%) of INH resistance.

We further analyzed the structural variants by detecting zero or low-depth areas when H37Rv and four complete genomes in Hanoi (AL123456, and AP018033 to AP018036) were used as references to be mapped. We found two groups of gene deletions associated with this phenotype. The first group included 1-bp deletion in *Rv1043c*, 52-bp deletion in *Rv2286c*, 3-bp insertion in *Rv0790c*, 1-bp deletion in *Rv3230c*, and a big deletion in *Rv2025c*, and these correlated well with PC-9 (Table 4), which corresponded to the same branch of ancient Beijing strains shown in SNV analysis. The second group included a 238-bp deletion in *accD2*, 2-bp deletion in *eis*, a 459-bp insertion in *Rv3077*, a 12-bp deletion in *Rv2690c*, and correlated with PC-12 (Table 4) corresponding to another branch of modern Beijing strains.

**Table 4:** Genes with deletions/insertions significantly associated with clustered strains harboring *katG*-S315T mutation among Hanoi samples obtained by GWAS, and corresponding k-mers.

Reference gene*	Locus in H37Rv (AL123456)	Deletion/insertion	P value	Principal component	K-mers	Strongest P value
		Details			No of significant k-mers/No of k-mers	
AL123456.3H37Rv|00828|*Rv0790c*	Rv0790c	3-bp insertion at 884090 to 884092	1.507E-08	9	42/46	1.43E-08
AL123456.3H37Rv|01092|*Rv1043c*	Rv1043c	1-bp deletion at 1166499	1.941E-09	9	45/61	1.43E-08
AL123456.3H37Rv|02117|*Rv2025c*	Rv2025c	Deletion of almost all Rv2025c gene, also Rv2024c (789-bp)	9.053E-08	9	819/833	1.43E-08
AL123456.3H37Rv|02393|*Rv2286c*	Rv2286c	52-bp deletion from 2559452 to 2559503	1.507E-08	9	69/123	1.43E-08
AL123456.3H37Rv|03394|*Rv3230c*	Rv3230c	1-bp deletion at 3607421	9.053E-08	9	59/63	1.84E-09
AL123456.3H37Rv|02535|*eis*	Rv2416c	2-bp deletion at 2714527–2714528	3.219E-06	12	0/60	1.32E-05
AP018033.1HN024|01030|*accD2*	Best hit with Rv0974c	238-bp deletion from 1086195 to 1086432	2.991E-06	12	—	—
AP018033.1HN024|02797|*HN024_02796*	Best hit with Rv2690c	12-bp deletion	1.720E-05	12	—	—
AP018036.1HN506|03217|*HN506_03216*	Best hit with Rv3077	459-bp insertion	1.720E-05	12	—	—

PC-9 correlated gene variants were observed in 14 samples (HN-042, HN-065, HN-120, HN-127, HN-152, HN-154, HN-170, HN-179, HN-186, HN-222, HN-253, HN-391, HN-441, HN-456) that belong to ancient Beijing sublineages.

PC-12 correlated gene variants were observed in 10 samples (HN-019, HN-075, HN-109, HN-137, HN-169, HN-324, HN-330, HN-435, HN-462, HN-506) that belong to modern Beijing sublineages.

*Genome sequences of clinical isolates in our Hanoi cohort, AP018033 to AP018036 as well as AL123456 (H37Rv genome) were used for reference.

### Convergence-based phyOverlap analysis

Seeking variants caused by convergent evolution is another alternative for detecting mutations supporting drug resistance, such as compensatory mutations^26^. We also tried to identify the phenotype-associated variants caused by convergence evolution. As expected, *rpoB* and *pncA* were also associated with *katG*-S315T mutation alone. However, no genes and inter-genic regions were significantly associated with the presence of cluster[+]/S315T[+] in both KwaZulu-Natal and our Hanoi panels (Table 5, Supplementary Table S5, Fig. 2b). One *PPE* gene, two *PE* genes, and one intergenic region, *PPE47, PE_PGRS55, PE_PGRS20* and *PE_PGRS3-PE_PGRS4* were extracted in the Hanoi samples only (Table 5).

**Table 5:** Genes detected by the phyOverlap method and their significant associations with the Hanoi strains harboring the *katG*-S315T mutation, with and without clustering, and loci shared with the KwaZulu-Natal study population.

Locus	Gene name	No of SNV	Parsimony score	P value	FDR (q value)	Remark
***Associated with cluster***[+]***/S315T***[+]						
Rv3021c	*PPE47*	12	73	0.00000	0.00000	PE/PPE
Rv3511	*PE_PGRS55*	13	18	0.00000	0.00000	PE-PGRS family protein
Rv0278c-Rv0279c	*PE_PGRS3-PE_PGRS4*	2	37	0.00002	0.02434	PE-PGRS family protein
Rv1068c	*PE_PGRS20*	13	27	0.00002	0.02434	PE-PGRS family protein
***Associated with S315T***[+]						
**Rv0667**	***rpoB***	**14**	**27**	**0.00000**	**0.00000**	**Rifampicin-resistance related gene**
Rv0682	*rpsL*	2	21	0.00000	0.00000	Streptomycin-resistance related gene
Rv0758	*phoR*	8	8	0.00002	0.01217	Sensor part of a two component regulatory system
**Rv2043c**	***pncA***	**10**	**11**	**0.00000**	**0.00000**	**Pyrazinamide-resistance related gene**
Rv3021c	*PPE47*	12	73	0.00000	0.00000	PE/PPE
Rv3169	*Rv3169*	5	5	0.00002	0.01217	Unknown
Rv3428c-Rv3429	*Rv3428c-PPE59*	28	247	0.00000	0.00000	
Rv3647c-Rv3648c	*Rv3647c-cspA*	4	4	0.00006	0.03245	
Rv3680-Rv3681c	*Rv3680-whiB4*	1	78	0.00000	0.00000	

The genes in bold are shared between Hanoi and KwaZulu-Natal study population.

*PPE46* and *PPE47* share large portions of identical nucleotide sequences. By BLAST search for variants in the *de novo* assembled contigs in addition to genome-wide screening of zero or low-depth areas (<15% of average depth) in the reference genome, we found that 37 (4 in L1, 19 in L2, and 14 in L4) strains had large deletions in *PPE47* (Supplementary Fig. S6) and all had fusion with *PPE46*, resulting in *PPE46*-like chimeric genes in 35 isolates and *PPE47*-like chimeric genes in 2 isolates (Supplementary Fig. S7). SNVs identified from 3379708 to 3379763 of AL123456.3 (H37Rv) in the *PPE47* region were the main reason for the significant association in the phylogenetic convergence test. In other candidates from phyOverlap, specific variants were not validated, presumably owing to the difficulties in mapping short reads followed by ambiguous base calling within *PE_PGRS* genes.

### Analyses using logistic regression models adjusted for host confounders in Hanoi samples

By multivariate analyses using conventional logistic regression models after adjustment for patients’ gender, age, living area, as well as Mtb lineages, all k-mers from *PPE18/19*, *emrB*, and a part of k-mers from *gid* showed positive associations with clustered strains carrying *katG*-S315T mutations in the Hanoi study population (adjusted odds ratio [aOR] with 95% confidence interval [CI] = 13.20 [3.49–49.96], 11.98 [3.24–44.29], and 12.42 [2.81–54.90], respectively), whereas *Rv1588c* and *pncA* k-mers showed negative associations (aOR with 95% CI = 0.08 [0.02–0.31], and 0.01 [0.00–0.25], respectively). All variants corresponding to significant k-mers showed positive associations with cluster[+]/S315T[+]. The *PPE46/47*-like chimeric gene also showed positive association with cluster[+]/S315T[+] (aOR with 95% CI = 6.81 [2.13–21.72]) (Supplementary Table S6).

## Discussion

We identified a variety of drug resistance-conferring mutations prevailing in Hanoi, northern Vietnam, which appeared most frequently in the East Asian Mtb lineage L2 particularly in ancient Beijing sublineage, and less in L1, and then least in L4. Large deletions that were not detected by conventional variant calling from mapped short reads were also found in *pncA*. Using the bacterial GWAS approach, we extracted candidate genes that were significantly associated with the clustered strains harboring the *katG*-S315T mutation and that were common to the two independent data sets—our cohort panel and a previous South African study, the KwaZulu-Natal cohort by Cohen *et al*.^19^—suggesting that expansions of INH-resistance TB can be facilitated by pathogen factors, at least in part.

A major drug-resistance conferring mutation, *katG*-S315T, accounted for 85.3% of the INH-resistance strains in our study population, which was similar to that reported in other studies in Vietnam, e.g., 73.2% was reported by Minh *et al*.^13^, 73.6% was reported by Huyen *et al*.^27^, 81.3% by Nguyen *et al*.^15^ and 78.4% was reported in Southeast Asian countries^28^. This *katG*-S315T is known as a mutation with low-fitness cost, spreads to Beijing strains and others, and is more likely to be clustered^23,29,30^. The acquisition of *katG*-S315T mutation precedes other resistance mutations, including RMP^4,5,19^. Indeed, this mutation occurs more frequently in MDR-TB strains than other mutations^30,31^, and it has an important implication in the transmission and control of MDR-TB.

In this setting, we performed bacterial GWAS with phenotypic combination of clusters defined by <6 SNVs^32^ and *katG*-S315T mutation as a surrogate, and searched for pathogenic variants contributing to the spread of INH-resistance. We identified five genes, namely, *PPE18/19*, *gid, emrB, Rv1588c*, and *pncA*, which were shared by two different sample panels in Asia and Africa with different major Mtb lineages, L2 and L4, respectively.

Of the five genes extracted from the k-mer GWAS, except for drug-resistance-conferring genes, the best P values were obtained from *PPE18/19* both in the Hanoi population and in the KwaZulu-Natal cohort. The association was mainly observed in L2 of Hanoi’s samples and L4 of KwaZulu-Natal’s samples. *PPE18/19* genes are members of a multigene family and share high sequence similarity with another PPE gene, *PPE60*^33^. Among these *PPE* genes, homologous recombination events frequently occur and contribute to the sequence diversity^34,35^. Among nonsynonymous SNVs and small indels detected in the *PPE18* gene of our study population leading to amino acid change in Mtb39A, two SNVs were significantly associated with cluster[+]/S315T[+], suggesting the spread of INH-resistant TB. Although their role in virulence is not fully understood, the PPE18 protein, also known as Mtb39A, was shown to downregulate the proinflammatory response and Th1-type immunity, interacting with host TLR2^36^, and facilitated survival and multiplication of Mtb bacilli in a mouse model^37^.

The T-cell epitopes in Mtb39A^38–40^ lead to strong T-cell proliferation and IFN-gamma production^38^. Therefore it is used as a subunit for the human TB vaccine candidate Mtb72F and its successor M72. It has been proved to be immunogenic and can stimulate both cellular and humoral immune response^41,42^. By comparing with the lists of the T-cell epitopes published elsewhere^38,40,43,44^, nonsynonymous mutations E99A and A101T in *PPE18* identified in the Hanoi population and Q286R in *PPE19* in the KwaZulu-Natal’s panel were both found to be included in the sequences acting as T-cell epitopes for cellular immunity. Despite the presence of *PPE18* variants in the Hanoi strains, the original T-cell epitope sequences were mostly conserved in either *PPE19* or *PPE60*, when the BLAST search was applied. Variants of these *PPE* genes associated with spread of INH-resistance strains may thus increase the antigenic diversity of the bacilli, which may help evade or exploit human immunity by unidentified mechanisms through the process of human-Mtb coevolution^33,35,45^.

Mutations in the *gid* gene have been reported to be associated with low-level resistance to SM^46^, which has been used as the first-line drug since a long time until recently. Because *gid* encodes methyltransferase that is responsible for the methylation of 16 S rRNA involved in the translational fidelity^47^, it is thus conceivable that the *gid* mutation may modulate the fitness of INH-resistance conferring mutations through the change in mRNA translational fidelity. Our study revealed that *gid* k-mers with E173* mutant were significantly associated with cluster[+]/S315T[+] and S315T[+], but not with cluster[+] alone, indicating that a concurrence of *gid*-E173* and *katG*-S315T may facilitate transmission even after controlling for population structure. The concurrence of *gid* 130-bp deletion and *katG*-S315T is the first step toward XDR-level drug resistance in Africa^19^. In our study population, all strains with *gid*-E173* mutation had at least one genetic mutation conferring resistance to first-line drugs. This non-sense mutation *gid*-E173* may facilitate the expansion of *katG*-S315T mutant strains. Indeed, other groups have reported that SM-resistant strains seem to be more clustered in Vietnam^48^.

Efflux pumps, including *emrB*, have been reported to be associated with pathogenicity since the up-regulation of efflux gene expression is involved in the development of resistance to anti-TB drugs^49^ and a wide array of physiologic processes such as the growth kinetics or transportation of a variety of compounds^50^. The combination of the *katG*-S315T mutant with *emrB* variant F508S in the Hanoi study may thus increase the drug efflux activity and facilitate Mtb survival and spread, by mitigating drug pressure. *emrB* (*Rv0783c*) belongs to the major facilitator superfamily (MFS) characteristically energized by the proton motive force (H^+^ or Na^+^)^50,51^, and may confer low-level resistance to RMP^52^. Although the role of I461I in *emrB* in KwaZulu-Natal’s population is unknown at present, this synonymous mutation (c.1383 C > A, ATC > ATA) is very rare in terms of codon usage of Mtb^53^, and the significance of codon usage bias and t-RNA modification should be taken into account, because rare codons are sometimes advantageous to the survival of Mtb under stress conditions^54^. When exposed to INH, various MFS efflux pump genes were reported to be overexpressed^50^, and these may induce sustained increased efflux activity with selection and stabilization of drug-resistant mutations^55^. This may also be relevant to the acquisition of additional drug resistance. Further studies are required for elucidating the function of the mutations in efflux pump genes. Indeed, SNVs in efflux pump genes are often found in XDR strains but not in drug-susceptible strains^51^, although it is often difficult to identify the extrusion of a drug to a specific gene^50,56^. The association pattern characterized by the presence or absence of the variant of *emrB* with the phenotype was quite similar to that of *PPE18/19*, whose phenomenon was shown beyond lineages, L2 in Hanoi, and L4 in KwaZulu-Natal. Although membrane localization of the *PPE* genes may be functionally linked to efflux pump activities, it is currently unknown.

*Rv1588c* is a partial REP13E12 repeat protein^57^. Although k-mers carrying the reference sequences in *Rv1588c* showed negative association with the clustered strains harboring *katG*-S315T in the two panels, their functional significance remains unclear. As a variant, only a synonymous variant P34P was found in Hanoi, which was associated with ancient Beijing sublineage.

The reference sequence (=wild type) k-mers in *pncA* were also associated negatively with the phenotype in the Hanoi cohort, and variant-carrying k-mers showed a positive association in the KwaZulu-Natal cohort. As a variant found in Hanoi, Q141P in *pncA* has been reported as a high-confidence mutation leading to PZA resistance^58,59^. PZA resistance is often observed among MDR-TB isolates^60,61^. Thus, the possibility of *pncA* mutations facilitating the transmission of *katG*-S315T mutant Mtb isolates may make TB management more challenging.

Convergence-based phyOverlap analysis, which is a different approach, revealed that four different gene/intergenic regions only in the Hanoi study population that may have been caused by convergence evolution were significantly associated with the clustered strains carrying the *katG*-S315T mutation; and these four were present in the *PE or PPE* region. The impact of genetic variation on the function of PE or PPE proteins remains largely unknown^35^. However, at least large deletions between *PPE46* and *PPE47* genes were observed in all three lineages (L1, L2, and L4) in Hanoi, and these were positively associated with the spread of INH-resistant TB. The deletion between the identical sequences of the two *PPE* genes leads to in-frame gene fusions through homologous recombination^62^, and a relatively high prevalence, indicating a clonal expansion of Haarlem strains (L4) in Tunisia^34^, suggested that the generation of the new chimeric genes may facilitate antigenic diversity and provide new determinants for pathogen’s virulence across the lineages.

Further analyses using logistic regression models confirmed that all variants corresponding to significant k-mers of the five genes and even variants detected by the phylogenetic convergence test in Hanoi samples were positively associated with the spread of INH-resistant TB, even after adjustment for other possible confounders.

Our study has some limitations. First, we were not able to trace the epidemiological link among the patients to corroborate the transmission chain. Hanoi is the capital city of Vietnam with on-going urbanization and this city consists of a floating population coming from many other provinces; thus, pursuing an epidemiological link is rather difficult. However, we have detailed information on the patients’ residential districts and we have added this information to the logistic regression analyses. Second, our samples were obtained in a population-based setting in an Asian city; but to generalize our finding, we analyzed another African cohort set available in the public by using the same methodology. Third, performing *in vitro* experiments to elucidate the functional significance of each genetic variant was beyond our scope owing to resource limitation. Nevertheless, the extracted genes were associated with the spread of INH-resistant strains carrying *katG*-S315T mutation and these reached statistically significant levels by using the bacterial GWAS approach based on LMM.

Previous studies suggest that the *katG* gene’s physiological function is not largely reduced by S315T substitution^63^. Its catalase-peroxidase-peroxynitritase activities may play a part to protect Mtb against reactive oxygen and nitrogen species derived from the phagocyte oxidative burst in human macrophages^63,64^. This may link KatG with other pathogen factors relevant to immune evasion or virulence such as PE/PPE^65,66^, although possible additive or synergistic effects on fitness should be further investigated. It is desirable to conduct validation studies in different populations. These Mtb genes are attractive candidates, presumably because of their relevance to the pathogen’s virulence, and they could be important sources to consider in *in vitro* and in animal models.

In conclusion, WGS data demonstrated the status of primary drug resistance at gene levels in the Hanoi city, and bacterial GWAS was performed to identify candidate genes that may facilitate the spreading of INH-resistant strains. Our findings provide new insights into the pathogenic mechanisms possibly mediated by the candidate genes including *PE/PPE*, by which drug-resistant Mtb can maintain epidemiological fitness and spread in high-burden countries such as in Asia and Africa.

## Methods

### Study sites, patient recruitment, and sample collection

This was a part of our cohort study of patients who were over 16 years of age and who were newly diagnosed with smear-positive pulmonary TB without any treatment history in Hanoi, Vietnam during 2007–2009, in which basic data with clinical interpretation were published in a previous report^12,67–69^. In brief, we included 7 of the 14 districts in Hanoi as the catchment area, where more than half of new smear-positive TB patients in the city were diagnosed and treated in the area during the study period^12^. Sputum specimens were collected before starting the treatment, 92.7% of which revealed culture-positive, and drug susceptibility testing for first-line drugs was performed using the WHO standard proportional method^12^. The patients’ clinico-epidemiological information was also collected. Written informed consent was obtained from all the patients.

### Ethics statement

This cohort study was approved by the Ethical Committees of the Ministry of Health, Vietnam, National Center for Global Health and Medicine, and the Research Institute of Tuberculosis, Japan Anti-Tuberculosis Association, Japan. All experiments were performed in accordance with relevant guidelines and regulations. In the case of minors, their parents provided written informed consent.

### WGS

Mycobacterial DNA samples from Löwenstein-Jensen culture media were extracted using the Isoplant kit (Nippon Gene, Tokyo, Japan) and analyzed using Illumina HiSeq and MiSeq systems (Illumina, San Diego, CA, USA). These experiments were performed using a class II safety cabinet in a biosafety level 3 laboratory to prevent contamination. For Hiseq. 2500, a library of WGS was prepared using an automated sample preparation system (Agilent Technologies Inc.) with the TruSeq DNA PCR free sample prep kit (Illumina). For Miseq, a library was prepared from 200 ng of genomic DNA with the TruSeq Nano DNA LT Sample Preparation Kit (Illumina), following the manufacturer’s instructions. Paired-end (2 × 150 bp) sequencing was performed using Hiseq. 2500. For Miseq, the paired-end (2 × 250 bp or 2 × 300 bp) sequencing system was used. The sequence data are available in the DDBJ/EMBL/GenBank databases under the accession numbers DRA008666-7 and DRA008677.

### Extracting single nucleotide variants (SNVs) for Mtb lineages/sublineages and genetic clustering

Briefly, after trimming and excluding severely contaminated samples, sequence reads were mapped to the H37Rv genome (AL123456.3) by using BWA-MEM 0.7.15 (https://github.com/lh3/bwa), followed by variant calling with the Genome analysis toolkit (GATK version 3.7)^70^. Only paired-end fastq files with average depth more than 25X were accepted for the analysis. The criteria set for identifying SNVs and small indels included Q30 minimum base call quality score and a minimum coverage depth of 10X. Drug resistance-conferring mutations, including small indels and lineage-specific variations, were extracted using the TB-Profiler version 0.3.7^9,22^. The Beijing genotype of lineage-2 (L2.2) was further classified into ancient and modern Beijing sublineages by detecting the SNVs at the nucleotide position 649,345, which is equivalent to the presence of IS6110 in the NTF region^71^.

Large deletions were screened throughout the mapped reads by seeking zero or low-depth areas (<15% of the average depth) using an in-house python script and then visualized for confirmation with the Integrative Genomics Viewer (IGV) version 2.3.91. For this deletion screening, complete genome sequences of the clinical isolates in our Hanoi cohort, AP018033 to AP018036^72,73^ as well as H37Rv genome, were used as reference sequences. After excluding ambiguous variants in categories of repetitive and insertion sequences and phages, genetic clusters were defined by the pairwise differences of no more than five SNVs^74^. A phylogenetic tree was constructed by the maximum likelihood method using RAxML version 8.2.8^75^ and then visualized with plotTree (https://github.com/katholt/plotTree) by using a lineage-7 strain ERR181435 as an out-group.

### Analyses of bacterial GWAS

The associations between the phenotypes and the presence or absence of 31-bp short sequences, k-mers, in the genome were investigated using a genome-wide efficient mixed model association algorithm, the GEMMA software (https://github.com/genetics-statistics/GEMMA). At first, the DSK software (https://github.com/GATB/dsk) was used for listing all the unique 31-bp DNA k-mers, and then their presence or absence in all the samples was analyzed as mentioned above^20^.

DBGWAS, an extended k-mer-based GWAS tool with compacted De Bruijn graph^76^, was further used to confirm the genetic variants associated with the phenotypes of interest. Sequence reads were assembled using SPAdes v3.13.0^77^ and Platanus 1.2.4^78^ when appropriate, and the generated contigs were used for BLAST search (ncbi-blast-2.8.1+) to identify the location of the phenotype-associated k-mers. Bonferroni correction was applied for multiple testing; the threshold of the significance after correction was calculated as 0.05 divided by the number of variants identified in the study samples.

To investigate whether any variant, including SNVs or indels in the whole genome, has any possible association with the phenotypes of interest, we used bugwas R package^20^ with built-in GEMMA. The bi-allelic SNVs were used to calculate the relatedness matrix of the samples for LMMs to control for the population structure. Likelihood ratio tests were used for assessing the significant associations.

Phylogenetic convergence tests (phyOverlap)^26^ were also performed to identify the convergent variants associated with the phenotypes. Herein, Benjamini-Hochberg adjustment at 0.05 false discovery rate level was applied as the q value for phyOverlap.

To compare the findings obtained from the GWAS analysis of our 332 samples in Vietnam, another set of WGS data from 337 clinical isolates, which were collected in the KwaZulu-Natal province of South Africa from 2008 to 2013 in a study conducted by Cohen *et al*.^19^ to investigate the emergence of drug-resistant TB (hereafter referred to as KwaZulu-Natal study), were retrieved from the public database, and analyzed in a similar way.

### Other statistical analyses

Chi square and Fisher’s exact tests were performed to compare the differences in the proportions among the groups. Venn diagram (R version 3.4.4 VennDiagram package) was used to demonstrate the common gene(s) harboring variants, including k-mers, associated with different phenotypes. Possible associations between the given genetic variations and INH-resistant clusters, adjusted for Mtb lineages and patients’ age, gender, and living area were further studied using logistic regression models. These analyses were performed using STATA version 12 (StataCorp, College Station, TX, USA), and P values less than 0.05 were considered statistically significant.

## Supplementary information

Supplementary information

## Data Availability

All data pertaining to the manuscript have been provided in the forms of tables and figures. Supporting Information is available as Supplementary Tables S1–S6 and Figs S1–S7. Datasets pertaining to the sequence searches described here are available from the corresponding author on request.
