# Supplementary material for: Whole genome sequencing, analyses of drug resistance-conferring mutations, and correlation with transmission of Mycobacterium tuberculosis carrying katG-S315T in Hanoi, Vietnam
Source: Sci Rep. 2019 Oct 25;9:15354. doi: 10.1038/s41598-019-51812-7 (PMC6814805; doi:10.1038/s41598-019-51812-7)
Supplement: Supplementary file 1 — Supplementary information [file 41598_2019_51812_MOESM1_ESM.pdf]

Supplementary information

Whole genome sequencing, analyses of drug resistance-conferring mutations, and correlation with transmission of *Mycobacterium tuberculosis* carrying *katG*-S315T in Hanoi, Vietnam

Nguyen Thi Le Hang[1*], Minako Hijikata[2*], Shinji Maeda[3], Pham Huu Thuong[4], Jun Ohashi[5], Hoang Van Huan[4], Nguyen Phuong Hoang[6], Akiko Miyabayashi[2], Vu Cao Cuong[7], Shintaro Seto[2], Nguyen Van Hung[8], Naoto Keicho[9,10]

[1]NCGM-BMH Medical Collaboration Center, Hanoi, Vietnam.
[2]Department of Pathophysiology and Host Defense, The Research Institute of Tuberculosis, JATA, Tokyo, Japan.
[3]Faculty of Pharmaceutical Sciences, Hokkaido University of Science, Hokkaido, Japan.
[4]Hanoi Lung Hospital, Hanoi, Vietnam.
[5]Department of Biological Sciences, Graduate School of Science, The University of Tokyo, Tokyo, Japan.
[6]Department of Microbiology, Hanoi Lung Hospital, Hanoi, Vietnam.
[7]Hanoi Department of Health, Hanoi, Vietnam.
[8]Department of Microbiology, National Lung Hospital, Hanoi, Vietnam.
[9]The Research Institute of Tuberculosis JATA, Tokyo, Japan.
[10]National Center for Global Health and Medicine, Tokyo, Japan.

*These authors contributed equally to this work.
Correspondence and request for materials should be addressed to N.K.
(nkeicho-tky@umin.ac.jp)

Corresponding author
Naoto Keicho, MD, PhD
Vice Director
The Research Institute of Tuberculosis
Japan Anti-Tuberculosis Association
3-1-24 Matsuyama, Kiyose, Tokyo 204-8533, JAPAN
E-mail: nkeicho-tky@umin.ac.jp

Supplementary Fig. S1: Deletions identified in drug resistance-related genes observed in Hanoi samples

353-bp deletion in *pncA* (S1a), 6-bp deletion in *rpoB* (S1b), 1-bp deletion in *gid* (S1c), and 1-bp deletion in *ethA* (S1d) in Hanoi's 332 samples

S1a)

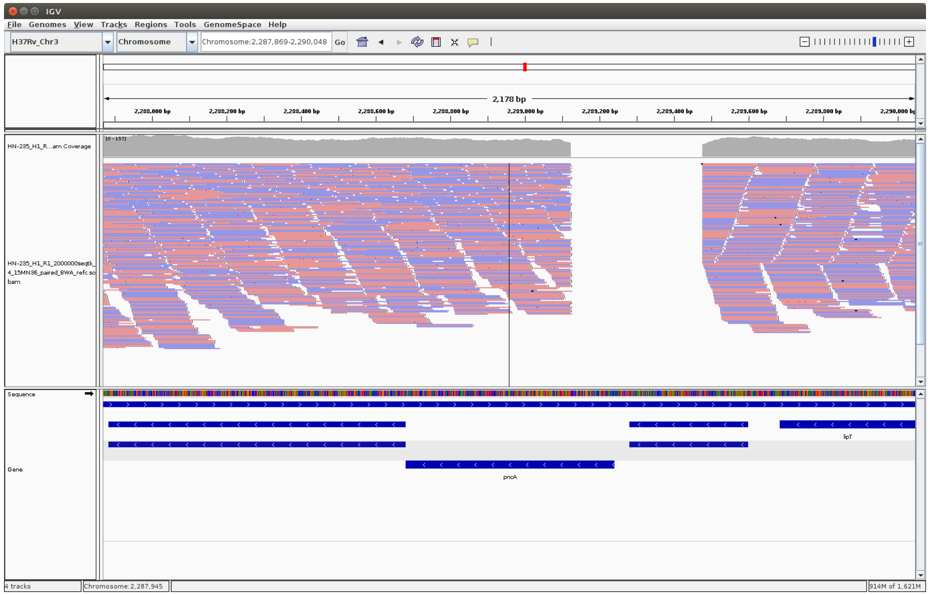

S1b)

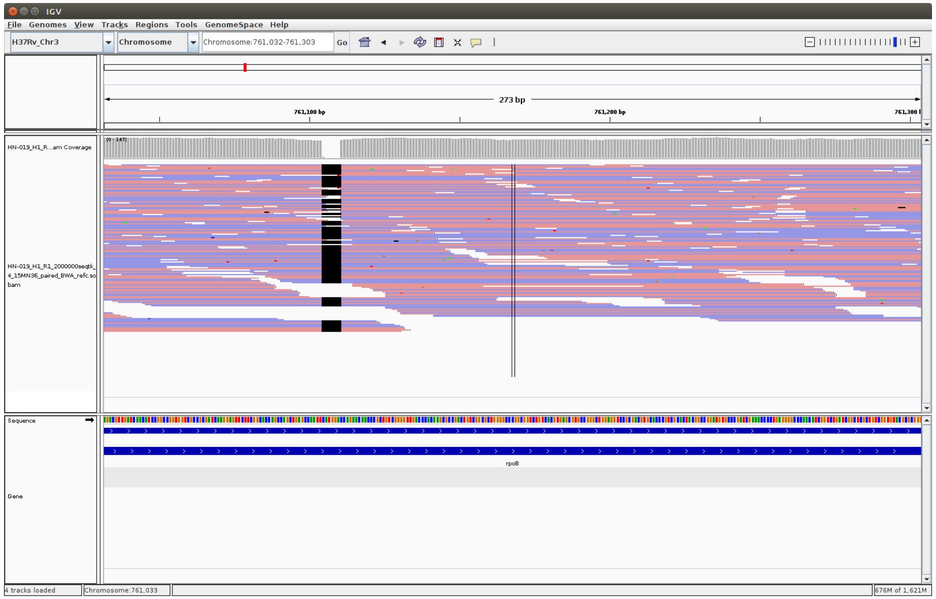

S1c)

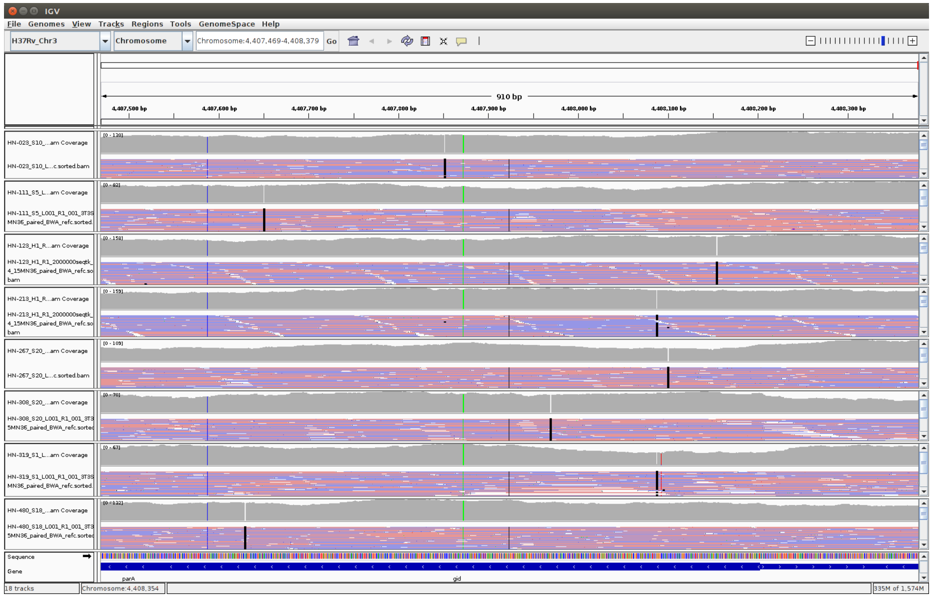

S1d)

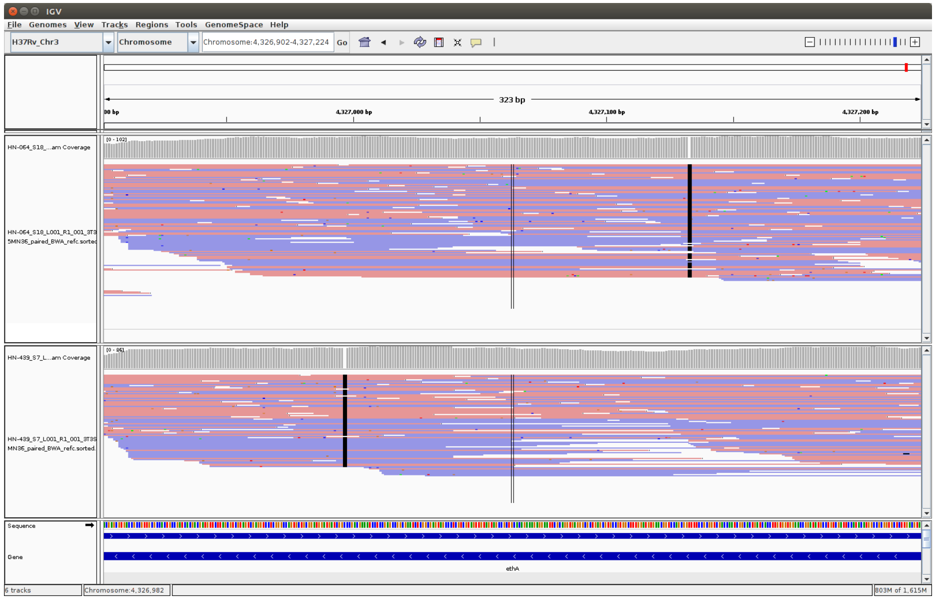

Supplementary Fig S2: a) k-mers in *PPE18* and *PPE19* significantly associated with clustered strains carrying *katG*-S315T among Hanoi and KwaZulu-Natal study population, viewed using IGV

PPE18

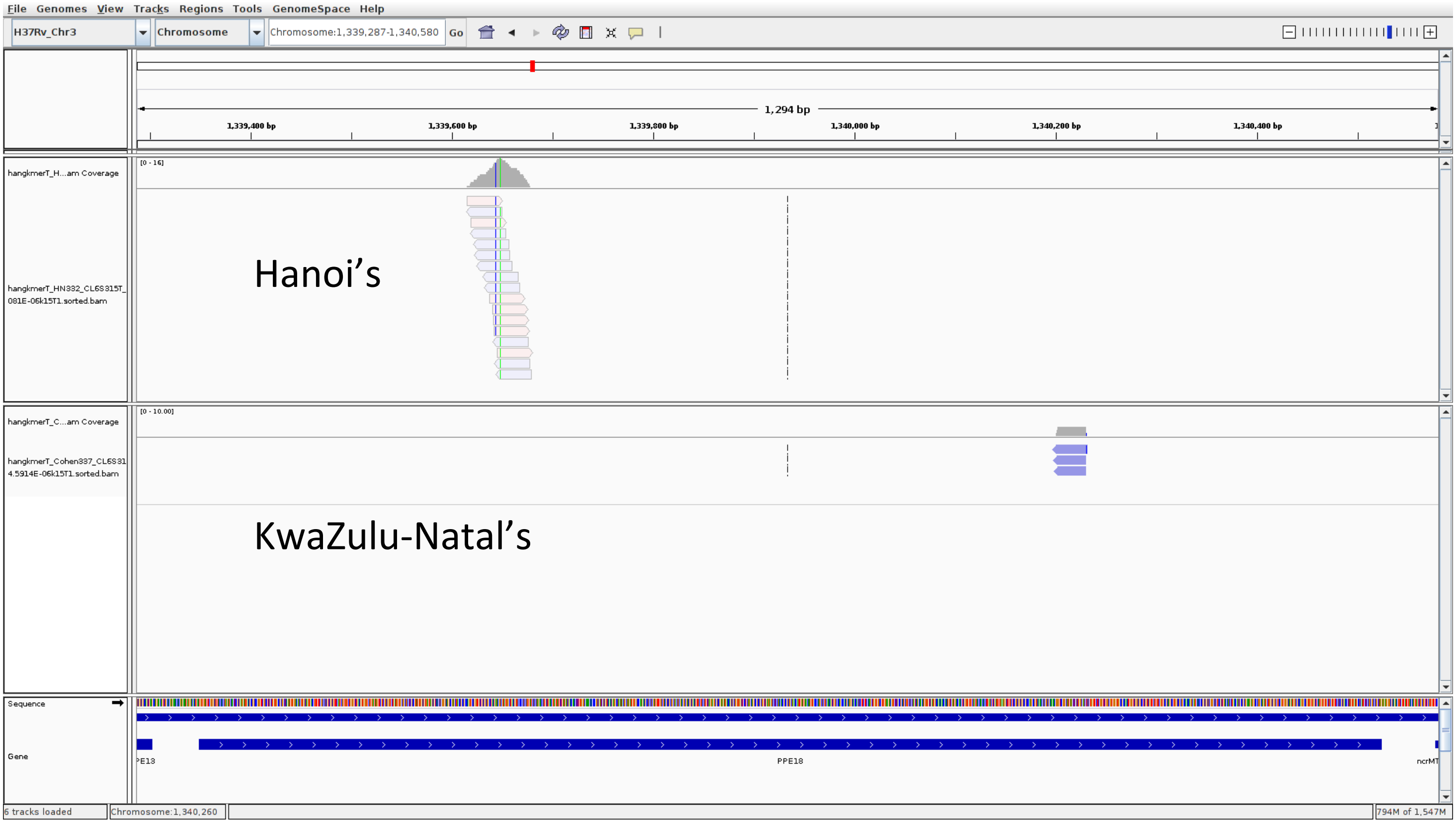

PPE19

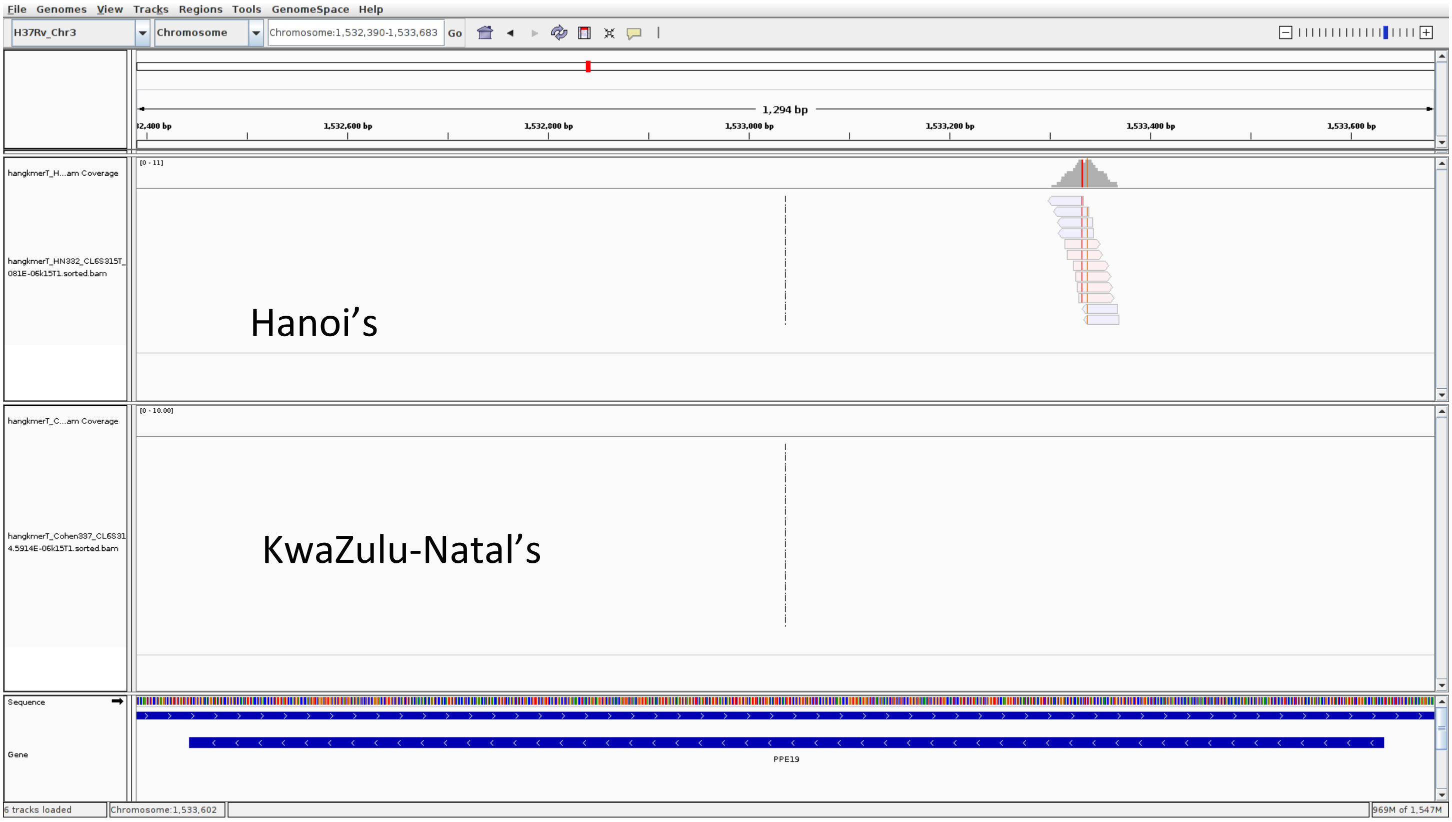

Supplementary Fig S2: b) k-mers in *gid* and *emrB* significantly associated with clustered strains carrying *katG*-S315T among Hanoi and KwaZulu-Natal study population, viewed using IGV

gid

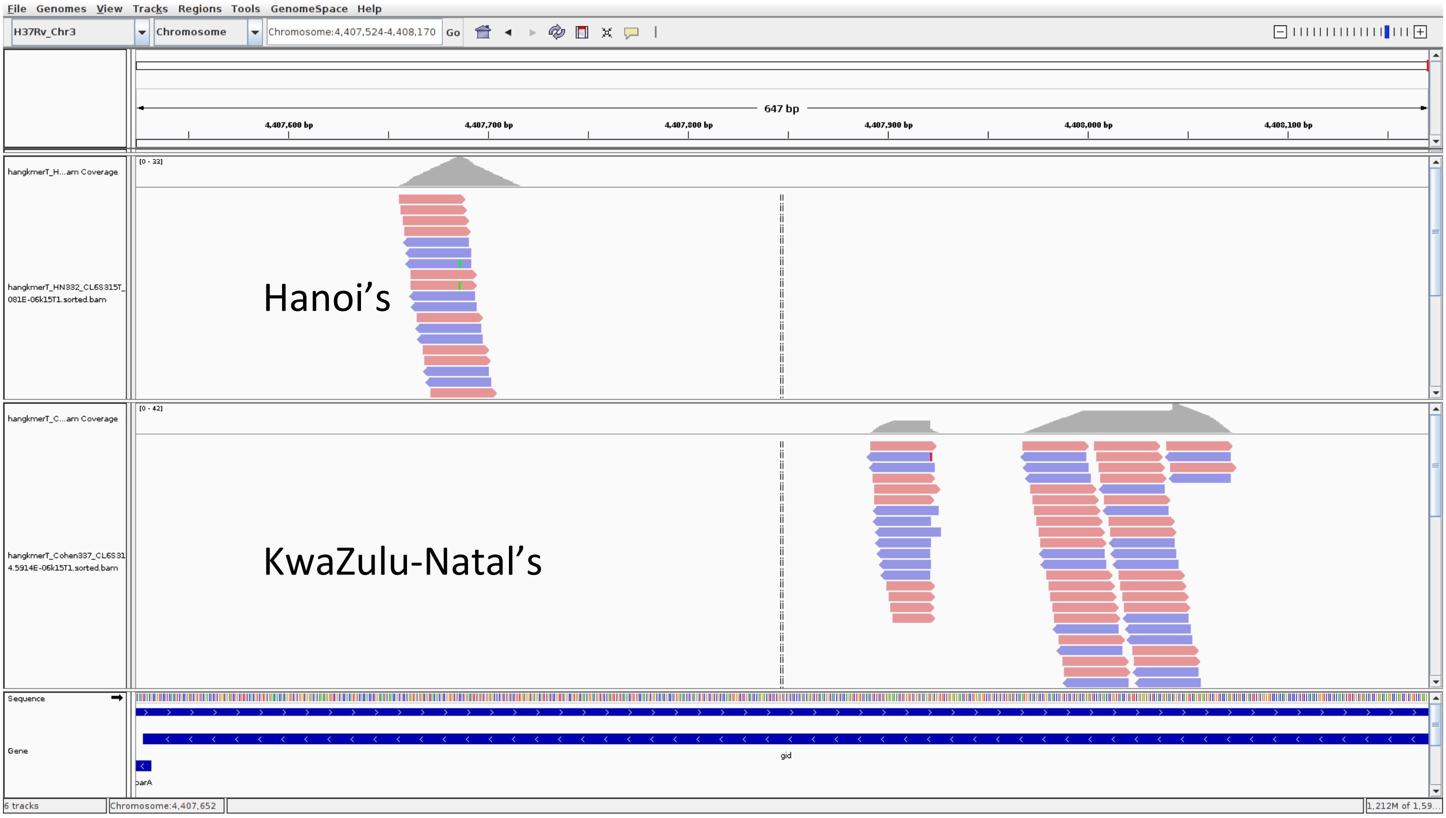

emrB

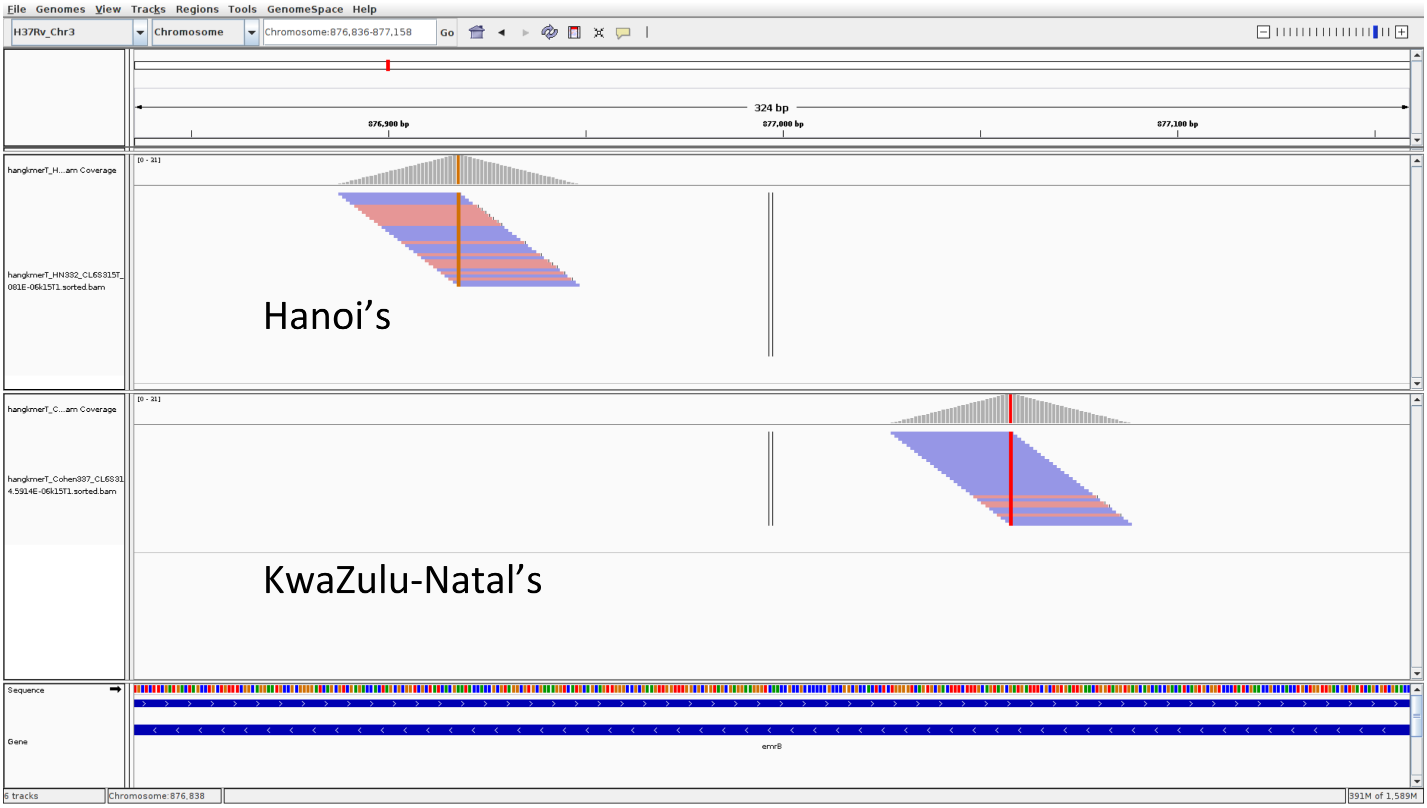

Supplementary Fig S2: c) k-mers in *Rv1588c* and *pncA* significantly associated with clustered strains carrying *katG*-S315T among Hanoi and KwaZulu-Natal study population, viewed using IGV

Rv1588c

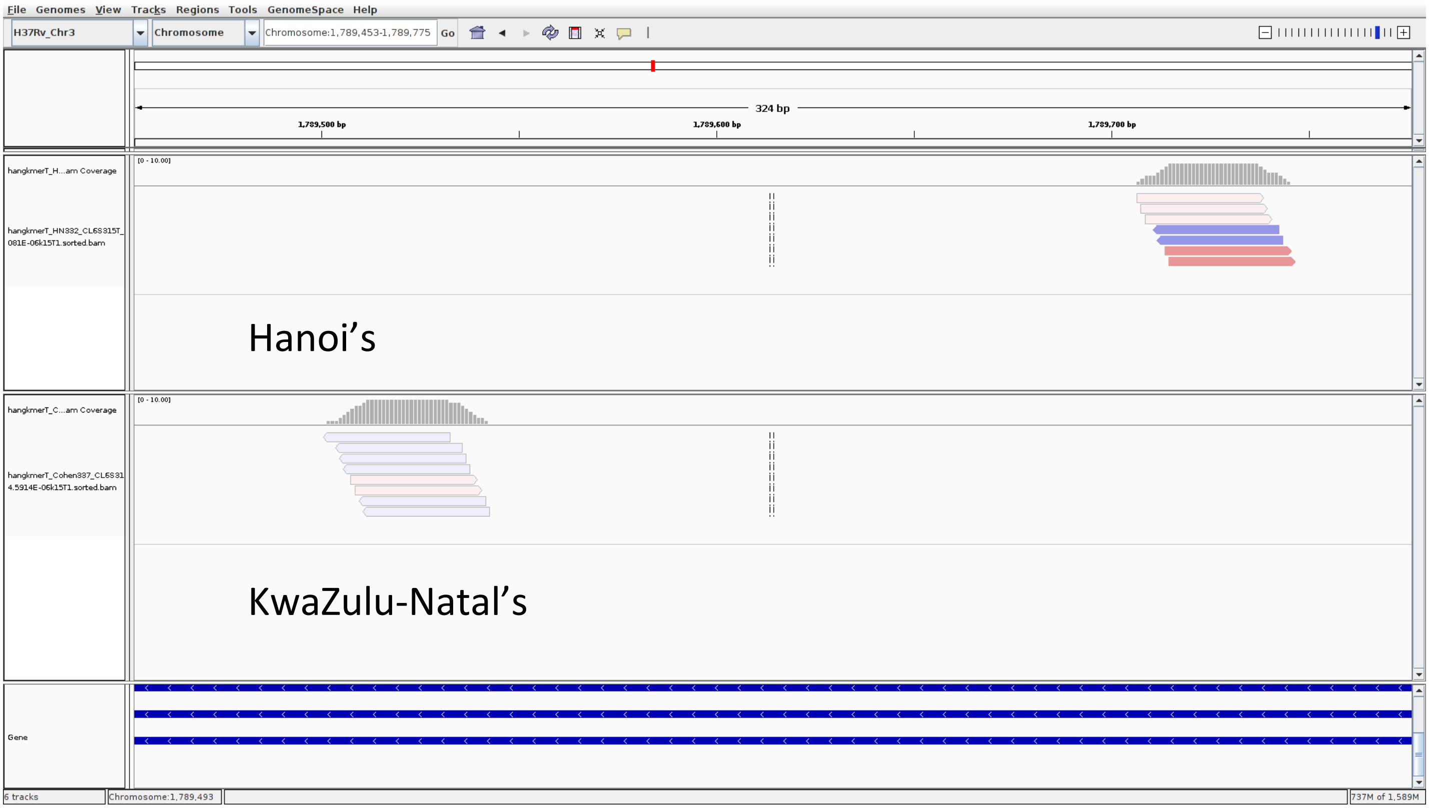

pncA

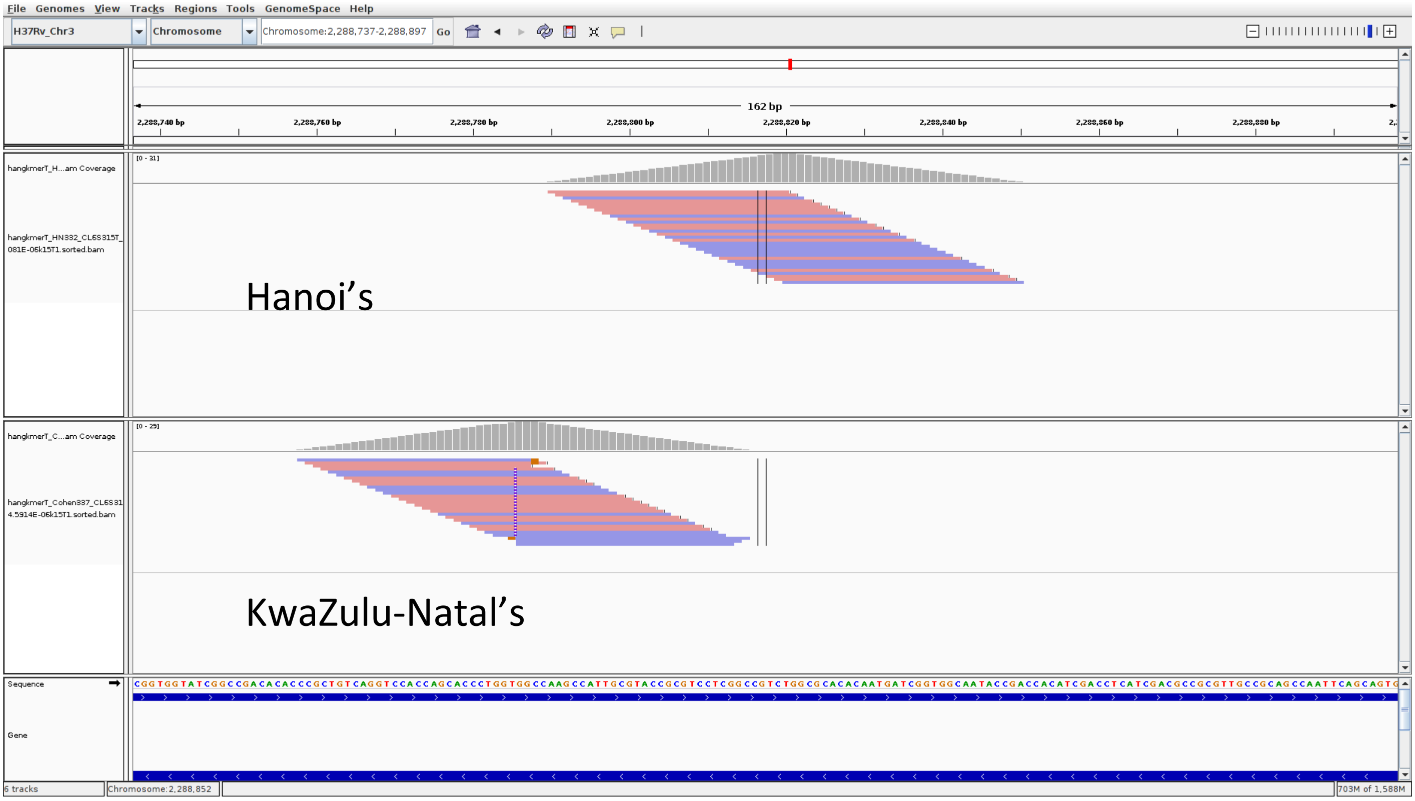

Supplementary Fig. S3: Unitigs found by k-mer-based DBGWAS and their significant associations with cluster[+]/*katG*-S315T[+] (phenotype [+]) among Hanoi's 332 samples

Colors are continuously interpolated between blue for phenotype [-] and red for phenotype [+]. The size of the nodes corresponds to allele frequency. Nodes circled in black are significant unitigs: One significant unitig in *PPE18* (S3a), three in *gid* (S3b), two in *emrB* (S3c), and two in *Rv1588c* (S3d) are shown. No significant unitigs were identified in *pncA*.

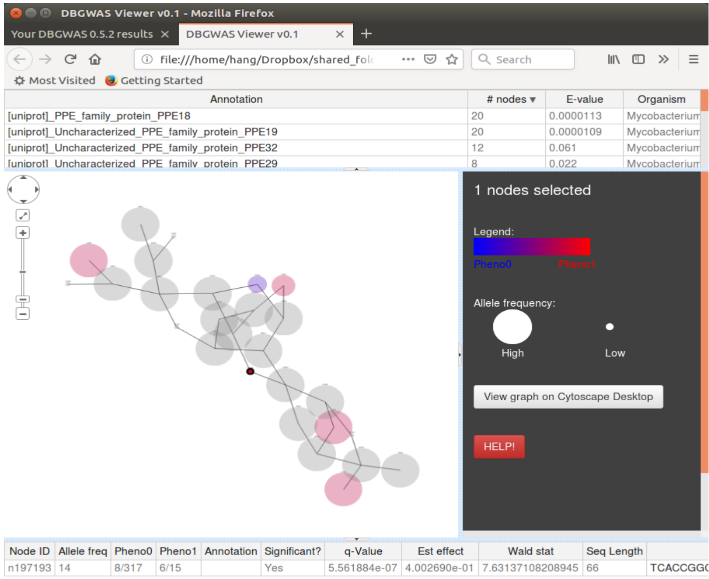

S3a) *PPE18*

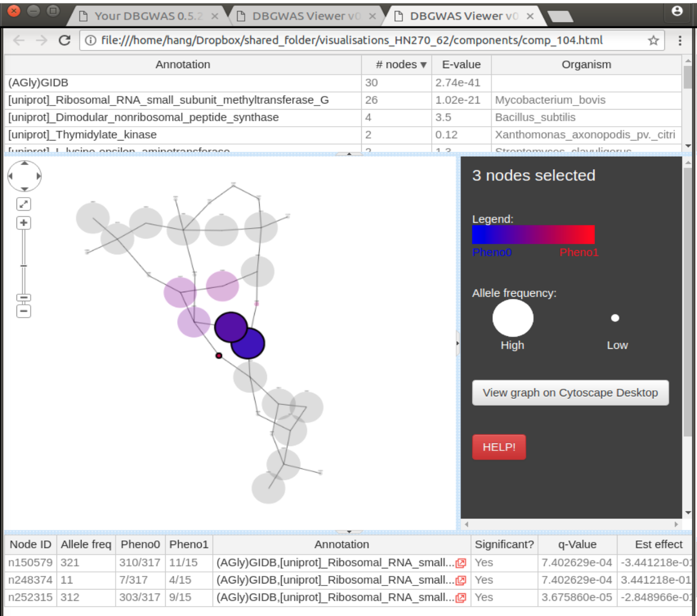

S3b) *gid*

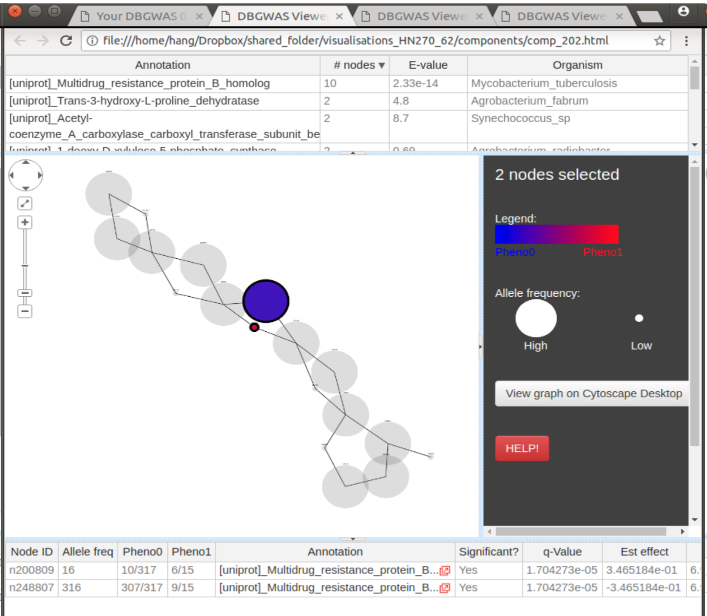

S3c) *emrB*

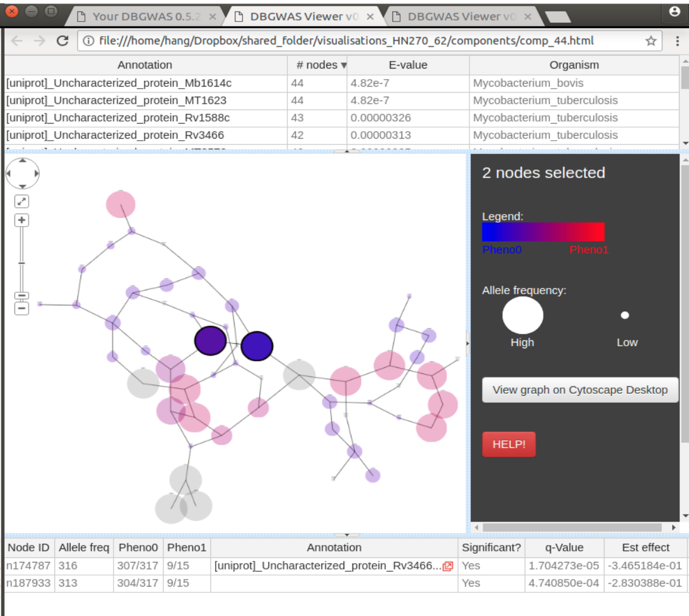

S3d) *Rv1588c*

Supplementary Fig. S4: Sequence similarity observed in *PPE18*, *PPE19* and *PPE60* of H37Rv genome

Nucleotide sequences of *PPE18*, *PPE19* and *PPE60* in the H37Rv genome (AL123456.3) were aligned. Dots indicate nucleotides identical to those shown in the top (*PPE18*), and dashes indicate gaps. When two sequences among the three have the same nucleotide, the regions were shown in a dark blue background. Upper and lower red boxes indicate the *PPE18/19* regions to which significant k-mers in Hanoi and KwaZulu-Natal samples were mapped respectively.

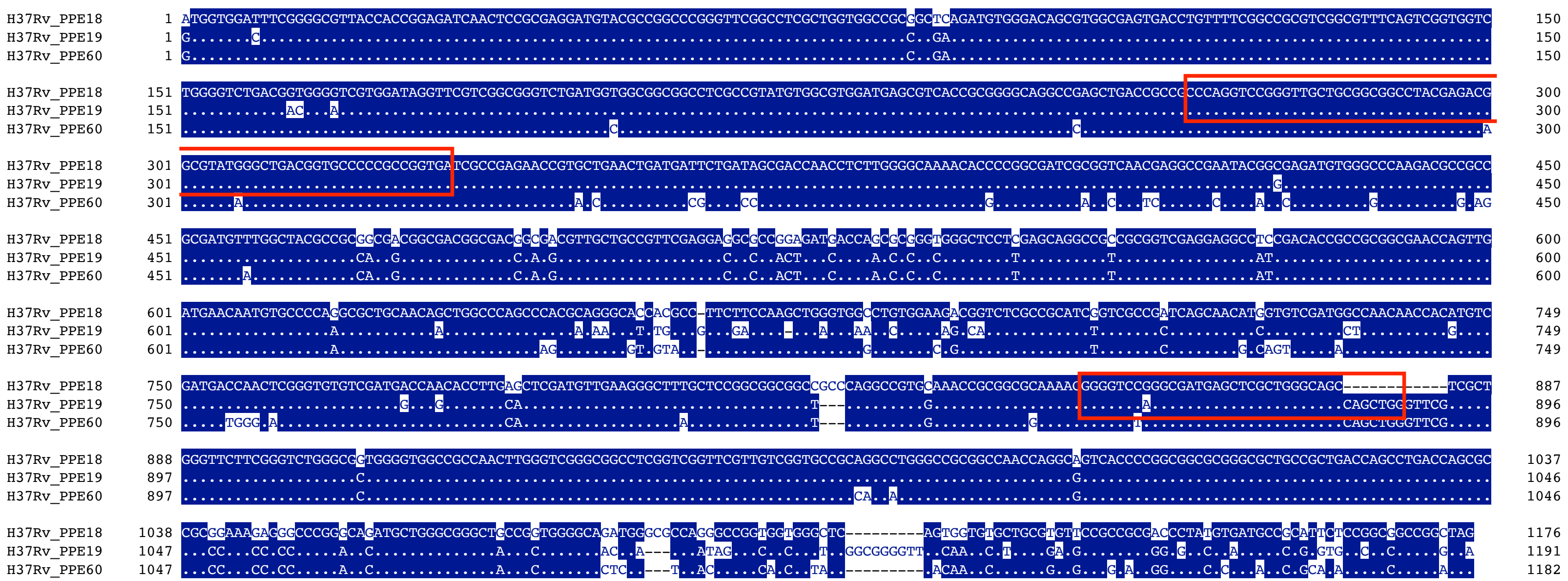

Supplementary Fig. S5: GWAS was performed to investigate the association between SNVs and cluster[+]/*katG*-S315T[+] among Hanoi samples, controlling for population structure

S5a) Bayesian Wald test of significance of lineage-specific association with cluster[+]/*katG*-S315T[+]; S5b) Principal components correspond to lineages associated with cluster[+]/*katG*-S315T[+]; S5c) Manhattan plot showing significance of unique variants after controlling for population structure, with variants clustered by principal component.

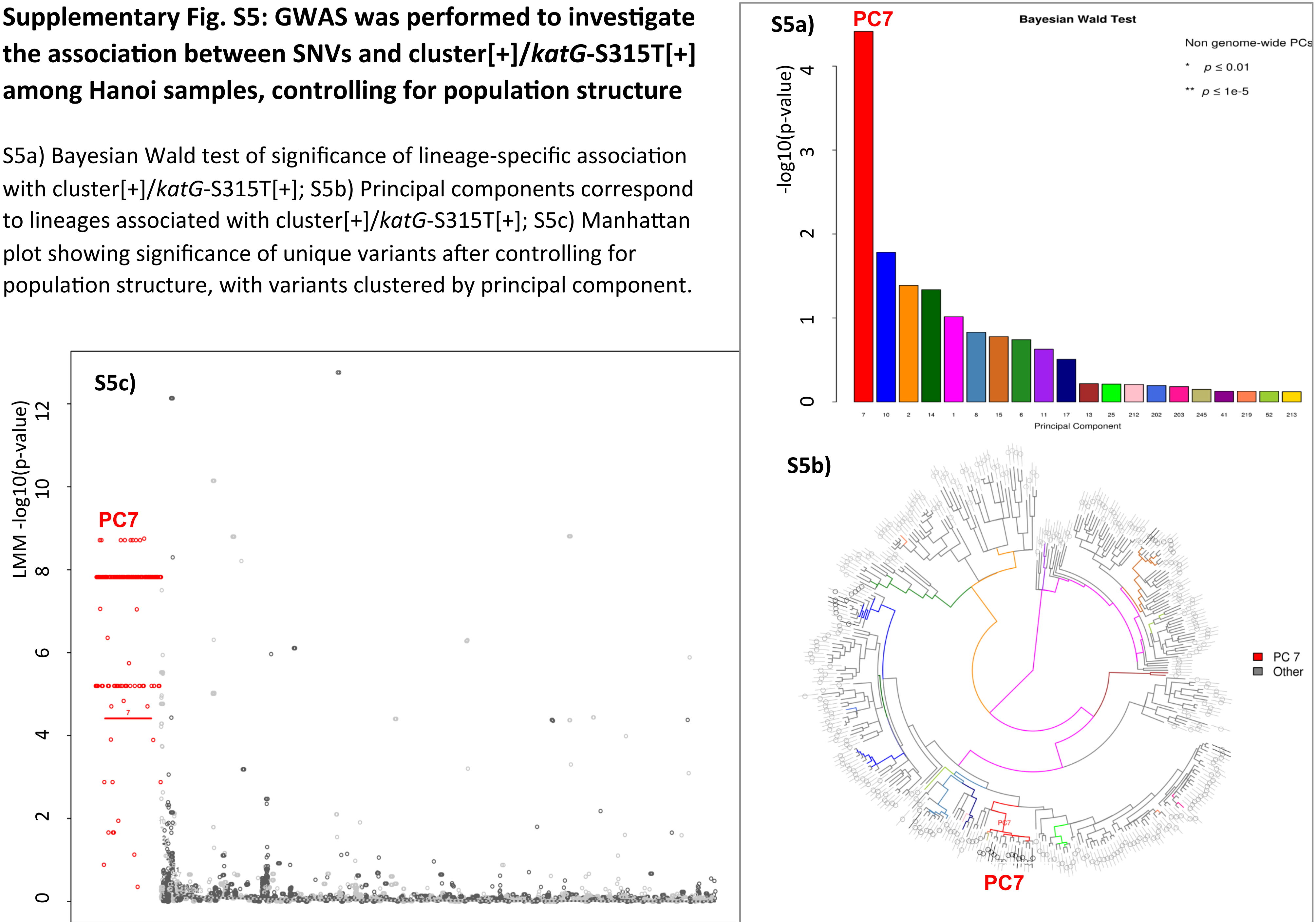

Supplementary Fig S6: Genomic structure of the *PPE46* to *PPE47* region

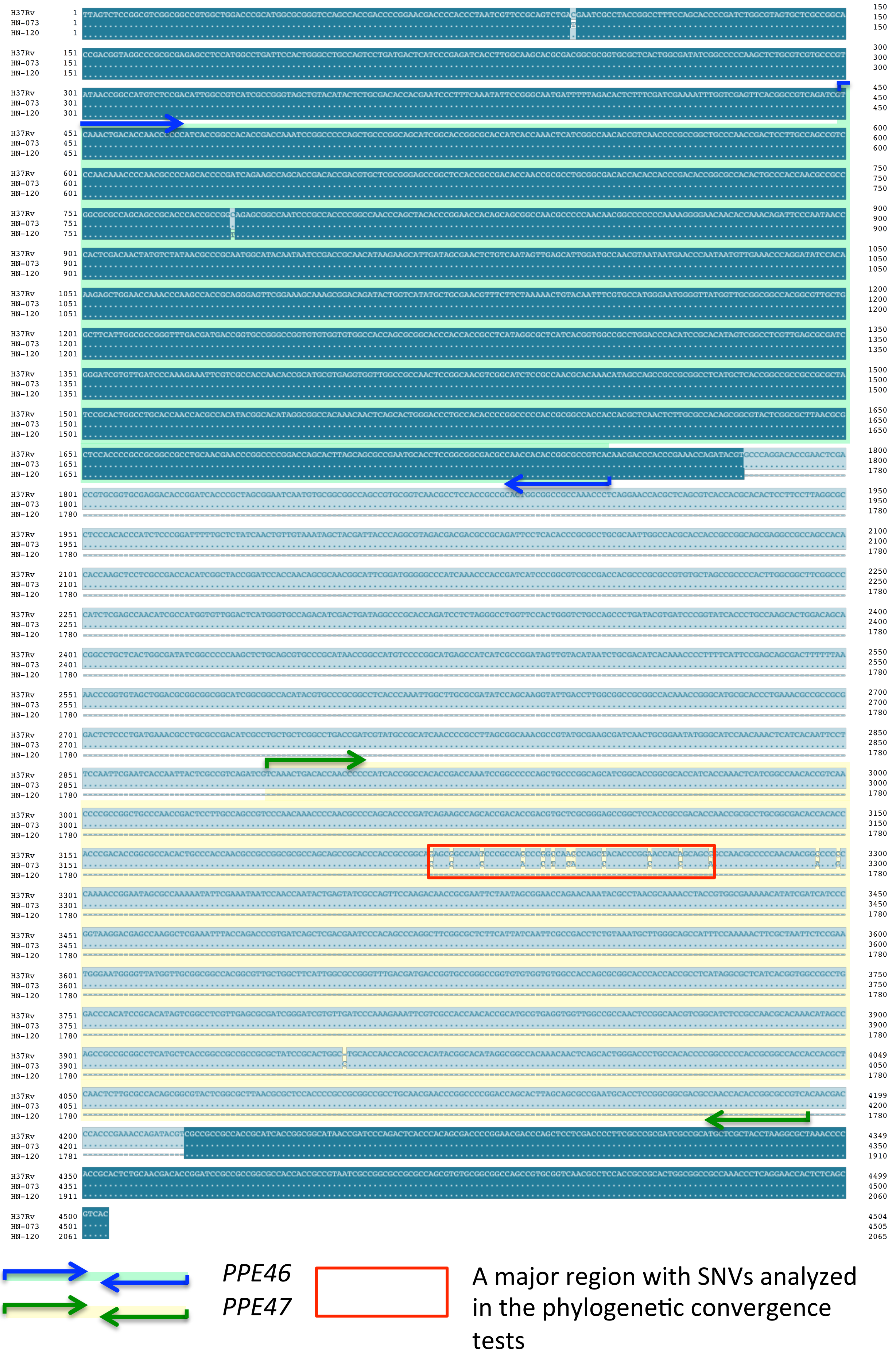

In two Hanoi strains (HN-073 and HN-120), nucleotide sequences spanning *PPE46* and *PPE47* were aligned with a reference strain H37Rv at positions 3376490 to 3380993 of AL123456.3. The *PPE46* region is overlaid in green, and the *PPE47* region in yellow. When three sequences show identical nucleotides, they are shown in white letters in a dark blue background. When two sequences among the three are identical, they are shown in a faint blue background. Dotted lines indicate the nucleotides identical to those of H37Rv (AL123456.3). A dashed line indicates a deleted region observed in HN-120.

The SNVs in *PPE47* were associated with the phenotype through phyOverlap analysis, and many of them were located in the region at positions from 3379708 to 3379763 of H37Rv (AL123456.3) shown in the red box in *PPE47*. These SNVs were identified in most of the Hanoi strains, including HN-073, irrespective of their lineages (L1, L2 or L4).

However, in 35 Hanoi strains, including HN-120, *de novo* assembled sequences revealed the presence of a large deletion of 2,439 bp around the region. Therefore, the above SNVs were not detected. Such a deletion resulted in in-frame connection of *PPE46* and *PPE47* ORFs, and thus generated a novel chimeric PPE gene. The other rare deletions (two cases) are not presented here. The schematic diagrams are also shown in supplementary Fig. S7.

Supplementary Fig. S7: Scheme of *PPE46/47* chimeric genes observed in Hanoi sample set

S7a) Structure and sequence similarity of *PPE46/47*

S7b) Three deletion patterns around *PPE46/47* and generation of chimeric genes

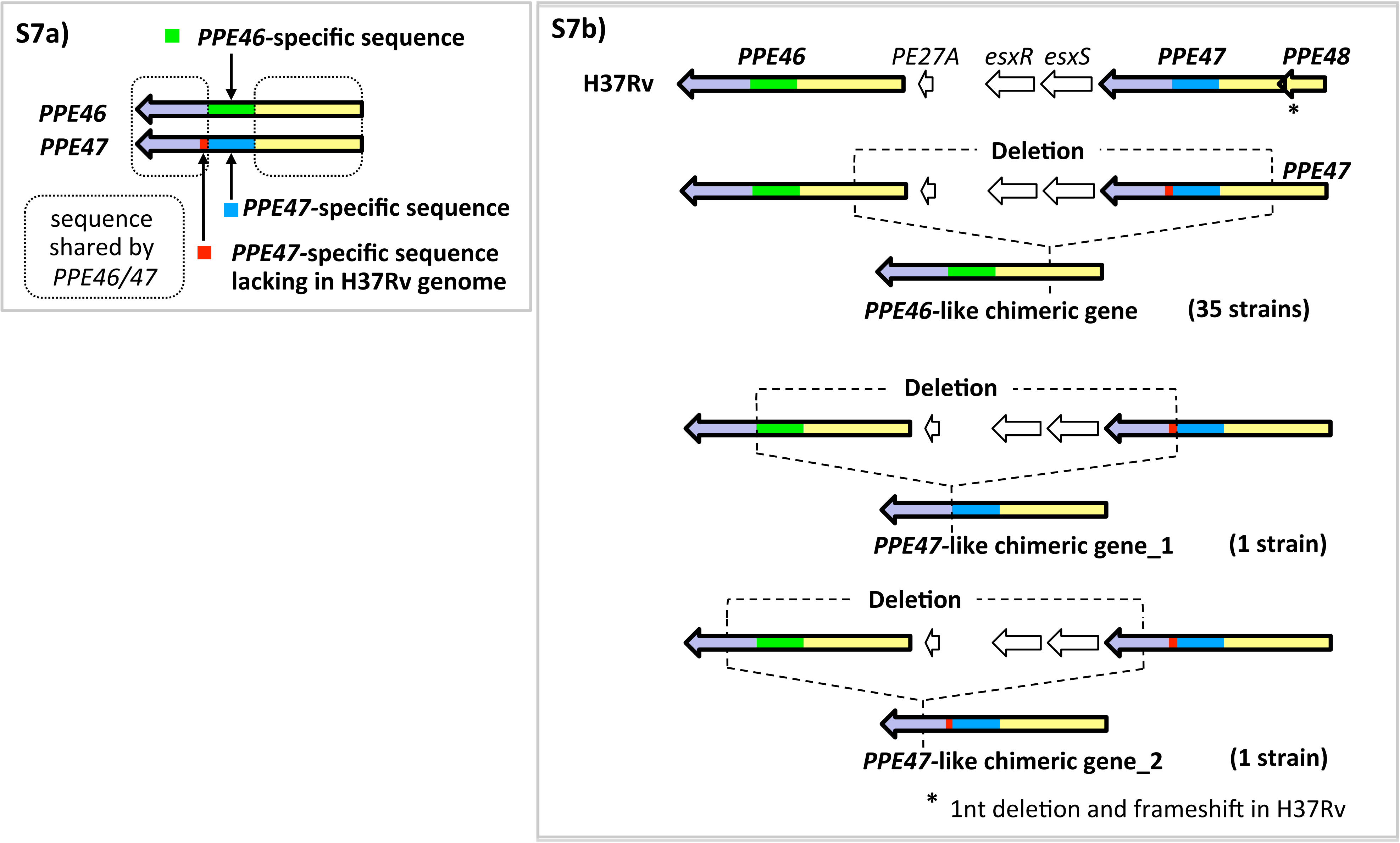

Supplementary Table S1: Five common genes (*PPE18/PPE19, gid, emrB, pncA*, and *Rv1588c*) and their k-mers that were significantly associated with the clustered strains carrying the *katG*-S315T mutation of Hanoi's 332 and KwaZulu-Natal's 337 samples

rs	Kmer	Kmer_sequence	Presence of k-mers among phenotype[-]	Presence of k-mers among phenotype[+]	Start position	End position	Gene	P value (Wald test)	P value (Likelihood ratio test)
**Hanoi's study population**									
rs14	kmer5300519	ACCGGCGGGGGCACCGTCAGCCCATACGTCG	9	6	1339677	1339647	*PPE18*	2.60279E-12	1.840E-09
rs14	kmer16678658	CATACGTCGTCGCGTAGGCCGCCGCAGCAAC	9	6	1339655	1339625	*PPE18*	2.60279E-12	1.840E-09
rs14	kmer9905015	AGGTCCGGGTTGCTGCGGCGGCCTACGCGAC	9	6	1339617	1339647	*PPE18*	2.60279E-12	1.840E-09
rs14	kmer6190163	ACGCGACGACGTATGGGCTGACGGTGCCCCC	9	6	1339641	1339671	*PPE18*	2.60279E-12	1.840E-09
rs14	kmer18366644	CCCATACGTCGTCGCGTAGGCCGCCGCAGCA	9	6	1339657	1339627	*PPE18*	2.60279E-12	1.840E-09
rs14	kmer15960566	CAGCCCATACGTCGTCGCGTAGGCCGCCGCA	9	6	1339660	1339630	*PPE18*	2.60279E-12	1.840E-09
rs14	kmer21866099	CGACGACGTATGGGCTGACGGTGCCCCCGCC	9	6	1339645	1339674	*PPE18*	2.60279E-12	1.840E-09
rs14	kmer6778669	ACGTCGTCGCGTAGGCCGCCGCAGCAACCCG	9	6	1339652	1339622	*PPE18*	2.60279E-12	1.840E-09
rs14	kmer32249733	TACGCGACGACGTATGGGCTGACGGTGCCCC	9	6	1339640	1339670	*PPE18*	2.60279E-12	1.840E-09
rs14	kmer30472299	GCGACGACGTATGGGCTGACGGTGCCCCCGC	9	6	1339645	1339673	*PPE18*	2.60279E-12	1.840E-09
rs14	kmer15135322	CACCGGCGGGGGCACCGTCAGCCCATACGTC	9	6	1339678	1339650	*PPE18*	2.60279E-12	1.840E-09
rs14	kmer25283133	CGTCAGCCCATACGTCGTCGCGTAGGCCGCC	9	6	1339663	1339633	*PPE18*	2.60279E-12	1.840E-09
rs14	kmer10848459	ATACGTCGTCGCGTAGGCCGCCGCAGCAACC	9	6	1339654	1339624	*PPE18*	2.60279E-12	1.840E-09
rs14	kmer20760958	CCTACGCGACGACGTATGGGCTGACGGTGCC	9	6	1339638	1339668	*PPE18*	2.60279E-12	1.840E-09
rs14	kmer33799972	TCCGGGTTGCTGCGGCGGCCTACGCGACGAC	9	6	1339620	1339648	*PPE18*	2.60279E-12	1.840E-09
rs14	kmer29824538	GCACCGTCAGCCCATACGTCGTCGCGTAGGC	9	6	1339667	1339637	*PPE18*	2.60279E-12	1.840E-09
rs14	kmer24545936	CGGCGGGGGCACCGTCAGCCCATACGTCGTC	9	6	1339675	1339645	*PPE18*	2.60279E-12	1.840E-09
rs14	kmer20529728	CCGTCAGCCCATACGTCGTCGCGTAGGCCGC	9	6	1339664	1339634	*PPE18*	2.60279E-12	1.840E-09
rs14	kmer5379821	ACCGTCAGCCCATACGTCGTCGCGTAGGCCG	9	6	1339665	1339635	*PPE18*	2.60279E-12	1.840E-09
rs14	kmer20398506	CCGGGTTGCTGCGGCGGCCTACGCGACGACG	9	6	1339621	1339648	*PPE18*	2.60279E-12	1.840E-09
rs14	kmer30113731	GCCCATACGTCGTCGCGTAGGCCGCCGCAGC	9	6	1339658	1339628	*PPE18*	2.60279E-12	1.840E-09
rs14	kmer33207065	TCAGCCCATACGTCGTCGCGTAGGCCGCCGC	9	6	1339661	1339631	*PPE18*	2.60279E-12	1.840E-09
rs14	kmer18352863	CCCAGGTCCGGGTTGCTGCGGCGGCCTACGC	9	6	1339614	1339643	*PPE18*	2.60279E-12	1.840E-09
rs14	kmer6703764	ACGTATGGGCTGACGGTGCCCCCGCCGGTGA	9	6	1339650	1339679	*PPE18*	2.60279E-12	1.840E-09
rs14	kmer23336712	CGCGACGACGTATGGGCTGACGGTGCCCCCG	9	6	1339642	1339672	*PPE18*	2.60279E-12	1.840E-09
rs14	kmer24517828	CGGCGGCCTACGCGACGACGTATGGGCTGAC	9	6	1339632	1339662	*PPE18*	2.60279E-12	1.840E-09
rs14	kmer32288280	TACGTCGTCGCGTAGGCCGCCGCAGCAACCC	9	6	1339653	1339623	*PPE18*	2.60279E-12	1.840E-09
rs14	kmer25463038	CGTCGCGTAGGCCGCCGCAGCAACCCGGACC	9	6	1339648	1339618	*PPE18*	2.60279E-12	1.840E-09
rs14	kmer15159943	CACCGTCAGCCCATACGTCGTCGCGTAGGCC	9	6	1339666	1339636	*PPE18*	2.60279E-12	1.840E-09
rs14	kmer5835122	ACGACGTATGGGCTGACGGTGCCCCCGCCGG	9	6	1339646	1339676	*PPE18*	2.60279E-12	1.840E-09
rs14	kmer26030968	CTACGCGACGACGTATGGGCTGACGGTGCCC	9	6	1339639	1339669	*PPE18*	2.60279E-12	1.840E-09
rs14	kmer8483714	AGCCCATACGTCGTCGCGTAGGCCGCCGCAG	9	6	1339659	1339629	*PPE18*	2.60279E-12	1.840E-09
rs14	kmer16444207	CAGGTCCGGGTTGCTGCGGCGGCCTACGCGA	9	6	1339616	1339643	*PPE18*	2.60279E-12	1.840E-09
rs14	kmer18004377	CCAGGTCCGGGTTGCTGCGGCGGCCTACGCG	9	6	1339615	1339643	*PPE18*	2.60279E-12	1.840E-09
rs14	kmer34614774	TCGTCGCGTAGGCCGCCGCAGCAACCCGGAC	9	6	1339648	1339619	*PPE18*	2.60279E-12	1.840E-09
rs14	kmer18070492	CCATACGTCGTCGCGTAGGCCGCCGCAGCAA	9	6	1339656	1339626	*PPE18*	2.60279E-12	1.840E-09
rs138	kmer22946493	CGCCCGATGCGATCATCACACGCCGGTGCTC	303	9	4407656	4407686	*gid*	2.6576E-08	5.21335E-08
rs229	kmer677956	AAAGGCGAGCGGGCTCACGACGAAGTACGGG	310	11	4407716	4407686	*gid*	1.6524E-06	2.31776E-06
rs229	kmer2514548	AAGGCGAGCGGGCTCACGACGAAGTACGGGA	310	11	4407715	4407685	*gid*	1.6524E-06	2.31776E-06
rs229	kmer2663726	AAGTACGGGAGCACCGGCGTGTGATGATCGC	310	11	4407694	4407664	*gid*	1.6524E-06	2.31776E-06

rs229	kmer4088267	ACACGCCGGTGCTCCCGTACTTCGTCGTGAG	310	11	4407673	4407703	*gid*	1.6524E-06	2.31776E-06
rs229	kmer5725621	ACGAAGTACGGGAGCACCGGCGTGTGATGAT	310	11	4407697	4407667	*gid*	1.6524E-06	2.31776E-06
rs229	kmer5791837	ACGACGAAGTACGGGAGCACCGGCGTGTGAT	310	11	4407700	4407670	*gid*	1.6524E-06	2.31776E-06
rs229	kmer6155368	ACGCCGGTGCTCCCGTACTTCGTCGTGAGCC	310	11	4407675	4407705	*gid*	1.6524E-06	2.31776E-06
rs229	kmer6542701	ACGGGAGCACCGGCGTGTGATGATCGCATCG	310	11	4407690	4407660	*gid*	1.6524E-06	2.31776E-06
rs229	kmer8914725	AGCGGGCTCACGACGAAGTACGGGAGCACCG	310	11	4407709	4407679	*gid*	1.6524E-06	2.31776E-06
rs229	kmer9582846	AGGCGAGCGGGCTCACGACGAAGTACGGGAG	310	11	4407714	4407684	*gid*	1.6524E-06	2.31776E-06
rs229	kmer10092635	AGTACGGGAGCACCGGCGTGTGATGATCGCA	310	11	4407693	4407663	*gid*	1.6524E-06	2.31776E-06
rs229	kmer12721235	ATGCGATCATCACACGCCGGTGCTCCCGTAC	310	11	4407662	4407692	*gid*	1.6524E-06	2.31776E-06
rs229	kmer14809154	CACACGCCGGTGCTCCCGTACTTCGTCGTGA	310	11	4407672	4407702	*gid*	1.6524E-06	2.31776E-06
rs229	kmer15271688	CACGACGAAGTACGGGAGCACCGGCGTGTGA	310	11	4407701	4407671	*gid*	1.6524E-06	2.31776E-06
rs229	kmer15362897	CACGCCGGTGCTCCCGTACTTCGTCGTGAGC	310	11	4407674	4407704	*gid*	1.6524E-06	2.31776E-06
rs229	kmer16763993	CATCACACGCCGGTGCTCCCGTACTTCGTCG	310	11	4407669	4407699	*gid*	1.6524E-06	2.31776E-06
rs229	kmer18726627	CCCGATGCGATCATCACACGCCGGTGCTCCC	310	11	4407658	4407688	*gid*	1.6524E-06	2.31776E-06
rs229	kmer19509795	CCGATGCGATCATCACACGCCGGTGCTCCCG	310	11	4407659	4407689	*gid*	1.6524E-06	2.31776E-06
rs229	kmer20458822	CCGGTGCTCCCGTACTTCGTCGTGAGCCCGC	310	11	4407678	4407708	*gid*	1.6524E-06	2.31776E-06
rs229	kmer21622226	CGAAGTACGGGAGCACCGGCGTGTGATGATC	310	11	4407696	4407666	*gid*	1.6524E-06	2.31776E-06
rs229	kmer22203970	CGAGCGGGCTCACGACGAAGTACGGGAGCAC	310	11	4407711	4407681	*gid*	1.6524E-06	2.31776E-06
rs229	kmer22430891	CGATCATCACACGCCGGTGCTCCCGTACTTC	310	11	4407665	4407695	*gid*	1.6524E-06	2.31776E-06
rs229	kmer23191426	CGCCGGTGCTCCCGTACTTCGTCGTGAGCCC	310	11	4407676	4407706	*gid*	1.6524E-06	2.31776E-06
rs229	kmer24792033	CGGGCTCACGACGAAGTACGGGAGCACCGGC	310	11	4407707	4407677	*gid*	1.6524E-06	2.31776E-06
rs229	kmer29269604	GAGCGGGCTCACGACGAAGTACGGGAGCACC	310	11	4407710	4407680	*gid*	1.6524E-06	2.31776E-06
rs229	kmer30152690	GCCCGATGCGATCATCACACGCCGGTGCTCC	310	11	4407657	4407687	*gid*	1.6524E-06	2.31776E-06
rs229	kmer30839817	GCTCCCGTACTTCGTCGTGAGCCCGCTCGCC	310	11	4407683	4407713	*gid*	1.6524E-06	2.31776E-06
rs229	kmer32272728	TACGGGAGCACCGGCGTGTGATGATCGCATC	310	11	4407691	4407661	*gid*	1.6524E-06	2.31776E-06
rs229	kmer33310104	TCATCACACGCCGGTGCTCCCGTACTTCGTC	310	11	4407668	4407698	*gid*	1.6524E-06	2.31776E-06
rs229	kmer35746990	TGCTCCCGTACTTCGTCGTGAGCCCGCTCGC	310	11	4407682	4407712	*gid*	1.6524E-06	2.31776E-06
rs230	kmer12721234	ATGCGATCATCACACGCCGGTGCTACCGTAC	7	4	4407662	4407692	*gid*	1.6524E-06	2.31776E-06
rs230	kmer32277649	TACGGTAGCACCGGCGTGTGATGATCGCATC	7	4	4407691	4407661	*gid*	1.6524E-06	2.31776E-06
rs44	kmer4635999	ACCACTAGCGCGGTCGCTATCACGGATACCA	11	6	876894	876924	*emrB*	1.1445E-10	8.57904E-08
rs44	kmer5673735	ACCTTTCGCACGCCTACGCGGTGGTATCCGT	11	6	876945	876915	*emrB*	1.1445E-10	8.57904E-08
rs44	kmer6167939	ACGCCTACGCGGTGGTATCCGTGATAGCGAC	11	6	876936	876906	*emrB*	1.1445E-10	8.57904E-08
rs44	kmer6268732	ACGCGGTGGTATCCGTGATAGCGACCGCGCT	11	6	876930	876900	*emrB*	1.1445E-10	8.57904E-08
rs44	kmer6944984	ACTAGCGCGGTCGCTATCACGGATACCACCG	11	6	876897	876927	*emrB*	1.1445E-10	8.57904E-08
rs44	kmer7700944	AGACCACTAGCGCGGTCGCTATCACGGATAC	11	6	876892	876922	*emrB*	1.1445E-10	8.57904E-08
rs44	kmer11335474	ATCACGGATACCACCGCGTAGGCGTGCGAAA	11	6	876912	876942	*emrB*	1.1445E-10	8.57904E-08
rs44	kmer11698312	ATCCGTGATAGCGACCGCGCTAGTGGTCTCG	11	6	876917	876890	*emrB*	1.1445E-10	8.57904E-08
rs44	kmer12431661	ATGACCTTTCGCACGCCTACGCGGTGGTATC	11	6	876948	876919	*emrB*	1.1445E-10	8.57904E-08
rs44	kmer15366074	CACGCCTACGCGGTGGTATCCGTGATAGCGA	11	6	876937	876907	*emrB*	1.1445E-10	8.57904E-08
rs44	kmer15421468	CACGGATACCACCGCGTAGGCGTGCGAAAGG	11	6	876914	876944	*emrB*	1.1445E-10	8.57904E-08
rs44	kmer15558693	CACTAGCGCGGTCGCTATCACGGATACCACC	11	6	876896	876926	*emrB*	1.1445E-10	8.57904E-08
rs44	kmer17764184	CCACTAGCGCGGTCGCTATCACGGATACCAC	11	6	876895	876925	*emrB*	1.1445E-10	8.57904E-08
rs44	kmer20634591	CCGTGATAGCGACCGCGCTAGTGGTCTCGAC	11	6	876917	876888	*emrB*	1.1445E-10	8.57904E-08
rs44	kmer20761444	CCTACGCGGTGGTATCCGTGATAGCGACCGC	11	6	876933	876903	*emrB*	1.1445E-10	8.57904E-08
rs44	kmer22695473	CGCACGCCTACGCGGTGGTATCCGTGATAGC	11	6	876939	876909	*emrB*	1.1445E-10	8.57904E-08
rs44	kmer23238193	CGCCTACGCGGTGGTATCCGTGATAGCGACC	11	6	876935	876905	*emrB*	1.1445E-10	8.57904E-08
rs44	kmer23689079	CGCGGTGGTATCCGTGATAGCGACCGCGCTA	11	6	876929	876899	*emrB*	1.1445E-10	8.57904E-08

rs44	kmer23772176	CGCTATCACGGATACCACCGCGTAGGCGTGC	11	6	876908	876938	*emrB*	1.1445E-10	8.57904E-08
rs44	kmer24128006	CGGATACCACCGCGTAGGCGTGCGAAAGGTC	11	6	876916	876946	*emrB*	1.1445E-10	8.57904E-08
rs44	kmer25004663	CGGTCGCTATCACGGATACCACCGCGTAGGC	11	6	876904	876934	*emrB*	1.1445E-10	8.57904E-08
rs44	kmer26033219	CTACGCGGTGGTATCCGTGATAGCGACCGCG	11	6	876932	876902	*emrB*	1.1445E-10	8.57904E-08
rs44	kmer26082868	CTAGCGCGGTCGCTATCACGGATACCACCGC	11	6	876898	876928	*emrB*	1.1445E-10	8.57904E-08
rs44	kmer26121921	CTATCACGGATACCACCGCGTAGGCGTGCGA	11	6	876910	876940	*emrB*	1.1445E-10	8.57904E-08
rs44	kmer28331985	CTTTCGCACGCCTACGCGGTGGTATCCGTGA	11	6	876943	876913	*emrB*	1.1445E-10	8.57904E-08
rs44	kmer28797794	GACCACTAGCGCGGTCGCTATCACGGATACC	11	6	876893	876923	*emrB*	1.1445E-10	8.57904E-08
rs44	kmer32254668	TACGCGGTGGTATCCGTGATAGCGACCGCGC	11	6	876931	876901	*emrB*	1.1445E-10	8.57904E-08
rs44	kmer32661995	TATCACGGATACCACCGCGTAGGCGTGCGAA	11	6	876911	876941	*emrB*	1.1445E-10	8.57904E-08
rs44	kmer32692745	TATCCGTGATAGCGACCGCGCTAGTGGTCTC	11	6	876921	876891	*emrB*	1.1445E-10	8.57904E-08
rs44	kmer33827064	TCCGTGATAGCGACCGCGCTAGTGGTCTCGA	11	6	876917	876889	*emrB*	1.1445E-10	8.57904E-08
rs44	kmer35191025	TGACCTTTCGCACGCCTACGCGGTGGTATCC	11	6	876947	876919	*emrB*	1.1445E-10	8.57904E-08
rs71	kmer4593341	ACCACCCCGGAACGGCTGCGGTCTCTGGAAC	306	9	1789743	1789713	*Rv1588c*	1.14449E-10	8.579E-08
rs71	kmer17623994	CCACCCCGGAACGGCTGCGGTCTCTGGAACG	306	9	1789742	1789712	*Rv1588c*	1.14449E-10	8.579E-08
rs71	kmer33483815	TCCAGAGACCGCAGCCGTTCCGGGGTGGTCA	306	9	1789715	1789745	*Rv1588c*	1.14449E-10	8.579E-08
rs71	kmer36730610	TTCCAGAGACCGCAGCCGTTCCGGGGTGGTC	306	9	1789714	1789744	*Rv1588c*	1.14449E-10	8.579E-08
rs186	kmer2219604	AAGCCATTGCGTACCGCGTCCTCGGCCGTCT	316	13	2288790	2288820	*pncA*	4.45749E-07	4.43668E-07
rs186	kmer5094323	ACCGATCATTGTGTGCGCCAGACGGCCGAGG	316	13	2288839	2288809	*pncA*	4.45749E-07	4.43668E-07
rs186	kmer5216775	ACCGCGTCCTCGGCCGTCTGGCGCACACAAT	316	13	2288802	2288832	*pncA*	4.45749E-07	4.43668E-07
rs186	kmer8474231	AGCCATTGCGTACCGCGTCCTCGGCCGTCTG	316	13	2288791	2288821	*pncA*	4.45749E-07	4.43668E-07
rs186	kmer11493018	ATCATTGTGTGCGCCAGACGGCCGAGGACGC	316	13	2288835	2288805	*pncA*	4.45749E-07	4.43668E-07
rs186	kmer13651897	ATTGCCACCGATCATTGTGTGCGCCAGACGG	316	13	2288845	2288815	*pncA*	4.45749E-07	4.43668E-07
rs186	kmer13674656	ATTGCGTACCGCGTCCTCGGCCGTCTGGCGC	316	13	2288795	2288825	*pncA*	4.45749E-07	4.43668E-07
rs186	kmer15064723	CACCGATCATTGTGTGCGCCAGACGGCCGAG	316	13	2288840	2288810	*pncA*	4.45749E-07	4.43668E-07
rs186	kmer17348098	CATTGCGTACCGCGTCCTCGGCCGTCTGGCG	316	13	2288794	2288824	*pncA*	4.45749E-07	4.43668E-07
rs186	kmer17365166	CATTGTGTGCGCCAGACGGCCGAGGACGCGG	316	13	2288833	2288803	*pncA*	4.45749E-07	4.43668E-07
rs186	kmer17641470	CCACCGATCATTGTGTGCGCCAGACGGCCGA	316	13	2288841	2288811	*pncA*	4.45749E-07	4.43668E-07
rs186	kmer17814934	CCAGACGGCCGAGGACGCGGTACGCAATGGC	316	13	2288822	2288792	*pncA*	4.45749E-07	4.43668E-07
rs186	kmer18214397	CCATTGCGTACCGCGTCCTCGGCCGTCTGGC	316	13	2288793	2288823	*pncA*	4.45749E-07	4.43668E-07
rs186	kmer19477140	CCGATCATTGTGTGCGCCAGACGGCCGAGGA	316	13	2288838	2288808	*pncA*	4.45749E-07	4.43668E-07
rs186	kmer22435194	CGATCATTGTGTGCGCCAGACGGCCGAGGAC	316	13	2288837	2288807	*pncA*	4.45749E-07	4.43668E-07
rs186	kmer23711999	CGCGTCCTCGGCCGTCTGGCGCACACAATGA	316	13	2288804	2288834	*pncA*	4.45749E-07	4.43668E-07
rs186	kmer24364303	CGGCCGTCTGGCGCACACAATGATCGGTGGC	316	13	2288812	2288842	*pncA*	4.45749E-07	4.43668E-07
rs186	kmer25186320	CGTACCGCGTCCTCGGCCGTCTGGCGCACAC	316	13	2288799	2288829	*pncA*	4.45749E-07	4.43668E-07
rs186	kmer25366455	CGTCCTCGGCCGTCTGGCGCACACAATGATC	316	13	2288806	2288836	*pncA*	4.45749E-07	4.43668E-07
rs186	kmer25566175	CGTCTGGCGCACACAATGATCGGTGGCAATA	316	13	2288816	2288846	*pncA*	4.45749E-07	4.43668E-07
rs186	kmer27564532	CTGGCGCACACAATGATCGGTGGCAATACCG	316	13	2288819	2288849	*pncA*	4.45749E-07	4.43668E-07
rs186	kmer31367114	GTATTGCCACCGATCATTGTGTGCGCCAGAC	316	13	2288847	2288817	*pncA*	4.45749E-07	4.43668E-07
rs186	kmer32181818	TACCGCGTCCTCGGCCGTCTGGCGCACACAA	316	13	2288801	2288831	*pncA*	4.45749E-07	4.43668E-07
rs186	kmer34524664	TCGGTATTGCCACCGATCATTGTGTGCGCCA	316	13	2288850	2288820	*pncA*	4.45749E-07	4.43668E-07
rs186	kmer34921438	TCTGGCGCACACAATGATCGGTGGCAATACC	316	13	2288818	2288848	*pncA*	4.45749E-07	4.43668E-07
rs186	kmer35488990	TGCCACCGATCATTGTGTGCGCCAGACGGCC	316	13	2288843	2288813	*pncA*	4.45749E-07	4.43668E-07
rs186	kmer35715110	TGCGTACCGCGTCCTCGGCCGTCTGGCGCAC	316	13	2288797	2288827	*pncA*	4.45749E-07	4.43668E-07
rs186	kmer36278116	TGTGCGCCAGACGGCCGAGGACGCGGTACGC	316	13	2288828	2288798	*pncA*	4.45749E-07	4.43668E-07
rs186	kmer36306019	TGTGTGCGCCAGACGGCCGAGGACGCGGTAC	316	13	2288830	2288800	*pncA*	4.45749E-07	4.43668E-07
rs186	kmer37127475	TTGCCACCGATCATTGTGTGCGCCAGACGGC	316	13	2288844	2288814	*pncA*	4.45749E-07	4.43668E-07

rs186	kmer37168389	TTGCGTACCGCGTCCTCGGCCGTCTGGCGCA	316	13	2288796	2288826	*pncA*	4.45749E-07	4.43668E-07
**KwaZulu-Natal's study population**									
rs18	kmer26323394	CTGGCTGCCCAGCGAGCTCATCGCCCGGACC	47	52	1340230	1340203	*PPE18*	1.71063E-37	2.99814E-08
rs18	kmer28929848	GGCTGCCCAGCGAGCTCATCGCCCGGACCCC	47	52	1340230	1340201	*PPE18*	1.71063E-37	2.99814E-08
rs18	kmer33862222	TGGCTGCCCAGCGAGCTCATCGCCCGGACCC	47	52	1340230	1340202	*PPE18*	1.71063E-37	2.99814E-08
rs18	kmer28784798	GCTGGCTGCCCAGCGAGCTCATCGCCCGGAC	47	52	1532751	1532781	*PPE19*	1.71063E-37	2.99814E-08
rs18	kmer11068324	AGCTGGCTGCCCAGCGAGCTCATCGCCCGGA	47	52	1532750	1532780	*PPE19*	1.71063E-37	2.99814E-08
rs18	kmer20785593	CCAGCTGGCTGCCCAGCGAGCTCATCGCCCG	47	52	3895311	3895282	*PPE60**	1.71063E-37	2.99814E-08
rs18	kmer19137052	CAGCTGGCTGCCCAGCGAGCTCATCGCCCGG	47	52	3895310	3895282	*PPE60*	1.71063E-37	2.99814E-08
rs18	kmer24462647	CGGTGCGGCGCAGTTCAGTAGATGCCGGTCC	47	52	4408043	4408068	*gid*	1.71063E-37	2.99814E-08
rs18	kmer7656521	ACCGGCATCTACTGAACTGCGCCGCACCGAG	47	52	4408066	4408043	*gid*	1.71063E-37	2.99814E-08
rs18	kmer30275354	TACTGAACTGCGCCGCACCGAGTTTCTTCGA	47	52	4407921	4407897	*gid*	1.71063E-37	2.99814E-08
rs18	kmer25941220	CTGAACTGCGCCGCACCGAGTTTCTTCGAGA	47	52	4407921	4407895	*gid*	1.71063E-37	2.99814E-08
rs18	kmer32806171	TCTCTCGAAGAAACTCGGTGCGGCGCAGTTC	47	52	4407893	4407921	*gid*	1.71063E-37	2.99814E-08
rs18	kmer33875223	TGGGACCGGCATCTACTGAACTGCGCCGCAC	47	52	4408070	4408043	*gid*	1.71063E-37	2.99814E-08
rs18	kmer32536711	TCGGTGCGGCGCAGTTCAGTAGATGCCGGTC	47	52	4408043	4408067	*gid*	1.71063E-37	2.99814E-08
rs18	kmer3113753	AACTGCGCCGCACCGAGTTTCTTCGAGAGAT	47	52	4407921	4407892	*gid*	1.71063E-37	2.99814E-08
rs18	kmer25839097	CTCTCGAAGAAACTCGGTGCGGCGCAGTTCA	47	52	4407894	4407921	*gid*	1.71063E-37	2.99814E-08
rs18	kmer25215189	CTACTGAACTGCGCCGCACCGAGTTTCTTCG	47	52	4407921	4407898	*gid*	1.71063E-37	2.99814E-08
rs18	kmer28948245	GGTGCGGCGCAGTTCAGTAGATGCCGGTCCC	47	52	4408043	4408069	*gid*	1.71063E-37	2.99814E-08
rs18	kmer3071807	AACTCGGTGCGGCGCAGTTCAGTAGATGCCG	47	52	4408043	4408064	*gid*	1.71063E-37	2.99814E-08
rs18	kmer8983053	ACTCGGTGCGGCGCAGTTCAGTAGATGCCGG	47	52	4408043	4408065	*gid*	1.71063E-37	2.99814E-08
rs18	kmer32691685	TCTACTGAACTGCGCCGCACCGAGTTTCTTC	47	52	4407921	4407899	*gid*	1.71063E-37	2.99814E-08
rs18	kmer27350510	GAAACTCGGTGCGGCGCAGTTCAGTAGATGC	47	52	4407902	4407921	*gid*	1.71063E-37	2.99814E-08
rs18	kmer3272659	AAGAAACTCGGTGCGGCGCAGTTCAGTAGAT	47	52	4407900	4407921	*gid*	1.71063E-37	2.99814E-08
rs18	kmer9033433	ACTGAACTGCGCCGCACCGAGTTTCTTCGAG	47	52	4407921	4407896	*gid*	1.71063E-37	2.99814E-08
rs18	kmer9522910	AGAAACTCGGTGCGGCGCAGTTCAGTAGATG	47	52	4407901	4407921	*gid*	1.71063E-37	2.99814E-08
rs18	kmer15334460	ATGGGACCGGCATCTACTGAACTGCGCCGCA	47	52	4408071	4408043	*gid*	1.71063E-37	2.99814E-08
rs18	kmer1051894	AAACTCGGTGCGGCGCAGTTCAGTAGATGCC	47	52	4408043	4408063	*gid*	1.71063E-37	2.99814E-08
rs18	kmer31029355	TATGGGACCGGCATCTACTGAACTGCGCCGC	47	52	4408072	4408043	*gid*	1.71063E-37	2.99814E-08
rs35	kmer10995207	AGCTCCTCGAACGCGGTGACCGGGTCGTGGA	232	4	4408033	4408003	*gid*	3.98215E-36	7.80205E-07
rs35	kmer29121848	GTGATCGGTGAGCTCCTCGAACGCGGTGACC	232	4	4408043	4408013	*gid*	3.98215E-36	7.80205E-07
rs35	kmer19844985	CATCTACTGAACTGCGCCGTGATCGGTGAGC	232	4	4408061	4408031	*gid*	3.98215E-36	7.80205E-07
rs35	kmer25467783	CTCACCGATCACGGCGCAGTTCAGTAGATGC	232	4	4408032	4408062	*gid*	3.98215E-36	7.80205E-07
rs35	kmer14422788	ATCTACTGAACTGCGCCGTGATCGGTGAGCT	232	4	4408060	4408030	*gid*	3.98215E-36	7.80205E-07
rs35	kmer25217263	CTACTGCGCCGCACCGAGTTTCTTCGAGAGA	232	4	4407923	4407893	*gid*	3.98215E-36	7.80205E-07
rs35	kmer32300297	TCGAGGAGCTCACCGATCACGGCGCAGTTCA	232	4	4408024	4408054	*gid*	3.98215E-36	7.80205E-07
rs35	kmer8444806	ACGGCGCAGTTCAGTAGATGCCGGTCCCATA	232	4	4408042	4408072	*gid*	3.98215E-36	7.80205E-07
rs35	kmer24450176	CGGTGAGCTCCTCGAACGCGGTGACCGGGTC	232	4	4408038	4408008	*gid*	3.98215E-36	7.80205E-07
rs35	kmer17468766	CAACCCGGCTCCGCTACCGATATCCACGACC	232	4	4407981	4408011	*gid*	3.98215E-36	7.80205E-07
rs35	kmer18359953	CACCGATCACGGCGCAGTTCAGTAGATGCCG	232	4	4408034	4408064	*gid*	3.98215E-36	7.80205E-07
rs35	kmer2832637	AACGCGGTGACCGGGTCGTGGATATCGGTAG	232	4	4408024	4407994	*gid*	3.98215E-36	7.80205E-07
rs35	kmer25941222	CTGAACTGCGCCGTGATCGGTGAGCTCCTCG	232	4	4408055	4408025	*gid*	3.98215E-36	7.80205E-07
rs35	kmer33211610	TGACCGGGTCGTGGATATCGGTAGCGGAGCC	232	4	4408017	4407987	*gid*	3.98215E-36	7.80205E-07
rs35	kmer28724483	GCTCCGCTACCGATATCCACGACCCGGTCAC	232	4	4407988	4408018	*gid*	3.98215E-36	7.80205E-07
rs35	kmer24416774	CGGTCACCGCGTTCGAGGAGCTCACCGATCA	232	4	4408012	4408042	*gid*	3.98215E-36	7.80205E-07
rs35	kmer9094311	ACTGCGCCGTGATCGGTGAGCTCCTCGAACG	232	4	4408051	4408021	*gid*	3.98215E-36	7.80205E-07

rs35	kmer13272568	ATATCCACGACCCGGTCACCGCGTTCGAGGA	232	4	4408000	4408030	*gid*	3.98215E-36	7.80205E-07
rs35	kmer13675032	ATCACGGCGCAGTTCAGTAGATGCCGGTCCC	232	4	4408039	4408069	*gid*	3.98215E-36	7.80205E-07
rs35	kmer21854857	CCGCGTTCGAGGAGCTCACCGATCACGGCGC	232	4	4408018	4408048	*gid*	3.98215E-36	7.80205E-07
rs35	kmer23207533	CGATCACGGCGCAGTTCAGTAGATGCCGGTC	232	4	4408037	4408067	*gid*	3.98215E-36	7.80205E-07
rs35	kmer2579962	AACCCGGCTCCGCTACCGATATCCACGACCC	232	4	4407982	4408012	*gid*	3.98215E-36	7.80205E-07
rs35	kmer24447073	CGGTGACCGGGTCGTGGATATCGGTAGCGGA	232	4	4408020	4407990	*gid*	3.98215E-36	7.80205E-07
rs35	kmer22052563	CCGGTCACCGCGTTCGAGGAGCTCACCGATC	232	4	4408011	4408041	*gid*	3.98215E-36	7.80205E-07
rs35	kmer9033434	ACTGAACTGCGCCGTGATCGGTGAGCTCCTC	232	4	4408056	4408026	*gid*	3.98215E-36	7.80205E-07
rs35	kmer20624967	CCACGACCCGGTCACCGCGTTCGAGGAGCTC	232	4	4408004	4408034	*gid*	3.98215E-36	7.80205E-07
rs35	kmer18475391	CACGACCCGGTCACCGCGTTCGAGGAGCTCA	232	4	4408005	4408035	*gid*	3.98215E-36	7.80205E-07
rs35	kmer25651697	CTCCTCGAACGCGGTGACCGGGTCGTGGATA	232	4	4408031	4408001	*gid*	3.98215E-36	7.80205E-07
rs35	kmer23823071	CGCTACCGATATCCACGACCCGGTCACCGCG	232	4	4407992	4408022	*gid*	3.98215E-36	7.80205E-07
rs35	kmer13887590	ATCCACGACCCGGTCACCGCGTTCGAGGAGC	232	4	4408002	4408032	*gid*	3.98215E-36	7.80205E-07
rs35	kmer24775424	CGTGATCGGTGAGCTCCTCGAACGCGGTGAC	232	4	4408044	4408014	*gid*	3.98215E-36	7.80205E-07
rs35	kmer18379647	CACCGCGTTCGAGGAGCTCACCGATCACGGC	232	4	4408016	4408046	*gid*	3.98215E-36	7.80205E-07
rs35	kmer21860509	CCGCTACTGCGCCGCACCGAGTTTCTTCGAG	232	4	4407926	4407896	*gid*	3.98215E-36	7.80205E-07
rs35	kmer3113816	AACTGCGCCGTGATCGGTGAGCTCCTCGAAC	232	4	4408052	4408022	*gid*	3.98215E-36	7.80205E-07
rs35	kmer22157426	CCGTGATCGGTGAGCTCCTCGAACGCGGTGA	232	4	4408045	4408015	*gid*	3.98215E-36	7.80205E-07
rs35	kmer25682750	CTCGAACGCGGTGACCGGGTCGTGGATATCG	232	4	4408028	4407998	*gid*	3.98215E-36	7.80205E-07
rs35	kmer22364440	CCTCGAACGCGGTGACCGGGTCGTGGATATC	232	4	4408029	4407999	*gid*	3.98215E-36	7.80205E-07
rs35	kmer8305557	ACGCGGTGACCGGGTCGTGGATATCGGTAGC	232	4	4408023	4407993	*gid*	3.98215E-36	7.80205E-07
rs35	kmer7656522	ACCGGCATCTACTGAACTGCGCCGTGATCGG	232	4	4408066	4408036	*gid*	3.98215E-36	7.80205E-07
rs35	kmer7472264	ACCCGGCTCCGCTACCGATATCCACGACCCG	232	4	4407983	4408013	*gid*	3.98215E-36	7.80205E-07
rs35	kmer24255661	CGGCTCCGCTACCGATATCCACGACCCGGTC	232	4	4407986	4408016	*gid*	3.98215E-36	7.80205E-07
rs35	kmer30094335	TACCGATATCCACGACCCGGTCACCGCGTTC	232	4	4407995	4408025	*gid*	3.98215E-36	7.80205E-07
rs35	kmer28200671	GCAACCCGGCTCCGCTACCGATATCCACGAC	232	4	4407980	4408010	*gid*	3.98215E-36	7.80205E-07
rs35	kmer21386852	CCCGGCTCCGCTACCGATATCCACGACCCGG	232	4	4407984	4408014	*gid*	3.98215E-36	7.80205E-07
rs35	kmer22960879	CGACCCGGTCACCGCGTTCGAGGAGCTCACC	232	4	4408007	4408037	*gid*	3.98215E-36	7.80205E-07
rs35	kmer7557963	ACCGATCACGGCGCAGTTCAGTAGATGCCGG	232	4	4408035	4408065	*gid*	3.98215E-36	7.80205E-07
rs35	kmer7556618	ACCGATATCCACGACCCGGTCACCGCGTTCG	232	4	4407996	4408026	*gid*	3.98215E-36	7.80205E-07
rs35	kmer25215190	CTACTGAACTGCGCCGTGATCGGTGAGCTCC	232	4	4408058	4408028	*gid*	3.98215E-36	7.80205E-07
rs35	kmer21859301	CCGCTACCGATATCCACGACCCGGTCACCGC	232	4	4407991	4408021	*gid*	3.98215E-36	7.80205E-07
rs35	kmer32691686	TCTACTGAACTGCGCCGTGATCGGTGAGCTC	232	4	4408059	4408029	*gid*	3.98215E-36	7.80205E-07
rs35	kmer8028042	ACGACCCGGTCACCGCGTTCGAGGAGCTCAC	232	4	4408006	4408036	*gid*	3.98215E-36	7.80205E-07
rs35	kmer31517767	TCACGGCGCAGTTCAGTAGATGCCGGTCCCA	232	4	4408040	4408070	*gid*	3.98215E-36	7.80205E-07
rs35	kmer7623096	ACCGCGTTCGAGGAGCTCACCGATCACGGCG	232	4	4408017	4408047	*gid*	3.98215E-36	7.80205E-07
rs35	kmer25839096	CTCTCGAAGAAACTCGGTGCGGCGCAGTAGC	232	4	4407894	4407924	*gid*	3.98215E-36	7.80205E-07
rs35	kmer14316614	ATCGGTGAGCTCCTCGAACGCGGTGACCGGG	232	4	4408040	4408010	*gid*	3.98215E-36	7.80205E-07
rs35	kmer11276695	AGGAGCTCACCGATCACGGCGCAGTTCAGTA	232	4	4408027	4408057	*gid*	3.98215E-36	7.80205E-07
rs35	kmer7475958	ACCCGGTCACCGCGTTCGAGGAGCTCACCGA	232	4	4408009	4408039	*gid*	3.98215E-36	7.80205E-07
rs35	kmer23809121	CGCGTTCGAGGAGCTCACCGATCACGGCGCA	232	4	4408019	4408049	*gid*	3.98215E-36	7.80205E-07
rs35	kmer34975438	TTCGAGGAGCTCACCGATCACGGCGCAGTTC	232	4	4408023	4408053	*gid*	3.98215E-36	7.80205E-07
rs35	kmer31486866	TCACCGATCACGGCGCAGTTCAGTAGATGCC	232	4	4408033	4408063	*gid*	3.98215E-36	7.80205E-07
rs35	kmer26159179	CTGCGCCGTGATCGGTGAGCTCCTCGAACGC	232	4	4408050	4408020	*gid*	3.98215E-36	7.80205E-07
rs35	kmer21649742	CCGATATCCACGACCCGGTCACCGCGTTCGA	232	4	4407997	4408027	*gid*	3.98215E-36	7.80205E-07
rs35	kmer27995034	GATCACGGCGCAGTTCAGTAGATGCCGGTCC	232	4	4408038	4408068	*gid*	3.98215E-36	7.80205E-07
rs35	kmer25624087	CTCCGCTACCGATATCCACGACCCGGTCACC	232	4	4407989	4408019	*gid*	3.98215E-36	7.80205E-07

rs35	kmer7683239	ACCGGGTCGTGGATATCGGTAGCGGAGCCGG	232	4	4408015	4407985	*gid*	3.98215E-36	7.80205E-07
rs35	kmer14489606	ATCTCTCGAAGAAACTCGGTGCGGCGCAGTA	232	4	4407892	4407922	*gid*	3.98215E-36	7.80205E-07
rs35	kmer15334461	ATGGGACCGGCATCTACTGAACTGCGCCGTG	232	4	4408071	4408041	*gid*	3.98215E-36	7.80205E-07
rs35	kmer23826036	CGCTACTGCGCCGCACCGAGTTTCTTCGAGA	232	4	4407925	4407895	*gid*	3.98215E-36	7.80205E-07
rs56	kmer32611629	TCGTGGATATCGGTAGCGGAGCCGGGTTGCC	231	4	4408009	4407979	*gid*	1.79671E-35	3.74798E-06
rs56	kmer24812225	CGTGGATATCGGTAGCGGAGCCGGGTTGCCG	231	4	4408008	4407978	*gid*	1.79671E-35	3.74798E-06
rs56	kmer18512612	CACGCCCGGCAACCCGGCTCCGCTACCGATA	231	4	4407972	4408002	*gid*	1.79671E-35	3.74798E-06
rs56	kmer21945093	CCGGCAACCCGGCTCCGCTACCGATATCCAC	231	4	4407977	4408007	*gid*	1.79671E-35	3.74798E-06
rs56	kmer21374479	CCCGGCAACCCGGCTCCGCTACCGATATCCA	231	4	4407976	4408006	*gid*	1.79671E-35	3.74798E-06
rs56	kmer23558993	CGCCCGGCAACCCGGCTCCGCTACCGATATC	231	4	4407974	4408004	*gid*	1.79671E-35	3.74798E-06
rs56	kmer15275280	ATGGCACGCCCGGCAACCCGGCTCCGCTACC	231	4	4407968	4407998	*gid*	1.79671E-35	3.74798E-06
rs56	kmer28445897	GCCCGGCAACCCGGCTCCGCTACCGATATCC	231	4	4407975	4408005	*gid*	1.79671E-35	3.74798E-06
rs56	kmer8241944	ACGCCCGGCAACCCGGCTCCGCTACCGATAT	231	4	4407973	4408003	*gid*	1.79671E-35	3.74798E-06
rs56	kmer24402640	CGGTAGCGGAGCCGGGTTGCCGGGCGTGCCA	231	4	4407999	4407969	*gid*	1.79671E-35	3.74798E-06
rs56	kmer14310967	ATCGGTAGCGGAGCCGGGTTGCCGGGCGTGC	231	4	4408001	4407971	*gid*	1.79671E-35	3.74798E-06
rs56	kmer32526264	TCGGTAGCGGAGCCGGGTTGCCGGGCGTGCC	231	4	4408000	4407970	*gid*	1.79671E-35	3.74798E-06
rs34	kmer33461786	TGCAGTAGCTATGATTTCGCTGTGATTGAAC	49	52	877049	877079	*emrB*	3.98216E-36	7.80206E-07
rs34	kmer19030115	CAGCGAAATCATAGCTACTGCAAAGAAAGTC	49	52	877070	877040	*emrB*	3.98216E-36	7.80206E-07
rs34	kmer34722663	TTCAATCACAGCGAAATCATAGCTACTGCAA	49	52	877078	877048	*emrB*	3.98216E-36	7.80206E-07
rs34	kmer19343715	CAGTAGCTATGATTTCGCTGTGATTGAACTG	49	52	877051	877081	*emrB*	3.98216E-36	7.80206E-07
rs34	kmer31413181	TCAATCACAGCGAAATCATAGCTACTGCAAA	49	52	877077	877047	*emrB*	3.98216E-36	7.80206E-07
rs34	kmer11852961	AGTAGCTATGATTTCGCTGTGATTGAACTGG	49	52	877052	877082	*emrB*	3.98216E-36	7.80206E-07
rs34	kmer5020707	AATCATAGCTACTGCAAAGAAAGTCGCACTG	49	52	877064	877034	*emrB*	3.98216E-36	7.80206E-07
rs34	kmer28552159	GCGAAATCATAGCTACTGCAAAGAAAGTCGC	49	52	877068	877038	*emrB*	3.98216E-36	7.80206E-07
rs34	kmer27376253	GAAATCATAGCTACTGCAAAGAAAGTCGCAC	49	52	877066	877036	*emrB*	3.98216E-36	7.80206E-07
rs34	kmer19567092	CATAGCTACTGCAAAGAAAGTCGCACTGACC	49	52	877061	877031	*emrB*	3.98216E-36	7.80206E-07
rs34	kmer7302678	ACCAGTTCAATCACAGCGAAATCATAGCTAC	49	52	877083	877053	*emrB*	3.98216E-36	7.80206E-07
rs34	kmer10733568	AGCGAAATCATAGCTACTGCAAAGAAAGTCG	49	52	877069	877039	*emrB*	3.98216E-36	7.80206E-07
rs34	kmer10956116	AGCTACTGCAAAGAAAGTCGCACTGACCCCA	49	52	877057	877028	*emrB*	3.98216E-36	7.80206E-07
rs34	kmer30065015	TACCAGTTCAATCACAGCGAAATCATAGCTA	49	52	877084	877054	*emrB*	3.98216E-36	7.80206E-07
rs34	kmer22774386	CGAAATCATAGCTACTGCAAAGAAAGTCGCA	49	52	877067	877037	*emrB*	3.98216E-36	7.80206E-07
rs34	kmer18417903	CACCTACCAGTTCAATCACAGCGAAATCATA	49	52	877088	877059	*emrB*	3.98216E-36	7.80206E-07
rs34	kmer31667133	TCATAGCTACTGCAAAGAAAGTCGCACTGAC	49	52	877062	877032	*emrB*	3.98216E-36	7.80206E-07
rs34	kmer22252405	CCTACCAGTTCAATCACAGCGAAATCATAGC	49	52	877086	877056	*emrB*	3.98216E-36	7.80206E-07
rs34	kmer6741385	ACAGCGAAATCATAGCTACTGCAAAGAAAGT	49	52	877071	877041	*emrB*	3.98216E-36	7.80206E-07
rs34	kmer7762141	ACCTACCAGTTCAATCACAGCGAAATCATAG	49	52	877087	877059	*emrB*	3.98216E-36	7.80206E-07
rs34	kmer18192168	CACAGCGAAATCATAGCTACTGCAAAGAAAG	49	52	877072	877042	*emrB*	3.98216E-36	7.80206E-07
rs34	kmer12213221	AGTTCAATCACAGCGAAATCATAGCTACTGC	49	52	877080	877050	*emrB*	3.98216E-36	7.80206E-07
rs34	kmer13066302	ATAGCTACTGCAAAGAAAGTCGCACTGACCC	49	52	877057	877030	*emrB*	3.98216E-36	7.80206E-07
rs34	kmer31460189	TCACAGCGAAATCATAGCTACTGCAAAGAAA	49	52	877073	877043	*emrB*	3.98216E-36	7.80206E-07
rs34	kmer13641656	ATCACAGCGAAATCATAGCTACTGCAAAGAA	49	52	877074	877044	*emrB*	3.98216E-36	7.80206E-07
rs34	kmer4981719	AATCACAGCGAAATCATAGCTACTGCAAAGA	49	52	877075	877045	*emrB*	3.98216E-36	7.80206E-07
rs34	kmer17908725	CAATCACAGCGAAATCATAGCTACTGCAAAG	49	52	877076	877046	*emrB*	3.98216E-36	7.80206E-07
rs34	kmer30504543	TAGCTACTGCAAAGAAAGTCGCACTGACCCC	49	52	877057	877029	*emrB*	3.98216E-36	7.80206E-07
rs34	kmer1722216	AAATCATAGCTACTGCAAAGAAAGTCGCACT	49	52	877065	877035	*emrB*	3.98216E-36	7.80206E-07
rs34	kmer13767395	ATCATAGCTACTGCAAAGAAAGTCGCACTGA	49	52	877063	877033	*emrB*	3.98216E-36	7.80206E-07
rs34	kmer10969668	AGCTATGATTTCGCTGTGATTGAACTGGTAG	49	52	877055	877085	*emrB*	3.98216E-36	7.80206E-07

rs438	kmer32458938	TCGGACCTCGTCGAGCACTCACCGGTGAACC	230	7	1789541	1789511	*Rv1588c*	4.1013E-08	2.453E-06
rs438	kmer25743678	CTCGGACCTCGTCGAGCACTCACCGGTGAAC	230	7	1789542	1789512	*Rv1588c*	4.1013E-08	2.453E-06
rs438	kmer24009119	CGGACCTCGTCGAGCACTCACCGGTGAACCG	230	7	1789540	1789510	*Rv1588c*	4.1013E-08	2.453E-06
rs439	kmer32788855	TCTCGGACCTCGTCGAGCACTCACCGGTGAA	230	7	1789543	1789513	*Rv1588c*	4.02368E-08	2.52941E-06
rs732	kmer22390942	CCTCGTCGAGCACTCACCGGTGAACCGCTAG	224	7	1789536	1789506	*Rv1588c*	2.32481E-07	3.28469E-06
rs732	kmer28662338	GCGGTTCACCGGTGAGTGCTCGACGAGGTCC	224	7	1789509	1789539	*Rv1588c*	2.32481E-07	3.28469E-06
rs732	kmer29076944	GTCGAGCACTCACCGGTGAACCGCTAGCCCC	224	7	1789532	1789502	*Rv1588c*	2.32481E-07	3.28469E-06
rs732	kmer25784527	CTCGTCGAGCACTCACCGGTGAACCGCTAGC	224	7	1789535	1789505	*Rv1588c*	2.32481E-07	3.28469E-06
rs732	kmer10892125	AGCGGTTCACCGGTGAGTGCTCGACGAGGTC	224	7	1789508	1789538	*Rv1588c*	2.32481E-07	3.28469E-06
rs732	kmer24692987	CGTCGAGCACTCACCGGTGAACCGCTAGCCC	224	7	1789533	1789503	*Rv1588c*	2.32481E-07	3.28469E-06
rs732	kmer7810546	ACCTCGTCGAGCACTCACCGGTGAACCGCTA	224	7	1789537	1789507	*Rv1588c*	2.32481E-07	3.28469E-06
rs732	kmer32585325	TCGTCGAGCACTCACCGGTGAACCGCTAGCC	224	7	1789534	1789504	*Rv1588c*	2.32481E-07	3.28469E-06
rs14	kmer11243466	AGGACGCGGTACGCAATGGCTTGGCCCACCA	23	42	2288811	2288782	*pncA*	1.71561E-17	4.8017E-11
rs14	kmer18920256	CAGCACCCTGGTGGGCCAAGCCATTGCGTAC	23	42	2288774	2288803	*pncA*	1.71561E-17	4.8017E-11
rs14	kmer7502865	ACCCTGGTGGGCCAAGCCATTGCGTACCGCG	23	42	2288778	2288807	*pncA*	1.71561E-17	4.8017E-11
rs14	kmer26381741	CTGGTGGGCCAAGCCATTGCGTACCGCGTCC	23	42	2288781	2288810	*pncA*	1.71561E-17	4.8017E-11
rs14	kmer24397829	CGGTACGCAATGGCTTGGCCCACCAGGGTGC	23	42	2288805	2288776	*pncA*	1.71561E-17	4.8017E-11
rs14	kmer21628315	CCGAGGACGCGGTACGCAATGGCTTGGCCCA	23	42	2288814	2288786	*pncA*	1.71561E-17	4.8017E-11
rs14	kmer7261938	ACCAGCACCCTGGTGGGCCAAGCCATTGCGT	23	42	2288772	2288801	*pncA*	1.71561E-17	4.8017E-11
rs14	kmer28794885	GCTGTCAGGTCCACCAGCACCCTGGTGGGCC	23	42	2288760	2288787	*pncA*	1.71561E-17	4.8017E-11
rs14	kmer5547802	AATGGCTTGGCCCACCAGGGTGCTGGTGGAC	23	42	2288797	2288768	*pncA*	1.71561E-17	4.8017E-11
rs14	kmer18283116	CACCAGCACCCTGGTGGGCCAAGCCATTGCG	23	42	2288771	2288800	*pncA*	1.71561E-17	4.8017E-11
rs14	kmer20729304	CCAGCACCCTGGTGGGCCAAGCCATTGCGTA	23	42	2288773	2288802	*pncA*	1.71561E-17	4.8017E-11
rs14	kmer31624178	TCAGGTCCACCAGCACCCTGGTGGGCCAAGC	23	42	2288764	2288793	*pncA*	1.71561E-17	4.8017E-11
rs14	kmer22533066	CCTGGTGGGCCAAGCCATTGCGTACCGCGTC	23	42	2288780	2288809	*pncA*	1.71561E-17	4.8017E-11
rs14	kmer20580821	CCACCAGCACCCTGGTGGGCCAAGCCATTGC	23	42	2288770	2288799	*pncA*	1.71561E-17	4.8017E-11
rs14	kmer28468077	GCCGAGGACGCGGTACGCAATGGCTTGGCCC	23	42	2288815	2288786	*pncA*	1.71561E-17	4.8017E-11
rs14	kmer10386069	AGCACCCTGGTGGGCCAAGCCATTGCGTACC	23	42	2288775	2288804	*pncA*	1.71561E-17	4.8017E-11
rs14	kmer11643861	AGGTCCACCAGCACCCTGGTGGGCCAAGCCA	23	42	2288766	2288795	*pncA*	1.71561E-17	4.8017E-11
rs14	kmer23116976	CGAGGACGCGGTACGCAATGGCTTGGCCCAC	23	42	2288813	2288786	*pncA*	1.71561E-17	4.8017E-11
rs14	kmer18344426	CACCCTGGTGGGCCAAGCCATTGCGTACCGC	23	42	2288777	2288806	*pncA*	1.71561E-17	4.8017E-11
rs14	kmer15327157	ATGGCTTGGCCCACCAGGGTGCTGGTGGACC	23	42	2288796	2288767	*pncA*	1.71561E-17	4.8017E-11
rs14	kmer26993962	CTTGGCCCACCAGGGTGCTGGTGGACCTGAC	23	42	2288792	2288763	*pncA*	1.71561E-17	4.8017E-11
rs14	kmer27882417	GAGGACGCGGTACGCAATGGCTTGGCCCACC	23	42	2288812	2288783	*pncA*	1.71561E-17	4.8017E-11
rs14	kmer23909802	CGCTGTCAGGTCCACCAGCACCCTGGTGGGC	23	42	2288759	2288787	*pncA*	1.71561E-17	4.8017E-11
rs14	kmer35461126	TTGGCCCACCAGGGTGCTGGTGGACCTGACA	23	42	2288791	2288762	*pncA*	1.71561E-17	4.8017E-11
rs14	kmer26433640	CTGTCAGGTCCACCAGCACCCTGGTGGGCCA	23	42	2288761	2288790	*pncA*	1.71561E-17	4.8017E-11
rs14	kmer8304203	ACGCGGTACGCAATGGCTTGGCCCACCAGGG	23	42	2288808	2288779	*pncA*	1.71561E-17	4.8017E-11
rs14	kmer19291803	CAGGTCCACCAGCACCCTGGTGGGCCAAGCC	23	42	2288765	2288794	*pncA*	1.71561E-17	4.8017E-11
rs14	kmer18004892	CAATGGCTTGGCCCACCAGGGTGCTGGTGGA	23	42	2288798	2288769	*pncA*	1.71561E-17	4.8017E-11
rs14	kmer21096931	CCCACCAGGGTGCTGGTGGACCTGACAGCGG	23	42	2288787	2288758	*pncA*	1.71561E-17	4.8017E-11

**PPE60* k-mers shown here were not distinguished from *PPE18/19* by conventional mapping

Supplementary Table S2: Five common genes (*PPE18/19, gid, emrB, pncA*, and *Rv1588c*) with their unitigs obtained by DBGWAS and their significant associations with the clustered strains carrying the *katG*-S315T mutation (cluster[+]/S315T[+] = phenotype[+]).

Gene	Component ID	Node ID	Allele frequency	Proportion [No (%)] of unitigs among phenotype (-)	Proportion [No (%)] of unitigs among phenotype (+)	Ststistical significance	q value	Est Effect	Wald Statistics	Unitig sequence	Sequence Length	Original annotation
**Hanoi sample set**												
PPE18	233	n197193	14	8/317 (2.5)	6/15 (40.0)	Yes	5.56188E-07	0.400269	7.631371082	TCACCGGCGGGGGCACCGTCAGCCCATACGTCGTCGCGTAGGCCGCCGCAGCAACCCGGACCTGGG	66	
gid	104	n150579	321	310/317 (97.8)	11/15 (73.3)	Yes	0.000740263	-0.3441218	4.752993948	AAAGGCGAGCGGGCTCACGACGAAGTACGGGAGCACCGGCGTGTGATGATCGCATCGGGC	60	(AGly)GIDB~~~[uniprot]_Ribosomal_RNA_small_subunit_methyltransferase_G~~~[uniprot]_Nitrogenase_iron-molybdenum_cofactor_biosynthesis_protein_NifN
gid	104	n248374	11	7/317 (2.2)	4/15 (26.7)	Yes	0.000740263	0.3441218	4.752993948	AAAGGCGAGCGGGCTCACGACGAAGTACGGTAGCACCGGCGTGTGATGATCGCATCGGGCG	61	(AGly)GIDB~~~[uniprot]_Ribosomal_RNA_small_subunit_methyltransferase_G~~~[uniprot]_Nitrogenase_iron-molybdenum_cofactor_biosynthesis_protein_NifN
gid	104	n252315	312	303/317 (95.6)	9/15 (60.0)	Yes	3.67586E-05	-0.2848966	5.608958218	CGCCCGATGCGATCATCACACGCCGGTGCTC	31	(AGly)GIDB~~~[uniprot]_Ribosomal_RNA_small_subunit_methyltransferase_G
emrB	202	n200809	16	10/317 (3.2)	6/15 (40.0)	Yes	1.70427E-05	0.3465184	6.949792671	ATGACCTTTCGCACGCCTACGCGGTGGTATCCGTGATAGCGACCGCGCTAGTGGTCTCGAC	61	[uniprot]_Multidrug_resistance_protein_B_homolog
emrB	202	n248807	316	307/317 (96.8)	9/15 (60.0)	Yes	1.70427E-05	-0.3465184	6.949792671	GTCGAGACCACTAGCGCGGTCGCTATCACGAATACCACCGCGTAGGCGTGCGAAAGGTCAT	61	[uniprot]_Multidrug_resistance_protein_B_homolog~~~[uniprot] Histidine--tRNA ligase
Rv1588c	44	n174787	316	307/317 (96.8)	9/15 (60.0)	Yes	1.70427E-05	-0.3465184	6.949792671	CACCCCGGAACGGCTGCGGTCTCTGGAACGTCTGGAA	37	[uniprot]_Uncharacterized_protein_Rv3466~~~[uniprot]_Uncharacterized_protein_MT3572~~~[uniprot]_Uncharacterized_protein_Mb1614c~~~[uniprot]_Uncharacterized_protein_MT1623~~~[uniprot]_Uncharacterized_protein_Rv1588c~~~[uniprot]_Putative_uncharacterized_protein_Rv0095c
Rv1588c	44	n187933	313	304/317 (95.9)	9/15 (60.0)	Yes	0.000474085	-0.2830388	6.006374372	TGACCACCCCGGAACGGCTGCGGTCTCTGGAACG	34	
**KwaZulu-Natal sample set**												
PPE18	46	n235433	99	47/281 (16.7)	52/56 (92.9)	Yes	4.8561E-10	0.5084456	14.54529434	CCAGCTGGCTGCCCAGCGAGCTCATCGCCCGGACCCC	37	
gid	136	n689117	236	232/281 (82.6)	4/56 (7.1)	Yes	7.94256E-09	-0.4931431	12.03585938	ATCTCTCGAAGAAACTCGGTGCGGCGCAGTAGCGG	35	(AGly)GIDB~~~[uniprot]_Ribosomal_RNA_small_subunit_methyltransferase_G
gid	136	n664457	99	47/281 (16.7)	52/56 (92.9)	Yes	4.8561E-10	0.5084456	14.54529434	ATCTCTCGAAGAAACTCGGTGCGGCGCAGTTCAGTAGATGCCGGTCCCATA	51	(AGly)GIDB~~~[uniprot]_Ribosomal_RNA_small_subunit_methyltransferase_G~~~[uniprot]_ATP-dependent_zinc_metalloprotease_FtsH
gid	136	n361723	235	231/281 (82.2)	4/56 (7.1)	Yes	3.0005E-08	-0.4857804	11.73377507	ATGGCACGCCCGGCAACCCGGCTCCGCTACCGATATCCACG	41	(AGly)GIDB~~~[uniprot]_Ribosomal_RNA_small_subunit_methyltransferase_G~~~[uniprot]_Ribosomal_RNA_small_subunit_methyltransferase_G_1~~~[uniprot]_Ribosomal_RNA small subunit methyltransferase G 2
gid	136	n190849	236	232/281 (82.6)	4/56 (7.1)	Yes	7.94256E-09	-0.4931431	12.03585938	CGCCGTGATCGGTGAGCTCCTCGAACGCGGTGACCGGGTCGTGGATATCGGTAGCGGAGCCGGGTTGC	68	(AGly)GIDB~~~[uniprot]_Ribosomal_RNA_small_subunit_methyltransferase_G~~~[uniprot]_Ribosomal_protein_L11_methyltransferase~~~[uniprot]_UvrABC_system_protein_A~~~[uniprot]_Dihydroorotase~~~[uniprot]_tRNA1(Val)_(adenine(37)-N6)-methyltransferase~~~[uniprot]_Protein-L-isoaspartate_O-methyltransferase~~~[uniprot]_Uncharacterized_protein_Mb1438c~~~[uniprot]_Uncharacterized_protein_Rv1403c~~~[uniprot]_Uncharacterized_protein_MT1447~~~[uniprot]_N-acetyl-gamma-glutamyl-phosphate_reductase~~~[uniprot]_Ribosomal_RNA_large_subunit_methyltransferase_E
gid	136	n358132	236	232/281 (82.6)	4/56 (7.1)	Yes	7.94256E-09	-0.4931431	12.03585938	TATGGGACCGGCATCTACTGAACTGCGCCGTGATCGGTGAGCTCCTCGAACGCGG	55	(AGly)GIDB~~~[uniprot]_Ribosomal_RNA_small_subunit_methyltransferase_G
gid	136	n407225	235	231/281 (82.2)	4/56 (7.1)	Yes	3.0005E-08	-0.4857804	11.73377507	TCGTGGATATCGGTAGCGGAGCCGGGTTGCC	31	(AGly)GIDB
gid	136	n438722	233	229/281 (81.5)	4/56 (7.1)	Yes	4.13436E-07	-0.469309	10.79347418	TCTAGGAGAACTACCTGGAGGTCCGGCCGCGCTATCGCC	39	(AGly)GIDB~~~[uniprot]_Ribosomal_RNA_small_subunit_methyltransferase_G
emrB	102	n446406	101	49/281 (17.4)	52/56 (92.9)	Yes	7.94256E-09	0.4979022	14.19291042	CACCTACCAGTTCAATCACAGCGAAATCATAGCTACTGCAAAGAAAGTCGCACTGACCCCA	61	[uniprot]_Multidrug_resistance_protein_B_homolog~~~[uniprot]_Biotin_sulfoxide_reductase
emrB	102	n446407	234	230/281 (81.9)	4/56 (7.1)	Yes	1.14492E-07	-0.4841166	12.58056117	CACCTACCAGTTCAATCACAGCGAAATCATCGCTACTGCAAAGAAAGTCGCACTGACC	58	[uniprot]_Multidrug_resistance_protein_B_homolog
pncA	137	n121112	65	23/281 (8.2)	42/56 (75.0)	Yes	7.63904E-12	0.4928669	9.852821467	GCCGAGGACGCGGTACGCAATGGCTTGGCCCACCAGGGTGCTGGTGGACCTGACAGCGG	59	(MTB)PNCA~~~[uniprot]_5\'-nucleotidase_SurE

Supplementary Table S3: Single nucleotide variants and small indels that were significantly associated with different phenotypes in Hanoi's and KwaZulu-Natal's study population

Phenotypes						Positions with SNVs*	No of SNVs
**cluster[+] Hanoi**	**S315T[+] Hanoi**	**cluster[+]/ S315T[+] Hanoi**	**cluster[+] KwaZulu-Natal**	**S315T[+] KwaZulu-Natal**	**cluster[+]/ S315T[+] KwaZulu-Natal**		
No	No	**Yes**	No	No	No	897358, 2562288, 786311, 2056866, 3160421, 3778338, 567208, 2191082, 783342, 3640321, 2876955, 1027239, 3710380, 1954655, 2403177, 179091, 2129998, 2423422, 3594831, 3046181, 1740691, 2701529, 1598423, 526058, 2766789, 1437948, 542074, 1908105, 3782763, 1619591, 2438670, 3177276, 219804, 2775156, 499816, 3099391, 4204224, 1436403, 1600063, 3784399, 4277325, 2449325, 1419193, 2774856, 211664, 2056902, 1574837, 1735805, 3001584, 1934875, 1340667, 3944342, 1091590, 3944340, 2803670, 1339649, 631318, 3607420, 1339644, 3859614, 1893183, 1068960, 4018615, 4258229, 3368380, 3583543, 3734943, 2720993, 226522, 2876938, 3257937, 3963393, 2595142, 3794515, 1789735, 2586925, 4117974, 861878, 480468, 3405338, 3872538, 3057998, 2502324, 2757836, 2984903, 192182, 2441637, 1659662, 3142701, 3298532, 848436, 3766200, 2142602, 767570, 2032791, 218465, 343185, 1677986, 275657, 2867088, 1167975, 2947988, 3304104, 1630688, 3197045, 2211210, 1731055, 60414, 1171094, 2859133, 1846481, 2964867, 479505, 3443266, 1497267, 64495, 3538141, 1690715, 2326293, 4015697, 1355645, 4338537, 1545881, 2344942, 842266, 2208493, 876918, 4382055, 1290838, 1394032, 1942636, 4143453, 3989051, 3451440, 94961, 2212028, 1966742, 544146, 61846, 1641019, 581848, 795920, 1166498, 3089878, 2318825, 1099142, 4067394, 2226037, 1341344, 3575931, 3430006, 209259, 3042024, 2842880, 1846335, 1112922, 2453147, 4130795, 1145941, 1304703, 4314255, 975932, 4161437, 1309585, 3725794, 1340688, 1099769, 3949345, 2288820, 1216545, 83778, 3604064, 767395, 845386, 3941595, 3893944, 3746219, 777664, 3941605, 1411346, 3941604, 2081011, 927496, 2628895, 1987546, 1385214, 626195, 1423583, 1233342, 2840109, 2193886, 2605885, 2888827, 194787, 3451186, 3213678, 3866167, 346506, 2408079, 4407686, 1973987, 3545427, 4117356, 4228988, 1275379, 1358237, 2733749, 350702, 2044116, 3914154, 4369452, 3819448, 2677876, 14270, 3936503, 3007119, 4343176, 4381079, 433627, 3141737, 2044884, 2662802, 2284326, 1405701, 3818638, 952487, 192492, 1753350, 1690284, 1358515, 179874, 2614232, 548497, 3365184, 2980684, 4285042, 4024520, 508326, 430177, 2078585, 3437020, 2383160, 684355, 257071, 3079112, 840205, 840206, 840207, 2427457, 1086711, 2263631, 161416, 2628893, 3736108, 1800902, 3844733, 3218997, 782148, 3000401, 2318206, 312686, 1760688, 549777, 2500964, 1160131, 1194351, 3068497, 782634, 450525, 3899672, 2835894, 215049, 132596, 224057, 2251313, 489741, 3721174, 2714526, 217459, 1164619, 993352, 2255482, 3770743, 4058762, 2043141, 2220482, 1675193, 150496, 1202643, 845697, 3287430, 3736828, 334160, 348095, 2138730, 725365, 256818, 2358525, 141457, 308520, 1886155, 3540882, 3684649, 292679, 550103, 3300005, 513789, 335663, 4076016, 449633, 1554418, 3462822, 3570896, 845990, 2284652, 1241386, 1232778, 2610381, 2739546, 1651706, 1397606, 3724042, 655559, 774065, 3193202, 2093991, 826717, 396771, 1341152, 1843620, 115267, 3413574, 1341148	334
No	No	No	Yes	No	Yes	763123, 2246032, 664929, 4056430, 4269271, 761110, 756757, 1272321, 2288785, 2300669, 2300674, 2300676, 2300678, 2300680, 2300690, 2300699, 3889150, 7570, 2300666, 1673432, 761161, 1473246, 762889	23
No	No	No	No	No	Yes	4354671, 2601713, 2608956, 3633957, 302691, 537387, 881918, 1370737, 2665148, 3533193, 3296639, 3856224, 4011950, 1967844, 1264381, 877058, 147946, 572468, 1197700, 2486496	20
No	No	No	Yes	Yes	Yes	24125, 122107, 2401402, 3272997, 4327484, 4409995	6
No	No	No	Yes	No	No	2195976, 2535716, 849742	3
No	Yes	No	No	No	No	4374724, 4063178	2
No	Yes	**Yes**	No	Yes	No	2155168	1
No	No	No	No	Yes	No	781822	1

cluster[+]: clustered (pairwise SNV difference between the two isolates is no more than five SNVs); S315T[+]: harboring *katG*-S315T mutation; Hanoi: Hanoi's study cohort; KwaZulu-Natal: KwaZulu-Natal's study cohort; SNV: single nucleotide variant

*3944342: two SNVs were reported at one position.

Supplemetary Table S4: Details of single nucleotide variants and small indels that were significantly associated with strains carrying *katG*-S315T mutation in the Hanoi study population.

Position	Gene	Locus name	Pattern	P value	Principal component	Variant category	Impact	Nucleotide change	Amin acid change	No among phenotype (-)	No among phenotype (+)
14270	*Rv0012*	Rv0012	1227	2.99092E-06	10	missense_variant	MODERATE	c.182A>G	p.Asp61Gly	7	4
60414	*Rv0057*	Rv0057	1017	1.50744E-08	7	synonymous_variant	LOW	c.519C>T	p.Thr173Thr	10	6
61846	*dnaB*	Rv0058	1017	1.50744E-08	7	missense_variant	MODERATE	c.1451C>A	p.Ala484Glu	10	6
64495	*Rv0060*	Rv0060	1017	1.50744E-08	7	missense_variant	MODERATE	c.587A>G	p.His196Arg	10	6
83778	*Rv0074*	Rv0074	67	5.28411E-07	324	missense_variant	MODERATE	c.1031C>T	p.Thr344Ile	0	1
94961	*hycQ*	Rv0086	1017	1.50744E-08	7	synonymous_variant	LOW	c.1011C>T	p.Leu337Leu	10	6
115267	*nrp*	Rv0101	1231	1.71959E-05	10	missense_variant	MODERATE	c.5267T>C	p.Val1756Ala	8	4
132596	*PE_PGRS1*	Rv0109	1231	1.71959E-05	10	conservative_inframe_deletion	MODERATE	c.1231_1239delGCCGGCGGG	p.Ala411_Gly413del	8	4
141457	*oxyS*	Rv0117	1231	1.71959E-05	10	synonymous_variant	LOW	c.258G>C	p.Arg86Arg	8	4
150496	*PE_PGRS2*	Rv0124	1231	1.71959E-05	10	missense_variant	MODERATE	c.964A>G	p.Ile322Val	8	4
161416	*Rv0133*	Rv0133	666	0.000009805	217	missense_variant	MODERATE	c.548A>G	p.Glu183Gly	0	1
179091	*PE1*	Rv0151c	391	7.33622E-13	15	synonymous_variant	LOW	c.219G>T	p.Pro73Pro	0	2
179874	*PE2*	Rv0152c	1019	6.34814E-06	7	synonymous_variant	LOW	c.1023C>T	p.Gly341Gly	14	6
192182	*adhE1*	Rv0162c	1017	1.50744E-08	7	synonymous_variant	LOW	c.954A>G	p.Ser318Ser	10	6
192492	*adhE1*	Rv0162c	1019	6.34814E-06	7	missense_variant	MODERATE	c.644G>A	p.Gly215Glu	14	6
194787	*mce1R*	Rv0165c	1227	2.99092E-06	10	missense_variant	MODERATE	c.29G>A	p.Arg10Gln	7	4
209259	*Rv0178*	Rv0178	1017	1.50744E-08	7	missense_variant	MODERATE	c.322C>T	p.Arg108Cys	10	6
211664	*Rv0180c*	Rv0180c	617	1.59682E-09	215	missense_variant	MODERATE	c.587G>C	p.Ser196Thr	0	2
215049	*Rv0184*	Rv0184	1231	1.71959E-05	10	synonymous_variant	LOW	c.81C>T	p.Asp27Asp	8	4
217459	*bglS*	Rv0186	1231	1.71959E-05	10	synonymous_variant	LOW	c.1191T>C	p.Ser397Ser	8	4
218465	*mymT*	Rv0186A	1017	1.50744E-08	7	synonymous_variant	LOW	c.87A>G	p.Glu29Glu	10	6
219804	*Rv0188*	Rv0188	470	1.55984E-09	216	missense_variant	MODERATE	c.319G>A	p.Gly107Arg	0	2
224057	*Rv0192*	Rv0192	1231	1.71959E-05	10	missense_variant	MODERATE	c.494C>T	p.Ala165Val	8	4
226522	*Rv0193c*	Rv0193c	1017	1.50744E-08	7	missense_variant	MODERATE	c.50C>T	p.Thr17Ile	10	6
256818	*fadD4*	Rv0214	1231	1.71959E-05	10	missense_variant	MODERATE	c.755C>T	p.Ala252Val	8	4
257071	*fadD4*	Rv0214	1019	6.34814E-06	7	synonymous_variant	LOW	c.1008C>T	p.Tyr336Tyr	14	6
275657	*php*	Rv0230c	1017	1.50744E-08	7	missense_variant	MODERATE	c.307G>C	p.Val103Leu	10	6
292679	*fadA2*	Rv0243	1231	1.71959E-05	10	missense_variant	MODERATE	c.509A>G	p.Glu170Gly	8	4
308520	*PPE2*	Rv0256c	1231	1.71959E-05	10	missense_variant	MODERATE	c.1028C>A	p.Ala343Asp	8	4
312686	*cobQ1*	Rv0255c	1231	1.71959E-05	10	upstream_gene_variant	MODIFIER	c.-4828G>A		8	4
334160	*PE_PGRS3*	Rv0278c	1231	1.71959E-05	10	synonymous_variant	LOW	c.2151G>T	p.Leu717Leu	8	4
335663	*PE_PGRS3*	Rv0278c	1231	1.71959E-05	10	disruptive_inframe_deletion	MODERATE	c.606_647delCGCCGGCGGCGCCGGCGGCGGCGTAGTAGCCCTCACGGGCGG	p.Ala203_Gly216del	8	4
343185	*eccA3*	Rv0282	1017	1.50744E-08	7	synonymous_variant	LOW	c.1056C>T	p.Arg352Arg	10	6
346506	*eccC3*	Rv0284	1227	2.99092E-06	10	missense_variant	MODERATE	c.872C>T	p.Ala291Val	7	4
348095	*eccC3*	Rv0284	1231	1.71959E-05	10	missense_variant	MODERATE	c.2461T>C	p.Ser821Pro	8	4
350702	*PPE4*	Rv0286	1227	2.99092E-06	10	synonymous_variant	LOW	c.768C>T	p.Gly256Gly	7	4
396771	*Rv0331*	Rv0331	1231	1.71959E-05	10	missense_variant	MODERATE	c.571C>G	p.Pro191Ala	8	4
430177	*PPE8*	Rv0355c	1019	6.34814E-06	7	frameshift_variant	HIGH	c.4502dupA	p.Asn1501fs	14	6
433627	*PPE8*	Rv0355c	1019	6.34814E-06	7	synonymous_variant	LOW	c.1053C>T	p.Ser351Ser	14	6
449633	*Rv0373c*	Rv0373c	1231	1.71959E-05	10	missense_variant	MODERATE	c.2171C>T	p.Thr724Ile	8	4
450525	*Rv0373c*	Rv0373c	1231	1.71959E-05	10	missense_variant	MODERATE	c.1279A>C	p.Thr427Pro	8	4
479505	*fadE7*	Rv0400c	1017	1.50744E-08	7	synonymous_variant	LOW	c.249G>T	p.Ala83Ala	10	6
480468	*mmpL1*	Rv0402c	1017	1.50744E-08	7	missense_variant	MODERATE	c.2764G>C	p.Gly922Arg	10	6
489741	*pks6*	Rv0405	1231	1.71959E-05	10	synonymous_variant	LOW	c.4011G>A	p.Val1337Val	8	4

499816	*mutT3*	Rv0413	470	1.55984E-09	216	missense_variant	MODERATE	c.104C>T	p.Ala35Val	0	2
508326	*thiD*	Rv0422c	1019	6.34814E-06	7	missense_variant	MODERATE	c.230A>G	p.Lys77Arg	14	6
513789	*ctpH*	Rv0425c	1231	1.71959E-05	10	synonymous_variant	LOW	c.1533G>A	p.Val511Val	8	4
526058	*psd*	Rv0437c	667	7.17007E-11	217	missense_variant	MODERATE	c.25G>C	p.Gly9Arg	0	2
542074	*Rv0448c*	Rv0448c	667	7.17007E-11	217	upstream_gene_variant	MODIFIER	c.-4905T>A		0	2
544146	*PPE11*	Rv0453	1017	1.50744E-08	7	missense_variant	MODERATE	c.973G>A	p.Val325Ile	10	6
548497	*Rv0457c*	Rv0457c	1019	6.34814E-06	7	missense_variant	MODERATE	c.1111G>A	p.Ala371Thr	14	6
549777	*Rv0458*	Rv0458	1231	1.71959E-05	10	missense_variant	MODERATE	c.103C>T	p.Arg35Cys	8	4
550103	*Rv0458*	Rv0458	1231	1.71959E-05	10	synonymous_variant	LOW	c.429C>T	p.Ile143Ile	8	4
567208	*Rv0477*	Rv0477	391	7.33622E-13	15	missense_variant	MODERATE	c.433G>A	p.Glu145Lys	0	2
581848	*Rv0492c*	Rv0492c	1017	1.50744E-08	7	missense_variant	MODERATE	c.1531G>A	p.Val511Ile	10	6
626195	*menA*	Rv0534c	1227	2.99092E-06	10	synonymous_variant	LOW	c.246G>A	p.Arg82Arg	7	4
631318	*Rv0538*	Rv0538	1016	1.94095E-09	7	missense_variant	MODERATE	c.1279C>A	p.Pro427Thr	9	6
655559	*gpdA1*	Rv0564c	1231	1.71959E-05	10	missense_variant	MODERATE	c.391C>T	p.Pro131Ser	8	4
684355	*Rv0585c*	Rv0585c	1231	1.71959E-05	10	upstream_gene_variant	MODIFIER	c.-84delC		8	4
725365	*recC*	Rv0631c	1231	1.71959E-05	10	missense_variant	MODERATE	c.2942T>C	p.Phe981Ser	8	4
767395	*end*	Rv0670	67	5.28411E-07	324	upstream_gene_variant	MODIFIER	c.-2397G>A		0	1
767570	*end*	Rv0670	1017	1.50744E-08	7	upstream_gene_variant	MODIFIER	c.-2222G>A		10	6
774065	*Rv0674*	Rv0674	1231	1.71959E-05	10	start_lost	HIGH	c.2T>C	p.Met1?	8	4
777664	*mmpL5*	Rv0676c	631	0.00000078	281	missense_variant	MODERATE	c.817G>A	p.Ala273Thr	0	1
782148	*rpsG*	Rv0683	1231	1.71959E-05	10	missense_variant	MODERATE	c.215C>A	p.Ala72Asp	8	4
782634	*fusA1*	Rv0684	1231	1.71959E-05	10	synonymous_variant	LOW	c.150A>G	p.Ala50Ala	8	4
783342	*fusA1*	Rv0684	391	7.33622E-13	15	synonymous_variant	LOW	c.858G>A	p.Pro286Pro	0	2
786311	*Rv0686*	Rv0686	381	1.75432E-13	299	missense_variant	MODERATE	c.163C>T	p.Leu55Phe	0	2
795920	*Rv0696*	Rv0696	1017	1.50744E-08	7	synonymous_variant	LOW	c.402A>G	p.Val134Val	10	6
826717	*mapA*	Rv0734	1231	1.71959E-05	10	synonymous_variant	LOW	c.48C>T	p.Arg16Arg	8	4
840205	*PE_PGRS10*	Rv0747	123	9.38491E-06	217	frameshift_variant	HIGH	c.1755_1756insGG	p.Leu586fs	0	1
840206	*PE_PGRS10*	Rv0747	123	9.38491E-06	217	frameshift_variant	HIGH	c.1756_1757insG	p.Leu586fs	0	1
840207	*PE_PGRS10*	Rv0747	123	9.38491E-06	217	missense_variant	MODERATE	c.1757T>G	p.Leu586Arg	0	1
842266	*Rv0750*	Rv0750	1017	1.50744E-08	7	synonymous_variant	LOW	c.234C>T	p.Ala78Ala	10	6
845386	*mmsA*	Rv0753c	631	0.00000078	281	missense_variant	MODERATE	c.568T>C	p.Phe190Leu	0	1
845697	*mmsA*	Rv0753c	1231	1.71959E-05	10	missense_variant	MODERATE	c.257A>C	p.Glu86Ala	8	4
845990	*Rv0749A*	Rv0749A	1231	1.71959E-05	10	upstream_gene_variant	MODIFIER	c.-4122_-4117delAGTATC		8	4
848436	*PPE12*	Rv0755c	1017	1.50744E-08	7	synonymous_variant	LOW	c.1605T>C	p.Ser535Ser	10	6
861878	*aldA*	Rv0768	1017	1.50744E-08	7	missense_variant	MODERATE	c.967G>A	p.Gly323Ser	10	6
876918	*emrB*	Rv0783c	1017	1.50744E-08	7	missense_variant	MODERATE	c.1523T>C	p.Phe508Ser	10	6
897358	*purL*	Rv0803	1017	1.50744E-08	7	synonymous_variant	LOW	c.1539C>T	p.Cys513Cys	10	6
927496	*PE_PGRS13*	Rv0833	471	0.000001085	40	disruptive_inframe_deletion	MODERATE	c.2151_2159delCGGCAACGG	p.Gly718_Gly720del	1	2
952487	*far*	Rv0855	1019	6.34814E-06	7	missense_variant	MODERATE	c.856C>T	p.Arg286Cys	14	6
975932	*Rv0876c*	Rv0876c	1017	1.50744E-08	7	synonymous_variant	LOW	c.6C>T	p.Ala2Ala	10	6
993352	*Rv0891c*	Rv0891c	1231	1.71959E-05	10	missense_variant	MODERATE	c.104C>T	p.Ala35Val	8	4
1027239	*Rv0921*	Rv0921	391	7.33622E-13	15	missense_variant	MODERATE	c.136T>G	p.Ser46Ala	0	2
1068960	*purH*	Rv0957	1017	1.50744E-08	7	synonymous_variant	LOW	c.756C>T	p.His252His	10	6
1086711	*accD2*	Rv0974c	666	0.000009805	217	missense_variant	MODERATE	c.635T>C	p.Val212Ala	0	1
1091590	*PE_PGRS16*	Rv0977	893	1.94095E-09	7	conservative_inframe_deletion	MODERATE	c.1231_1233delGGC	p.Gly411del	9	6
1099142	*pepD*	Rv0983	1017	1.50744E-08	7	missense_variant	MODERATE	c.77C>T	p.Ser26Leu	10	6
1099769	*pepD*	Rv0983	1018	9.0534E-08	7	missense_variant	MODERATE	c.704G>A	p.Ser235Asn	11	6
1112922	*Rv0996*	Rv0996	1017	1.50744E-08	7	disruptive_inframe_deletion	MODERATE	c.560_565delAATACG	p.Glu187_Tyr188del	10	6

1145941	*Rv1024*	Rv1024	1017	1.50744E-08	7	synonymous_variant	LOW	c.84T>C	p.Gly28Gly	10	6
1160131	*Rv1036c*	Rv1036c	1231	1.71959E-05	10	synonymous_variant	LOW	c.303G>A	p.Glu101Glu	8	4
1164619	*Rv1041c*	Rv1041c	1231	1.71959E-05	10	missense_variant	MODERATE	c.817G>A	p.Val273Ile	8	4
1166498	*Rv1043c*	Rv1043c	1017	1.50744E-08	7	frameshift_variant	HIGH	c.308delG	p.Gly103fs	10	6
1167975	*Rv1045*	Rv1045	1017	1.50744E-08	7	synonymous_variant	LOW	c.303G>A	p.Glu101Glu	10	6
1171094	*Rv1048c*	Rv1048c	1017	1.50744E-08	7	missense_variant	MODERATE	c.1060G>A	p.Ala354Thr	10	6
1194351	*echA8*	Rv1070c	1231	1.71959E-05	10	synonymous_variant	LOW	c.693T>C	p.His231His	8	4
1202643	*cbs*	Rv1077	1231	1.71959E-05	10	synonymous_variant	LOW	c.927G>T	p.Ala309Ala	8	4
1216545	*PE_PGRS22*	Rv1091	380	0.000000498	324	missense_variant	MODERATE	c.77C>T	p.Ala26Val	0	1
1232778	*Rv1105*	Rv1105	1231	1.71959E-05	10	frameshift_variant	HIGH	c.470_474delACGAC	p.His157fs	8	4
1233342	*Rv1106c*	Rv1106c	1227	2.99092E-06	10	synonymous_variant	LOW	c.615C>G	p.Leu205Leu	7	4
1241386	*Rv1116A*	Rv1116A	1231	1.71959E-05	10	missense_variant	MODERATE	c.5G>A	p.Gly2Asp	8	4
1275379	*Rv1147*	Rv1147	1227	2.99092E-06	10	synonymous_variant	LOW	c.480C>T	p.Asp160Asp	7	4
1290838	*narG*	Rv1161	1017	1.50744E-08	7	missense_variant	MODERATE	c.3511C>T	p.Pro1171Ser	10	6
1304703	*fbiC*	Rv1173	1017	1.50744E-08	7	synonymous_variant	LOW	c.1773C>T	p.Ala591Ala	10	6
1309585	*Rv1178*	Rv1178	1017	1.50744E-08	7	synonymous_variant	LOW	c.222T>C	p.Ala74Ala	10	6
1339644	*PPE18*	Rv1196	1016	1.94095E-09	7	missense_variant	MODERATE	c.296A>C	p.Glu99Ala	9	6
1339649	*PPE18*	Rv1196	1016	1.94095E-09	7	missense_variant	MODERATE	c.301G>A	p.Ala101Thr	9	6
1340667	*esxK*	Rv1197	1277	1.77973E-09	7	synonymous_variant	LOW	c.9A>G	p.Ser3Ser	29	10
1340688	*esxK*	Rv1197	894	8.81145E-08	7	synonymous_variant	LOW	c.30C>T	p.His10His	11	6
1341148	*esxL*	Rv1198	1020	0.000019787	7	missense_variant	MODERATE	c.143C>T	p.Ala48Val	15	6
1341152	*esxL*	Rv1198	1020	0.000019787	7	synonymous_variant	LOW	c.147C>T	p.Ala49Ala	15	6
1341344	*Rv1194c*	Rv1194c	1017	1.50744E-08	7	upstream_gene_variant	MODIFIER	c.-2831G>A		10	6
1355645	*glgA*	Rv1212c	1017	1.50744E-08	7	missense_variant	MODERATE	c.17T>C	p.Leu6Ser	10	6
1358237	*Rv1215c*	Rv1215c	1227	2.99092E-06	10	missense_variant	MODERATE	c.1208C>T	p.Pro403Leu	7	4
1358515	*Rv1215c*	Rv1215c	1019	6.34814E-06	7	missense_variant	MODERATE	c.930G>T	p.Glu310Asp	14	6
1385214	*PE_PGRS23*	Rv1243c	896	1.79921E-06	7	synonymous_variant	LOW	c.1464C>T	p.Ala488Ala	13	6
1394032	*relB*	Rv1247c	1017	1.50744E-08	7	upstream_gene_variant	MODIFIER	c.-4788G>A		10	6
1397606	*Rv1251c*	Rv1251c	1231	1.71959E-05	10	synonymous_variant	LOW	c.1635G>A	p.Glu545Glu	8	4
1405701	*Rv1257c*	Rv1257c	1019	6.34814E-06	7	synonymous_variant	LOW	c.384T>C	p.Tyr128Tyr	14	6
1411346	*amiB2*	Rv1263	1019	6.34814E-06	7	synonymous_variant	LOW	c.916C>T	p.Leu306Leu	14	6
1419193	*lprA*	Rv1270c	617	1.59682E-09	215	missense_variant	MODERATE	c.556A>C	p.Thr186Pro	0	2
1423583	*Rv1273c*	Rv1273c	1227	2.99092E-06	10	synonymous_variant	LOW	c.468G>A	p.Ala156Ala	7	4
1436403	*oppB*	Rv1283c	617	1.59682E-09	215	missense_variant	MODERATE	c.715C>T	p.Leu239Phe	0	2
1437948	*cysD*	Rv1285	667	7.17007E-11	217	missense_variant	MODERATE	c.40A>C	p.Ile14Leu	0	2
1497267	*dinG*	Rv1329c	1017	1.50744E-08	7	missense_variant	MODERATE	c.1923G>C	p.Trp641Cys	10	6
1545881	*Rv1372*	Rv1372	1017	1.50744E-08	7	missense_variant	MODERATE	c.1057A>G	p.Ile353Val	10	6
1554418	*pyrC*	Rv1381	1231	1.71959E-05	10	synonymous_variant	LOW	c.231G>A	p.Ala77Ala	8	4
1574837	*cyp132*	Rv1394c	617	1.59682E-09	215	upstream_gene_variant	MODIFIER	c.-3868G>A		0	2
1598423	*whiA*	Rv1423	667	7.17007E-11	217	missense_variant	MODERATE	c.518C>A	p.Ala173Asp	0	2
1600063	*Rv1425*	Rv1425	617	1.59682E-09	215	missense_variant	MODERATE	c.406A>C	p.Met136Leu	0	2
1619591	*PE_PGRS26*	Rv1441c	667	7.17007E-11	217	missense_variant	MODERATE	c.94G>A	p.Ala32Thr	0	2
1630688	*PE_PGRS27*	Rv1450c	1017	1.50744E-08	7	missense_variant	MODERATE	c.3940G>A	p.Ala1314Thr	10	6
1641019	*Rv1455*	Rv1455	631	0.00000078	281	missense_variant	MODERATE	c.340G>C	p.Val114Leu	0	1
1651706	*csd*	Rv1464	1231	1.71959E-05	10	synonymous_variant	LOW	c.189C>T	p.Gly63Gly	8	4
1659662	*trxB1*	Rv1471	1017	1.50744E-08	7	missense_variant	MODERATE	c.293G>A	p.Ser98Asn	10	6
1675193	*hemZ*	Rv1485	1231	1.71959E-05	10	synonymous_variant	LOW	c.177C>G	p.Arg59Arg	8	4
1677986	*Rv1488*	Rv1488	1017	1.50744E-08	7	missense_variant	MODERATE	c.590G>A	p.Gly197Asp	10	6
1690284	*Rv1498A*	Rv1498A	1019	6.34814E-06	7	synonymous_variant	LOW	c.63G>A	p.Ala21Ala	14	6
1690715	*Rv1499*	Rv1499	1017	1.50744E-08	7	synonymous_variant	LOW	c.309C>A	p.Leu103Leu	10	6
1731055	*fadD24*	Rv1529	1017	1.50744E-08	7	synonymous_variant	LOW	c.1554C>T	p.Asp518Asp	10	6

1735805	*Rv1532c*	Rv1532c	617	1.59682E-09	215	upstream_gene_variant	MODIFIER	c.-2255C>T		0	2
1740691	*dinX*	Rv1537	667	7.17007E-11	217	missense_variant	MODERATE	c.836G>A	p.Gly279Asp	0	2
1753350	*PPE21*	Rv1548c	1019	6.34814E-06	7	upstream_gene_variant	MODIFIER	c.-22_-18delGCCGG		14	6
1760688	*frdD*	Rv1555	1231	1.71959E-05	10	missense_variant	MODERATE	c.137C>G	p.Pro46Arg	8	4
1789735	*Rv1588c*	Rv1588c	1017	1.50744E-08	7	synonymous_variant	LOW	c.102G>A	p.Pro34Pro	10	6
1800902	*hisC1*	Rv1600	1013	1.46315E-05	7	synonymous_variant	LOW	c.7A>C	p.Arg3Arg	1	2
1843620	*Rv1637c*	Rv1637c	1231	1.71959E-05	10	missense_variant	MODERATE	c.73A>G	p.Asn25Asp	8	4
1846335	*uvrA*	Rv1638	1017	1.50744E-08	7	synonymous_variant	LOW	c.2595C>G	p.Thr865Thr	10	6
1846481	*uvrA*	Rv1638	1017	1.50744E-08	7	missense_variant	MODERATE	c.2741T>C	p.Ile914Thr	10	6
1886155	*pks8*	Rv1662	1231	1.71959E-05	10	synonymous_variant	LOW	c.4452G>T	p.Leu1484Leu	8	4
1893183	*cyp139*	Rv1666c	727	6.17792E-09	207	missense_variant	MODERATE	c.380G>A	p.Ser127Asn	1	2
1908105	*Rv1683*	Rv1683	667	7.17007E-11	217	missense_variant	MODERATE	c.512T>C	p.Val171Ala	0	2
1934875	*cycA*	Rv1704c	617	1.59682E-09	215	upstream_gene_variant	MODIFIER	c.-3419A>G		0	2
1942636	*Rv1714*	Rv1714	1017	1.50744E-08	7	missense_variant	MODERATE	c.784T>C	p.Tyr262His	10	6
1954655	*Rv1729c*	Rv1729c	391	7.33622E-13	15	synonymous_variant	LOW	c.915G>T	p.Pro305Pro	0	2
1966742	*Rv1739c*	Rv1739c	1017	1.50744E-08	7	missense_variant	MODERATE	c.896G>C	p.Cys299Ser	10	6
1973987	*Rv1747*	Rv1747	1227	2.99092E-06	10	missense_variant	MODERATE	c.358C>T	p.Arg120Cys	7	4
1987546	*plcD*	Rv1755c	390	1.30344E-06	331	frameshift_variant&stop gained	HIGH	c.150_151insTCGATGAACCAC CTGACATGACCCCATCCTTT CCAAGAACTGGAGTCTCCG GACATGCCGGGGCGGTTCA	p.Ala51fs	0	1
2032791	*eccD5*	Rv1795	1017	1.50744E-08	7	synonymous_variant	LOW	c.552C>T	p.Val184Val	10	6
2043141	*PPE29*	Rv1801	1231	1.71959E-05	10	missense_variant	MODERATE	c.1141C>T	p.Arg381Cys	8	4
2044116	*PPE30*	Rv1802	1227	2.99092E-06	10	missense_variant	MODERATE	c.733G>A	p.Glu245Lys	7	4
2044884	*PE20*	Rv1806	1019	6.34814E-06	7	upstream_gene_variant	MODIFIER	c.-3188C>A		14	6
2056866	*erg3*	Rv1814	391	7.33622E-13	15	missense_variant	MODERATE	c.346C>T	p.Arg116Trp	0	2
2056902	*erg3*	Rv1814	617	1.59682E-09	215	missense_variant	MODERATE	c.382A>G	p.Ser128Gly	0	2
2078585	*gcvB*	Rv1832	1019	6.34814E-06	7	synonymous_variant	LOW	c.2709C>T	p.Ala903Ala	14	6
2081011	*Rv1835c*	Rv1835c	631	0.00000078	281	frameshift_variant	HIGH	c.1576_1577insATACA	p.Ile526fs	0	1
2093991	*gnd1*	Rv1844c	1231	1.71959E-05	10	missense_variant	MODERATE	c.1198G>A	p.Ala400Thr	8	4
2129998	*Rv1879*	Rv1879	391	7.33622E-13	15	stop_gained	HIGH	c.622C>T	p.Arg208*	0	2
2138730	*Rv1889c*	Rv1889c	1231	1.71959E-05	10	missense_variant	MODERATE	c.288C>A	p.Asp96Glu	8	4
2142602	*Rv1895*	Rv1895	1017	1.50744E-08	7	missense_variant	MODERATE	c.82G>A	p.Val28Met	10	6
2155168	*katG*	Rv1908c	1776	0.000000005	15	missense_variant	MODERATE	c.944G>C	p.Ser315Thr	72	15
2191082	*ephB*	Rv1938	391	7.33622E-13	15	missense_variant	MODERATE	c.56C>T	p.Ala19Val	0	2
2193886	*Rv1941*	Rv1941	1227	2.99092E-06	10	missense_variant	MODERATE	c.223G>A	p.Ala75Thr	7	4
2208493	*yrbE3A*	Rv1964	1017	1.50744E-08	7	missense_variant	MODERATE	c.794C>T	p.Ser265Phe	10	6
2211210	*mce3B*	Rv1967	1017	1.50744E-08	7	missense_variant	MODERATE	c.610A>G	p.Asn204Asp	10	6
2212028	*mce3C*	Rv1968	1017	1.50744E-08	7	missense_variant	MODERATE	c.403C>T	p.Pro135Ser	10	6
2220482	*Rv1977*	Rv1977	1231	1.71959E-05	10	frameshift_variant	HIGH	c.731delA	p.Asn244fs	8	4
2226037	*vapB36*	Rv1982A	1017	1.50744E-08	7	missense_variant	MODERATE	c.65A>G	p.His22Arg	10	6
2251313	*Rv2005c*	Rv2005c	1231	1.71959E-05	10	missense_variant	MODERATE	c.571A>G	p.Ile191Val	8	4
2255482	*otsB1*	Rv2006	1231	1.71959E-05	10	missense_variant	MODERATE	c.3481G>A	p.Gly1161Ser	8	4
2263631	*Rv2016*	Rv2016	666	0.000009805	217	frameshift_variant&stop gained	HIGH	c.206_207insCCGCCCCGGTG AGTCCGGAGACTCTCTGATC TGAGACCTCAGCCGGCGGC TGGTCTCT	p.Lys69fs	0	1
2284326	*Rv2038c*	Rv2038c	1019	6.34814E-06	7	synonymous_variant	LOW	c.471C>T	p.Phe157Phe	14	6
2284652	*Rv2038c*	Rv2038c	1231	1.71959E-05	10	synonymous_variant	LOW	c.145C>A	p.Arg49Arg	8	4
2288820	*pncA*	Rv2043c	668	4.91945E-07	217	missense_variant	MODERATE	c.422A>C	p.Gln141Pro	1	2

2318206	*cobN*	Rv2062c	1231	1.71959E-05	10	missense_variant	MODERATE	c.2548T>G	p.Trp850Gly	8	4
2318825	*cobN*	Rv2062c	1017	1.50744E-08	7	synonymous_variant	LOW	c.1929C>T	p.Asp643Asp	10	6
2326293	*blaC*	Rv2068c	1017	1.50744E-08	7	missense_variant	MODERATE	c.517G>C	p.Asp173His	10	6
2344942	*pknJ*	Rv2088	1017	1.50744E-08	7	missense_variant	MODERATE	c.532G>A	p.Ala178Thr	10	6
2358525	*Rv2100*	Rv2100	1231	1.71959E-05	10	missense_variant	MODERATE	c.137A>C	p.Asp46Ala	8	4
2383160	*metH*	Rv2124c	1019	6.34814E-06	7	missense_variant	MODERATE	c.2908G>A	p.Gly970Ser	14	6
2403177	*Rv2143*	Rv2143	391	7.33622E-13	15	stop_gained	HIGH	c.201G>A	p.Trp67*	0	2
2408079	*yfiH*	Rv2149c	1227	2.99092E-06	10	missense_variant	MODERATE	c.296T>C	p.Val99Ala	7	4
2423422	*PE_PGRS38*	Rv2162c	391	7.33622E-13	15	conservative_inframe_deletion	MODERATE	c.1309_1416del	p.Asn437_Gly472del	0	2
2427457	*Rv2164c*	Rv2164c	666	0.000009805	217	missense_variant	MODERATE	c.782C>T	p.Pro261Leu	0	1
2438670	*pknL*	Rv2176	470	1.55984E-09	216	missense_variant	MODERATE	c.730G>A	p.Gly244Ser	0	2
2441637	*aroG*	Rv2178c	1017	1.50744E-08	7	synonymous_variant	LOW	c.84C>G	p.Ala28Ala	10	6
2449325	*fadD15*	Rv2187	617	1.59682E-09	215	missense_variant	MODERATE	c.1166T>C	p.Phe389Ser	0	2
2453147	*Rv2190c*	Rv2190c	1017	1.50744E-08	7	missense_variant	MODERATE	c.126C>A	p.Asp42Glu	10	6
2500964	*Rv2227*	Rv2227	1231	1.71959E-05	10	missense_variant	MODERATE	c.34C>G	p.Arg12Gly	8	4
2502324	*Rv2228c*	Rv2228c	1017	1.50744E-08	7	missense_variant	MODERATE	c.415G>A	p.Ala139Thr	10	6
2562288	*cdh*	Rv2289	381	1.75432E-13	299	missense_variant	MODERATE	c.614A>G	p.Asp205Gly	0	2
2586925	*Rv2314c*	Rv2314c	1017	1.50744E-08	7	synonymous_variant	LOW	c.366G>A	p.Gly122Gly	10	6
2595142	*rocD1*	Rv2322c	1017	1.50744E-08	7	missense_variant	MODERATE	c.223A>G	p.Thr75Ala	10	6
2605885	*mez*	Rv2332	1227	2.99092E-06	10	missense_variant	MODERATE	c.778G>C	p.Val260Leu	7	4
2610381	*cysE*	Rv2335	1231	1.71959E-05	10	missense_variant	MODERATE	c.650C>T	p.Pro217Leu	8	4
2614232	*Rv2337c*	Rv2337c	1019	6.34814E-06	7	upstream_gene_variant	MODIFIER	c.-1245G>T		14	6
2628893	*plcB*	Rv2350c	1223	1.08236E-05	10	frameshift_variant	HIGH	c.1426_1427insGTTCA	p.Ala476fs	4	3
2628895	*plcB*	Rv2350c	1222	1.14389E-06	10	frameshift_variant	HIGH	c.1424_1425insTGAACCACCTGACATGACCCCATCCTTTCCAAGAACTGGAGTCTCCGGACATGCCGGGG	p.Ala476fs	1	2
2662802	*mbtE*	Rv2380c	1019	6.34814E-06	7	synonymous_variant	LOW	c.4314C>T	p.Phe1438Phe	14	6
2677876	*mbtJ*	Rv2385	1227	2.99092E-06	10	missense_variant	MODERATE	c.148G>A	p.Asp50Asn	7	4
2701529	*lepA*	Rv2404c	667	7.17007E-11	217	missense_variant	MODERATE	c.1720T>G	p.Phe574Val	0	2
2714526	*eis*	Rv2416c	1231	1.71959E-05	10	frameshift_variant	HIGH	c.805_806delAC	p.Thr269fs	8	4
2720993	*Rv2424c*	Rv2424c	1017	1.50744E-08	7	missense_variant	MODERATE	c.785G>C	p.Gly262Ala	10	6
2733749	*rbsK*	Rv2436	1227	2.99092E-06	10	missense_variant	MODERATE	c.520G>A	p.Gly174Ser	7	4
2739546	*obg*	Rv2440c	1231	1.71959E-05	10	synonymous_variant	LOW	c.141C>G	p.Val47Val	8	4
2757836	*Rv2456c*	Rv2456c	1017	1.50744E-08	7	synonymous_variant	LOW	c.357T>G	p.Ala119Ala	10	6
2766789	*lipP*	Rv2463	667	7.17007E-11	217	missense_variant	MODERATE	c.1135G>T	p.Val379Phe	0	2
2774856	*aglA*	Rv2471	617	1.59682E-09	215	synonymous_variant	LOW	c.1293C>A	p.Gly431Gly	0	2
2775156	*aglA*	Rv2471	470	1.55984E-09	216	frameshift_variant	HIGH	c.1594_1595insAT	p.Leu532fs	0	2
2803670	*PE_PGRS43*	Rv2490c	893	1.94095E-09	7	missense_variant	MODERATE	c.2567A>G	p.Asp856Gly	9	6
2835894	*PE26*	Rv2519	1231	1.71959E-05	10	missense_variant	MODERATE	c.110C>T	p.Thr37Ile	8	4
2840109	*ldtB*	Rv2518c	1227	2.99092E-06	10	upstream_gene_variant	MODIFIER	c.-4774C>T		7	4
2842880	*fas*	Rv2524c	1017	1.50744E-08	7	synonymous_variant	LOW	c.6453C>T	p.Leu2151Leu	10	6
2859133	*efp*	Rv2534c	1017	1.50744E-08	7	missense_variant	MODERATE	c.158C>T	p.Thr53Met	10	6
2867088	*lppA*	Rv2543	1017	1.50744E-08	7	synonymous_variant	LOW	c.621G>C	p.Pro207Pro	10	6
2876938	*Rv2556c*	Rv2556c	1017	1.50744E-08	7	missense_variant	MODERATE	c.28C>T	p.Arg10Cys	10	6
2876955	*Rv2556c*	Rv2556c	391	7.33622E-13	15	missense_variant	MODERATE	c.11T>C	p.Val4Ala	0	2
2888827	*Rv2566*	Rv2566	1227	2.99092E-06	10	missense_variant	MODERATE	c.2455G>A	p.Gly819Ser	7	4
2947988	*Rv2621c*	Rv2621c	1017	1.50744E-08	7	synonymous_variant	LOW	c.571T>C	p.Leu191Leu	10	6
2964867	*PE_PGRS46*	Rv2634c	1017	1.50744E-08	7	upstream_gene_variant	MODIFIER	c.-2426G>A		10	6
2980684	*Rv2659c*	Rv2659c	1019	6.34814E-06	7	synonymous_variant	LOW	c.135C>T	p.Ile45Ile	14	6

2984903	*Rv2668*	Rv2668	1017	1.50744E-08	7	synonymous_variant	LOW	c.171C>T	p.Val57Val	10	6
3000401	*Rv2683*	Rv2683	1231	1.71959E-05	10	missense_variant	MODERATE	c.290G>A	p.Cys97Tyr	8	4
3001584	*arsA*	Rv2684	617	1.59682E-09	215	missense_variant	MODERATE	c.971C>T	p.Ser324Phe	0	2
3007119	*Rv2686c*	Rv2686c	1230	3.21937E-06	10	upstream_gene_variant	MODIFIER	c.-3191_-3082del		7	4
3042024	*Rv2729c*	Rv2729c	1017	1.50744E-08	7	missense_variant	MODERATE	c.452T>G	p.Leu151Arg	10	6
3046181	*Rv2733c*	Rv2733c	667	7.17007E-11	217	missense_variant	MODERATE	c.344G>A	p.Arg115His	0	2
3057998	*35kd_ag*	Rv2744c	1017	1.50744E-08	7	missense_variant	MODERATE	c.66G>T	p.Glu22Asp	10	6
3068497	*hsdM*	Rv2756c	1231	1.71959E-05	10	synonymous_variant	LOW	c.1587A>G	p.Glu529Glu	8	4
3079112	*thyA*	Rv2764c	1019	6.34814E-06	7	upstream_gene_variant	MODIFIER	c.-4641C>T		14	6
3089878	*pepR*	Rv2782c	1017	1.50744E-08	7	missense_variant	MODERATE	c.484G>A	p.Asp162Asn	10	6
3099391	*ltp1*	Rv2790c	470	1.55984E-09	216	missense_variant	MODERATE	c.779C>T	p.Thr260Ile	0	2
3141737	*ugpA*	Rv2835c	1231	1.71959E-05	10	synonymous_variant	LOW	c.486T>C	p.Asp162Asp	8	4
3142701	*dinF*	Rv2836c	1017	1.50744E-08	7	missense_variant	MODERATE	c.928C>T	p.Arg310Trp	10	6
3160421	*Rv2851c*	Rv2851c	391	7.33622E-13	15	missense_variant	MODERATE	c.101C>A	p.Ala34Asp	0	2
3177276	*glnA4*	Rv2860c	470	1.55984E-09	216	upstream_gene_variant	MODIFIER	c.-4276C>T		0	2
3193202	*Rv2877c*	Rv2877c	1231	1.71959E-05	10	upstream_gene_variant	MODIFIER	c.-4331C>T		8	4
3197045	*amiC*	Rv2888c	1017	1.50744E-08	7	missense_variant	MODERATE	c.1241T>C	p.Val414Ala	10	6
3213678	*lepB*	Rv2903c	1227	2.99092E-06	10	synonymous_variant	LOW	c.177C>G	p.Pro59Pro	7	4
3218997	*dacB2*	Rv2911	1231	1.71959E-05	10	missense_variant	MODERATE	c.659T>A	p.Leu220Gln	8	4
3257937	*ppsC*	Rv2933	1017	1.50744E-08	7	missense_variant	MODERATE	c.2253C>G	p.Phe751Leu	10	6
3287430	*mmpL7*	Rv2942	1231	1.71959E-05	10	synonymous_variant	LOW	c.2361C>T	p.His787His	8	4
3298532	*fadD22*	Rv2948c	1017	1.50744E-08	7	stop_gained	HIGH	c.1423C>T	p.Arg475*	10	6
3300005	*Rv2949c*	Rv2949c	1231	1.71959E-05	10	missense_variant	MODERATE	c.566G>T	p.Trp189Leu	8	4
3304104	*Rv2951c*	Rv2951c	1017	1.50744E-08	7	missense_variant	MODERATE	c.145C>T	p.Pro49Ser	10	6
3365184	*lppZ*	Rv3006	1019	6.34814E-06	7	missense_variant	MODERATE	c.476T>C	p.Ile159Thr	14	6
3368380	*gatB*	Rv3009c	1017	1.50744E-08	7	synonymous_variant	LOW	c.414G>T	p.Arg138Arg	10	6
3405338	*fecB*	Rv3044	1017	1.50744E-08	7	missense_variant	MODERATE	c.203C>A	p.Pro68Gln	10	6
3413574	*nrdE*	Rv3051c	1231	1.71959E-05	10	missense_variant	MODERATE	c.593C>G	p.Ala198Gly	8	4
3430006	*Rv3064c*	Rv3064c	1017	1.50744E-08	7	missense_variant	MODERATE	c.245C>T	p.Ser82Leu	10	6
3437020	*Rv3074*	Rv3074	1019	6.34814E-06	7	missense_variant	MODERATE	c.242G>A	p.Arg81Gln	14	6
3443266	*pknK*	Rv3080c	1017	1.50744E-08	7	missense_variant	MODERATE	c.2723C>T	p.Ser908Leu	10	6
3451186	*Rv3085*	Rv3085	1227	2.99092E-06	10	synonymous_variant	LOW	c.267C>T	p.Asn89Asn	7	4
3451440	*Rv3085*	Rv3085	1017	1.50744E-08	7	missense_variant	MODERATE	c.521T>G	p.Leu174Arg	10	6
3462822	*Rv3094c*	Rv3094c	1231	1.71959E-05	10	missense_variant	MODERATE	c.1070C>A	p.Ala357Glu	8	4
3538141	*Rv3169*	Rv3169	1017	1.50744E-08	7	missense_variant	MODERATE	c.904G>C	p.Gly302Arg	10	6
3540882	*Rv3172c*	Rv3172c	1231	1.71959E-05	10	splice_region_variant&stop_retained_variant	LOW	c.483A>G	p.Ter161Ter	8	4
3545427	*hpx*	Rv3171c	1227	2.99092E-06	10	upstream_gene_variant	MODIFIER	c.-4682A>G		7	4
3570896	*uvrD2*	Rv3198c	1231	1.71959E-05	10	synonymous_variant	LOW	c.316C>A	p.Arg106Arg	8	4
3575931	*Rv3201c*	Rv3201c	1017	1.50744E-08	7	missense_variant	MODERATE	c.1106A>C	p.Glu369Ala	10	6
3583543	*moeB1*	Rv3206c	1017	1.50744E-08	7	synonymous_variant	LOW	c.168C>T	p.Pro56Pro	10	6
3594831	*Rv3218*	Rv3218	391	7.33622E-13	15	missense_variant	MODERATE	c.364G>A	p.Asp122Asn	0	2
3604064	*aroA*	Rv3227	67	5.28411E-07	324	missense_variant	MODERATE	c.688G>T	p.Ala230Ser	0	1
3607420	*Rv3230c*	Rv3230c	1016	1.94095E-09	7	frameshift_variant	HIGH	c.834delC	p.Ser279fs	9	6
3640321	*manA*	Rv3255c	391	7.33622E-13	15	upstream_gene_variant	MODIFIER	c.-4054A>G		0	2
3684649	*atsB*	Rv3299c	1231	1.71959E-05	10	missense_variant	MODERATE	c.1315C>T	p.Arg439Trp	8	4

3710380	*vapC44*	Rv3320c	391	7.33622E-13	15	upstream_gene_variant	MODIFIER	c.-2311_-2310insACAGGGAGCTGTCCGAGAGGTTGACGGCACTGCCCGGGTGGGAGCTCGTCGATGGAAAGCTCCGCCACACATTTGGGTTTGGGAGCTTCGATCAATCCATGAAATTTGTGGCGAAGATCGCCGCCATCGCCGACAAATTCAATCATCACCCCGATATCTG		0	2
3721174	*Rv3333c*	Rv3333c	1231	1.71959E-05	10	upstream_gene_variant	MODIFIER	c.-392A>C		8	4
3724042	*Rv3337*	Rv3337	1231	1.71959E-05	10	stop_lost&splice_region_variant	HIGH	c.387A>G	p.Ter129Trpext*?	8	4
3725794	*icd1*	Rv3339c	1226	3.13154E-08	10	disruptive_inframe_insertion	MODERATE	c.50_51insTGAACCACCTGACATGACCCCATCCTTTCCAAGAACTGGAGTCTCCGGACATGCCGGGGCGGTTCACGA	p.Asp17_Gly18insGluProProAspMetThrProSerPheProArgThrGlyValSerGlyHisAlaGlyAlaValHisAsp	5	4
3734943	*PPE54*	Rv3343c	1017	1.50744E-08	7	missense_variant	MODERATE	c.1993A>C	p.Thr665Pro	10	6
3736108	*PPE54*	Rv3343c	1228	1.21361E-05	10	synonymous_variant	LOW	c.828T>C	p.Ile276Ile	8	4
3736828	*PPE54*	Rv3343c	1231	1.71959E-05	10	synonymous_variant	LOW	c.108C>A	p.Ala36Ala	8	4
3746219	*PPE55*	Rv3347c	631	0.00000078	281	synonymous_variant	LOW	c.6966G>A	p.Ala2322Ala	0	1
3766200	*PPE56*	Rv3350c	1017	1.50744E-08	7	synonymous_variant	LOW	c.903C>A	p.Gly301Gly	10	6
3770743	*PPE56*	Rv3350c	1231	1.71959E-05	10	upstream_gene_variant	MODIFIER	c.-3641A>G		8	4
3778338	*Rv3361c*	Rv3361c	391	7.33622E-13	15	upstream_gene_variant	MODIFIER	c.-4771C>T		0	2
3782763	*dnaE2*	Rv3370c	667	7.17007E-11	217	missense_variant	MODERATE	c.1978C>G	p.Pro660Ala	0	2
3784399	*dnaE2*	Rv3370c	617	1.59682E-09	215	synonymous_variant	LOW	c.342C>T	p.Ala114Ala	0	2
3794515	*dxs2*	Rv3379c	1017	1.50744E-08	7	frameshift_variant	HIGH	c.352dupG	p.Ala118fs	10	6
3818638	*Rv3401*	Rv3401	1019	6.34814E-06	7	synonymous_variant	LOW	c.597G>A	p.Glu199Glu	14	6
3819448	*Rv3401*	Rv3401	1227	2.99092E-06	10	frameshift_variant	HIGH	c.1410delG	p.His471fs	7	4
3844733	*Rv3422c*	Rv3422c	1231	1.71959E-05	10	upstream_gene_variant	MODIFIER	c.-4536T>G		8	4
3859614	*Rv3439c*	Rv3439c	1017	1.50744E-08	7	missense_variant	MODERATE	c.49G>A	p.Ala17Thr	10	6
3866167	*eccC4*	Rv3447c	1227	2.99092E-06	10	missense_variant	MODERATE	c.2072A>G	p.His691Arg	7	4
3872538	*eccC4*	Rv3447c	1017	1.50744E-08	7	upstream_gene_variant	MODIFIER	c.-4300C>T		10	6
3893944	*Rv3471c*	Rv3471c	631	0.00000078	281	upstream_gene_variant	MODIFIER	c.-4603C>T		0	1
3899672	*Rv3480c*	Rv3480c	1231	1.71959E-05	10	missense_variant	MODERATE	c.731G>A	p.Arg244His	8	4
3914154	*lprN*	Rv3495c	1227	2.99092E-06	10	synonymous_variant	LOW	c.381C>T	p.Thr127Thr	7	4
3936503	*PE_PGRS54*	Rv3508	1229	3.11448E-06	10	conservative_inframe_deletion	MODERATE	c.5512_5628del	p.Asn1838_Gly1876del	7	4
3941595	*PE_PGRS55*	Rv3511	631	0.00000078	281	missense_variant	MODERATE	c.1979C>A	p.Thr660Asn	0	1
3941604	*PE_PGRS55*	Rv3511	631	0.00000078	281	missense_variant	MODERATE	c.1988A>C	p.Lys663Thr	0	1
3941605	*PE_PGRS55*	Rv3511	631	0.00000078	281	missense_variant	MODERATE	c.1989A>C	p.Lys663Asn	0	1
3944340	*PE_PGRS56*	Rv3512	893	1.94095E-09	7	missense_variant	MODERATE	c.2617G>A	p.Asp873Asn	9	6
3944342	*PE_PGRS56*	Rv3512	893	1.94095E-09	7	disruptive_inframe_deletion	MODERATE	c.2634_2642delCAACGGCGG	p.Asn879_Gly881del	9	6
3944342	*PE_PGRS56*	Rv3512	893	1.94095E-09	7	synonymous_variant	LOW	c.2619T>C	p.Asp873Asp	9	6
3949345	*PE_PGRS57*	Rv3514	895	4.40448E-07	7	frameshift_variant	HIGH	c.3553_3554delAA	p.Asn1185fs	12	6
3963393	*kshA*	Rv3526	1017	1.50744E-08	7	missense_variant	MODERATE	c.955C>A	p.Leu319Met	10	6
3989051	*Rv3549c*	Rv3549c	1017	1.50744E-08	7	synonymous_variant	LOW	c.48G>A	p.Gly16Gly	10	6
4015697	*fadE34*	Rv3573c	1017	1.50744E-08	7	synonymous_variant	LOW	c.516C>T	p.Val172Val	10	6
4018615	*lppH*	Rv3576	1017	1.50744E-08	7	synonymous_variant	LOW	c.258G>A	p.Lys86Lys	10	6

4024520	*ispD*	Rv3582c	1019	6.34814E-06	7	synonymous_variant	LOW	c.520C>T	p.Leu174Leu	14	6
4058762	*Rv3618*	Rv3618	1231	1.71959E-05	10	missense_variant	MODERATE	c.65C>T	p.Thr22Ile	8	4
4067394	*hpt*	Rv3624c	1017	1.50744E-08	7	upstream_gene_variant	MODIFIER	c.-3490G>C		10	6
4076016	*Rv3636*	Rv3636	1231	1.71959E-05	10	missense_variant	MODERATE	c.265C>G	p.Arg89Gly	8	4
4117356	*Rv3677c*	Rv3677c	1227	2.99092E-06	10	missense_variant	MODERATE	c.697G>C	p.Glu233Gln	7	4
4117974	*Rv3677c*	Rv3677c	1017	1.50744E-08	7	synonymous_variant	LOW	c.79T>C	p.Leu27Leu	10	6
4130795	*Rv3689*	Rv3689	1017	1.50744E-08	7	missense_variant	MODERATE	c.439A>G	p.Ile147Val	10	6
4143453	*Rv3700c*	Rv3700c	1017	1.50744E-08	7	synonymous_variant	LOW	c.468G>A	p.Pro156Pro	10	6
4161437	*Rv3717*	Rv3717	1017	1.50744E-08	7	synonymous_variant	LOW	c.390G>A	p.Pro130Pro	10	6
4204224	*proV*	Rv3758c	470	1.55984E-09	216	missense_variant	MODERATE	c.194A>G	p.Asp65Gly	0	2
4228988	*glfT1*	Rv3782	1227	2.99092E-06	10	synonymous_variant	LOW	c.642G>A	p.Leu214Leu	7	4
4258229	*pks13*	Rv3800c	1017	1.50744E-08	7	missense_variant	MODERATE	c.2918T>C	p.Ile973Thr	10	6
4277325	*PE_PGRS62*	Rv3812	617	1.59682E-09	215	missense_variant	MODERATE	c.755C>G	p.Ala252Gly	0	2
4285042	*papA2*	Rv3820c	1019	6.34814E-06	7	missense_variant	MODERATE	c.784A>C	p.Met262Leu	14	6
4314255	*bfrB*	Rv3841	1017	1.50744E-08	7	synonymous_variant	LOW	c.78A>G	p.Ala26Ala	10	6
4338537	*gltB*	Rv3859c	1019	6.34814E-06	7	upstream_gene_variant	MODIFIER	c.-2456T>C		14	6
4343176	*espH*	Rv3867	1019	6.34814E-06	7	missense_variant	MODERATE	c.407A>C	p.Asp136Ala	14	6
4369452	*mycP2*	Rv3886c	1227	2.99092E-06	10	frameshift_variant	HIGH	c.718delG	p.Val240fs	7	4
4381079	*eccB2*	Rv3895c	1019	6.34814E-06	7	missense_variant	MODERATE	c.862A>G	p.Ser288Gly	14	6
4382055	*Rv3896c*	Rv3896c	1017	1.50744E-08	7	missense_variant	MODERATE	c.797C>T	p.Ala266Val	10	6
4407686	*gid*	Rv3919c	1227	2.99092E-06	10	stop_gained	HIGH	c.517G>T	p.Glu173*	7	4

Supplementary Table S5: Loci found by phyOverlap and their significant associations with different phenotypes in the Hanoi and KwaZulu-Natal study population

cluster[+] Hanoi	S315T[+] Hanoi	cluster[+]/S315T[+] Hanoi	cluster[+] KwaZulu-Natal	S315T[+] KwaZulu-Natal	cluster[+]/S315T[+] KwaZulu-Natal	Locus	Number of loci
No	**Yes**	No	No	No	No	Rv3428c-Rv3429, Rv0682, Rv0758, Rv3169, Rv3680-Rv3681c, Rv3647c-Rv3648c	6
No	No	No	**Yes**	No	**Yes**	Rv0006, Rv0747, Rv1452c	3
No	No	**Yes**	No	No	No	Rv3511, Rv0278c-Rv0279c, Rv1068c	3
No	**Yes**	No	No	**Yes**	No	Rv0667, Rv2043c	2
No	No	No	**Yes**	**Yes**	**Yes**	Rv1753c	1
No	No	No	**Yes**	**Yes**	No	RVnc0003-Rv2049c	1
No	No	No	No	**Yes**	No	Rv3806c	1
No	No	No	**Yes**	No	No	Rv1087	1
No	**Yes**	**Yes**	No	No	No	Rv3021c	1

cluster[+]: clustered (pairwise SNV difference between two isolates is no more than five SNVs); S315T[+]: harboring *katG*-S315T mutation; Hanoi: Hanoi's study cohort; KwaZulu-Natal: KwaZulu-Natal's study cohort

Supplementary Table S6: Associations between variants and spread of strains carrying *katG*-S315T mutation (cluster[+]/S315T[+]) by univariate and multivariate analyses using logistic regression models in Hanoi's study population

S6a) Between k-mers and cluster[+]/S315T[+]

Gene harboring k-mers	Pattern of k-mer presence/absence among all samples	Corresponding variants*	Odds Ratio	95% CI	Adjusted Odds Ratio**	95%CI
PPE18/19	rs14	Yes (SNV)	22.81	6.69-77.85	13.20	3.49-49.96
gid	rs230	Yes (SNV)	16.10	4.10-63.23	12.42	2.81-54.90
	rs138	No	0.07	0.02-0.22	0.05	0.01-0.19
	rs229	No	0.06	0.02-0.24	0.08	0.02-0.36
emrB	rs44	Yes (SNV)	18.55	5.61-61.29	11.98	3.24-44.29
Rv1588c	rs71	Yes (SNV)	0.05	0.02-0.18	0.08	0.02-0.31
pncA	rs186	Yes (SNV)	0.02	0.002-0.24	0.01	0.00-0.25

S6b) Between SNVs or small indels and cluster[+]/S315T[+]

Gene harboring k-mers	Variants	Odds Ratio	95% CI	Adjusted Odds Ratio**	95%CI
PPE18/19	SNV (E99A)	22.81	6.69-77.85	13.20	3.49-49.96
	SNV (A101T)	22.81	6.69-77.85	13.20	3.49-49.96
gid	SNV (E173*)	16.10	4.10-63.23	12.42	2.81-54.90
emrB	SNV (F508S)	20.47	6.10-68.62	12.59	3.40-26.58
Rv1588c	SNV (P34P)	20.47	6.10-68.62	12.59	3.40-26.58
pncA	SNV (Q141P)	48.62	4.14-571.15	128.86	4.02-4133.63

S6c) Between *PPE46/47*-like chimeric genes and cluster[+]/S315T[+]

Presence of *PPE46/47*-like chimeric gene	Proportion (%)	Odds Ratio	95% CI	Adjusted Odds Ratio**	95%CI
No	9/295 (3.1)	Reference	-	Referrence	-
Yes	6/37 (16.2)	6.15	2.05-18.43	6.81	2.13-21.72

* found by variant-based GWAS or deletion-based GWAS or BLAST search

** adjusted Odds Ratio: adjustment for patients' age, gender, living area, and Mtb lineage

95%CI: 95% confidence interval; SNV: single nucleotide variant; GWAS: genome wide association study
